# A paracrine circuit of IL-1**β**/IL-1R1 between myeloid and tumor cells drives genotype-dependent glioblastoma progression

**DOI:** 10.1172/JCI163802

**Published:** 2023-11-15

**Authors:** Zhihong Chen, Bruno Giotti, Milota Kaluzova, Montse Puigdelloses Vallcorba, Kavita Rawat, Gabrielle Price, Cameron J. Herting, Gonzalo Pinero, Simona Cristea, James L. Ross, James Ackley, Victor Maximov, Frank Szulzewsky, Wes Thomason, Mar Marquez-Ropero, Angelo Angione, Noah Nichols, Nadejda M. Tsankova, Franziska Michor, Dmitry M. Shayakhmetov, David H. Gutmann, Alexander M. Tsankov, Dolores Hambardzumyan

**Affiliations:** 1 Department of Oncological Sciences, The Tisch Cancer Institute, Mount Sinai Icahn School of Medicine, New York, New York, USA.; 2 Department of Pediatrics, AFLAC Cancer and Blood Disorders Center, Children’s Healthcare of Atlanta and Winship Cancer Institute, and; 3 Winship Cancer Institute, Emory University School of Medicine, Atlanta, Georgia, USA.; 4 Department of Genetics and Genomic Sciences, Icahn School of Medicine at Mount Sinai, New York, New York, USA.; 5 Department of Neurology, Rutgers University, New Brunswick, New Jersey, USA.; 6 Department of Data Science, Dana-Farber Cancer Institute, Boston, Massachusetts, USA.; 7 Department of Medicine, Brigham and Women’s Hospital, Boston, Massachusetts, USA.; 8 Department of Medicine, Harvard Medical School, Boston, Massachusetts, USA.; 9 Department of Stem Cell and Regenerative Biology, Harvard University, Cambridge, Massachusetts, USA.; 10 Emory University Department of Microbiology and Immunology, Emory Vaccine Center, Atlanta, Georgia, USA.; 11 Department of Human Biology, Fred Hutchinson Cancer Research Center, Seattle, Washington, USA.; 12 Department of Neurosurgery and; 13 Department of Pathology and Molecular and Cell-Based Medicine, Mount Sinai Icahn School of Medicine, New York, New York, USA.; 14 Department of Data Science, Dana-Farber Cancer Institute, Boston, Massachusetts, USA.; 15 Department of Biostatistics, Harvard T.H. Chan School of Public Health, Boston, Massachusetts, USA.; 16 Department of Stem Cell and Regenerative Biology, Harvard University, Cambridge, Massachusetts, USA.; 17 The Ludwig Center at Harvard, Boston, Massachusetts, USA.; 18 The Broad Institute of MIT and Harvard, Cambridge, Massachusetts, USA.; 19 Lowance Center for Human Immunology and Emory Vaccine Center, Department of Pediatrics, Emory University School of Medicine, Atlanta, Georgia, USA.; 20 Department of Neurology, Washington University School of Medicine, St. Louis, Missouri, USA.

**Keywords:** Immunology, Oncology, Brain cancer, Cancer immunotherapy, Macrophages

## Abstract

Monocytes and monocyte-derived macrophages (MDMs) from blood circulation infiltrate glioblastoma (GBM) and promote growth. Here, we show that *PDGFB*-driven GBM cells induce the expression of the potent proinflammatory cytokine IL-1β in MDM, which engages IL-1R1 in tumor cells, activates the NF-κB pathway, and subsequently leads to induction of monocyte chemoattractant proteins (MCPs). Thus, a feedforward paracrine circuit of IL-1β/IL-1R1 between tumors and MDM creates an interdependence driving *PDGFB*-driven GBM progression. Genetic loss or locally antagonizing IL-1β/IL-1R1 leads to reduced MDM infiltration, diminished tumor growth, and reduced exhausted CD8^+^ T cells and thereby extends the survival of tumor-bearing mice. In contrast to IL-1β, IL-1α exhibits antitumor effects. Genetic deletion of *Il1a/b* is associated with decreased recruitment of lymphoid cells and loss-of-interferon signaling in various immune populations and subsets of malignant cells and is associated with decreased survival time of *PDGFB*-driven tumor-bearing mice. In contrast to *PDGFB*-driven GBM, *Nf1*-silenced tumors have a constitutively active NF-κB pathway, which drives the expression of MCPs to recruit monocytes into tumors. These results indicate local antagonism of IL-1β could be considered as an effective therapy specifically for proneural GBM.

## Graphical abstract

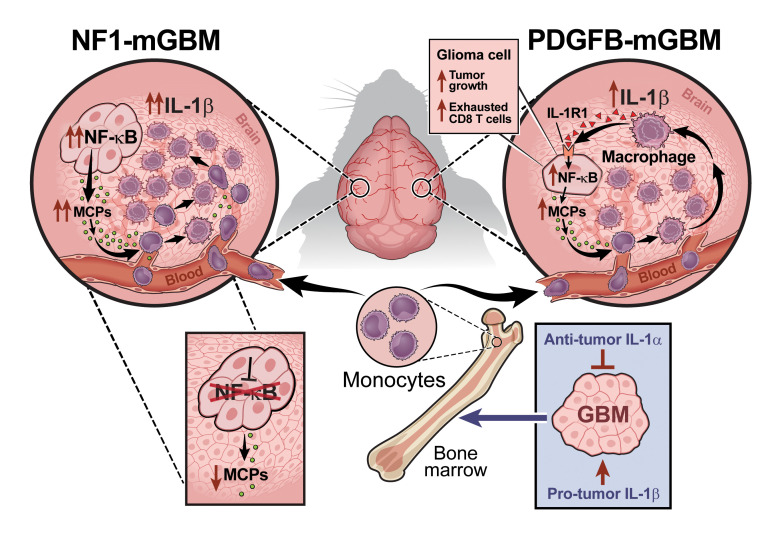

## Introduction

Glioblastoma (GBM) is the most prevalent and aggressive primary brain tumor in children and adults and has poor overall survival rates. Numerous therapies have entered clinical trials, with only temozolomide (TMZ) and radiotherapy (RT) modestly increasing median survival (1). One reason current antineoplastic therapies have not dramatically improved patient outcomes is the abundance of highly adaptive nonneoplastic cells in the tumor microenvironment (TME) that drive cancer progression. The nonneoplastic cells include tumor-associated macrophages (TAMs), which originate either from the bone marrow (bone marrow–derived myeloid cells [BMDMs]) or resident brain intrinsic microglia (MG) (2). Within this compartment, there is a high degree of intra- and intertumor heterogeneity, representing regional differences in composition and function, as well as non–mutually exclusive subclass differences among mesenchymal (MES), proneural (PN), and classical (CL) GBM (3, 4). Moreover, single-cell RNA-Seq (scRNA-Seq) analysis revealed that reciprocal interactions between TAMs and tumor cells can drive a transition of GBM into a MES-like cellular state (5). Thus, the resistance of heterogeneous GBM with multiple cellular states to current standard-of-care therapy may be improved with TAM-targeted immunotherapies (5, 6).

TAMs are generally regarded as “immunosuppressive” and are critical for glioma progression, as they release a wide array of growth factors, chemokines, and cytokines (7). One such cytokine produced by BMDMs is the master proinflammatory regulator IL-1 (IL-1 denotes both IL-1α and IL-1β), which induces GBM-associated vasogenic edema (8). Increased expression of IL-1α and IL-1β has been reported in numerous cancers, where their tumor-promoting roles have been established (9). Although IL-1α and IL-1β both signal through the same ubiquitously expressed receptor (IL-1R1) on all cells, the resulting effects on tumor biology can be distinct and tissue specific (10–12). Literature suggests the effects of IL-1β signaling, its downstream targets, and biological consequences are cell type specific (13, 14). Therefore, it is essential to understand the multifaceted roles of IL-1β in de novo GBM with regard to the whole animal whose immune microenvironment is intact.

To determine whether IL-1α and IL-1β have differential effects on GBM development and whether the IL-1β effect is tumor genotype dependent, we leveraged the RCAS/*Ntv-a* somatic cell type–specific gene-transfer system to create a series of genetically engineered mouse models (GEMM) representing the human PN and MES GBM molecular subtypes (15). Using these murine *PDGFB*-driven (*PDGFB* mGBM) and *Nf1*-silenced (*Nf1* mGBM) GBM models that show differential myeloid recruitment (16), we investigated how IL-1α and IL-1β individually contribute to tumor development in vitro and in vivo. In *PDGFB* mGBM, we describe a paracrine circuit in which GBM cells recruit and induce IL-1 expression in BMDMs, resulting in NF-κB pathway activation in GBM cells, increased expression of the monocyte chemoattractant proteins (MCPs), and increased chemotaxis of inflammatory monocytes. We demonstrate genetic loss of IL-1β leads to decreased inflammatory monocyte influx, reduced exhausted CD8^+^ T cells, and prolonged survival of mice. Similarly, pharmacological inhibition of IL-1β or IL-1R1 locally decreases intratumoral TAM content and prolongs survival of tumor-bearing mice. However, genetic deletion of both IL-1α and IL-1β results in decreased recruitment of lymphoid cells and loss of IFN signaling in immune cells, allowing the tumor to escape immune control and worsen survival. In contrast to *PDGFB* mGBM, IL-1β is not responsible for monocyte recruitment in MG-enriched *Nf1*-silenced mGBM due to constitutively active NF-κB signaling, which drives the expression of MCPs and recruits monocytes. Collectively, these findings establish a feedforward paracrine circuit of IL-1β/IL-1R1 between BMDMs and tumors that promotes *PDGFB* mGBM progression. Local antagonism of IL-1β could be considered an effective therapy for PN, but not *Nf1-*silenced GBM. These results demonstrate how GBM driver mutations differentially establish unique immune TMEs relevant to tumor pathogenesis.

## Results

### Increased IL1β expression in IDH1 WT GBM is associated with reduced patient survival.

**Figure 1:**
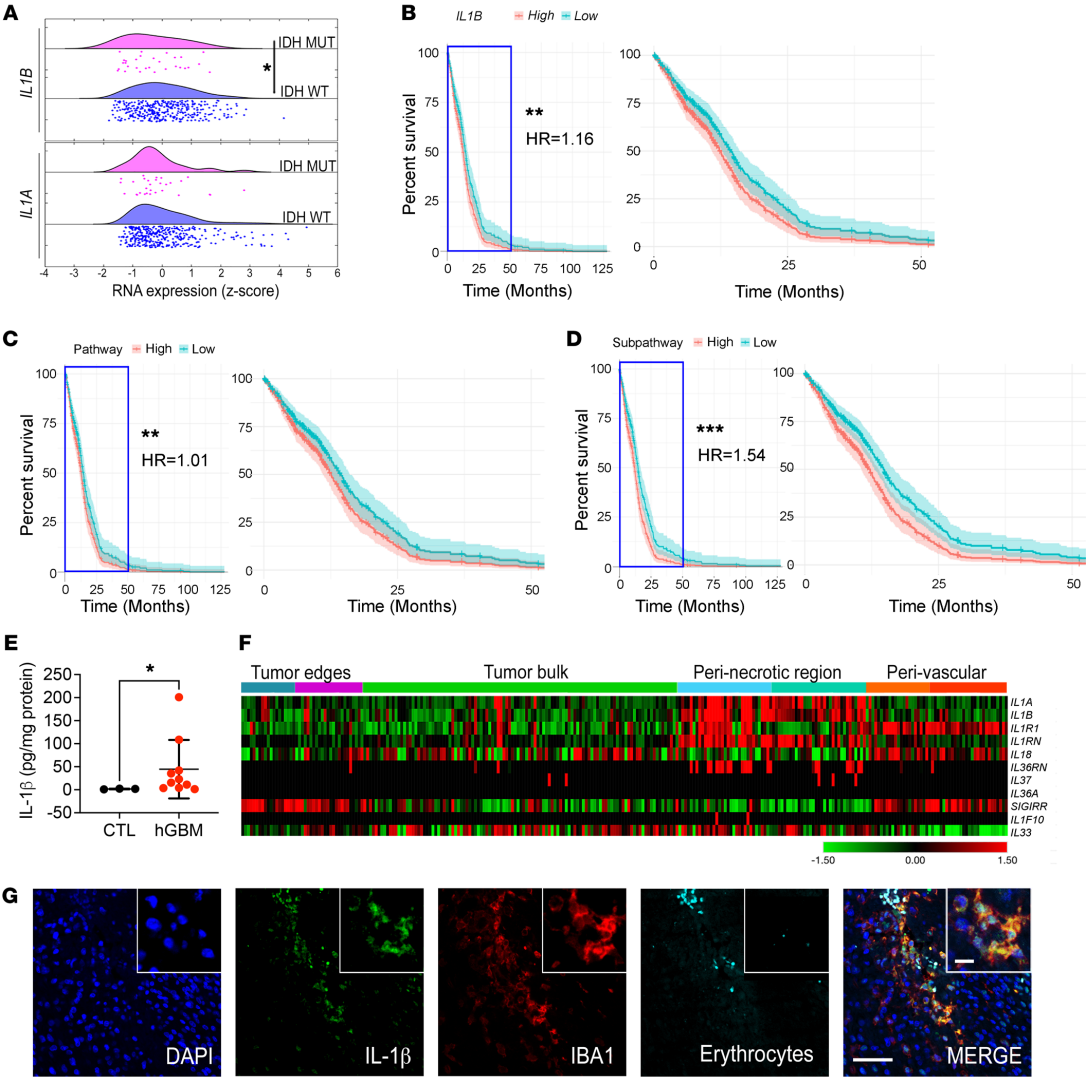
High *IL1B* expression in patients with IDH-WT GBM is associated with reduced survival. (**A**) *IL1A* and *IL1B* expression in IDH-WT (*n* = 372) and IDH-Mut (*n* = 30) patient samples from TCGA data sets. Unpaired Student’s *t* test. (**B**–**D**) Survival curves grouped by high and low total expression (relative to median) of the *IL1B* gene (**B**), IL-1 pathway (**C**), or a selected IL-1 subpathway (**D**, described in Supplemental Figure 1), fit using Cox’s proportional hazards regression models (zoomed curves on the right). *P* values and HRs were derived from the Cox’s proportional hazards model including expression as a continuous covariate and adjusting for sex and age. (**E**) IL-1β ELISA in human IDH-WT GBM samples (*n* =10). CTL, adjacent normal brain tissues (*n* = 3). Unpaired Student’s *t* test. (**F**) Expression of *IL1* family members in various regions of human GBM tissues as defined by the IVYGap database (*n* = 36: PN = 10, CL = 12, MES = 8, other = 6) following laser capture microdissection and RNA-Seq. (**G**) Representative images of immunofluorescence staining of IL-1β (green), IBA1 (red), erythrocytes (cyan), and nuclei (visualized with DAPI, blue). Scale bars: 100 μm; 20 μm (insets). *P < 0.05; **P < 0.01; ***P < 0.001.

Adult diffuse gliomas are divided into 2 groups based on the mutational status of isocitrate dehydrogenase 1 or 2 (*IDH1* and *IDH2*) genes. *IDH1/2* mutant (Mut) gliomas present as lower histologic grades and have better survival, but often transform to higher grades as the disease progresses. *IDH* WT gliomas are referred to as glioblastoma (GBM) and present in the highest histologic grade with the worst prognosis (17). We first evaluated *IL1A* and *IL1B* expression in IDH-WT and IDH-Mut samples using TCGA data sets via cBioPortal (18, 19). While there were no differences in *IL1A* expression, *IL1B* expression was higher in IDH-WT samples (Figure 1A; *P* < 0.05). To determine whether IL-1 and its signaling pathway affect survival of patients with IDH-WT GBM from TCGA, we used a Cox’s proportional hazards regression model to assess survival as a function of the following independent covariates: (a) each gene associated with IL1 signaling, (b) the entire pathway (specifically, the average normalized expression of all genes of the pathway, Supplemental Figure 1A; supplemental material available online with this article; https://doi.org/10.1172/JCI163802DS1), and (c) a subset of selected genes (the average normalized expression of *CASP1*, *IL1A*, *IL1B*, *IL1R1*, *IL1RAP*, *MYD88*, *TRAF6*, *TOLLIP*, *JUN*, *NFKB1*; highlighted in Supplemental Figure 1A).

A total of 372 IDH-WT patient samples for which covariate information (survival information, age, and sex) was available were included. We found that when controlled for age and sex, both *IL1B* (Figure 1B, hazard ratio [HR] = 1.16, *P* < 0.01) and IL1-related genes collectively serve as a significant adverse prognostic predictors of survival (Figure 1C, HR = 1.01, *P* < 0.01). Particularly, when we analyzed the IL-1 signaling subpathway of selected genes spanning the entire pathway, the HR increased to 1.54 (Figure 1D, *P* = 0.001), where 4 individual genes (*IL1B*, *IL1A*, *CASP1*, and *TOLLIP*) significantly influenced survival (Supplemental Figure 1, B–E). We also found IL1 pathway expression and any of its separate components does not affect survival differently depending on patient sex, as assessed by including an additional interaction term between gene expression and sex in the Cox’s proportional hazards model (Supplemental Figure 1, B–E). Our results differ from those of a recent report showing that *IL1B* expression only correlated with patient survival when the patients were stratified by sex. These differences may reflect the exclusion of sex as an explicit factor in their model, where males and females are treated independently (20).

Next, we confirmed increased IL-1β expression in microdissected IDH-WT human GBM tissues relative to adjacent normal brain tissues by ELISA (Figure 1E, patient information in Supplemental Table 3). To determine where within the tumor *IL1B* and its receptor (*IL1R1*) are expressed, we queried the IVY Glioblastoma Atlas Project (IVY GAP) database (https://glioblastoma.alleninstitute.org/) and found that *IL1B* and *IL1R1* are predominantly transcribed in perinecrotic and perivascular regions, whereas the tumor bulk contains comparatively little expression (Figure 1F) (21). Additionally, we found that the majority of the *IL1* family members, including *IL1A* and *IL1Ra*, are similarly enriched in perinecrotic/perivascular regions (Figure 1F), where we have previously shown that TAMs reside (2, 22). These results are further supported by a recent report demonstrating that *IL1B* is expressed in TAMs/MG by using scRNA-Seq from fresh GBM patient samples (23). We next costained human GBM tissue sections for IL-1β and IBA1 (pan-macrophage marker) and found that IL-1β colocalizes with IBA1 in perivascular regions, suggesting TAMs are a major source of IL-1β in human GBM (Figure 1G). To determine whether there are correlations between IL-1β expression levels and various T cell subsets, we immunostained a panel of human GBM samples representing known histologic subtypes with anti–IL-1β antibodies (Supplemental Figure 2, A and B) to correlate with T cell subsets from our recent publication (3). We observed no correlation between IL-1β expression and T cell subtype or content (Supplemental Figure 2C). It is important to note that these results may be affected by treatment of patients with dexamethasone, which is known to inhibit IL-1β (8).

### GBM cells induce IL-1 expression in BMDMs.

**Figure 2:**
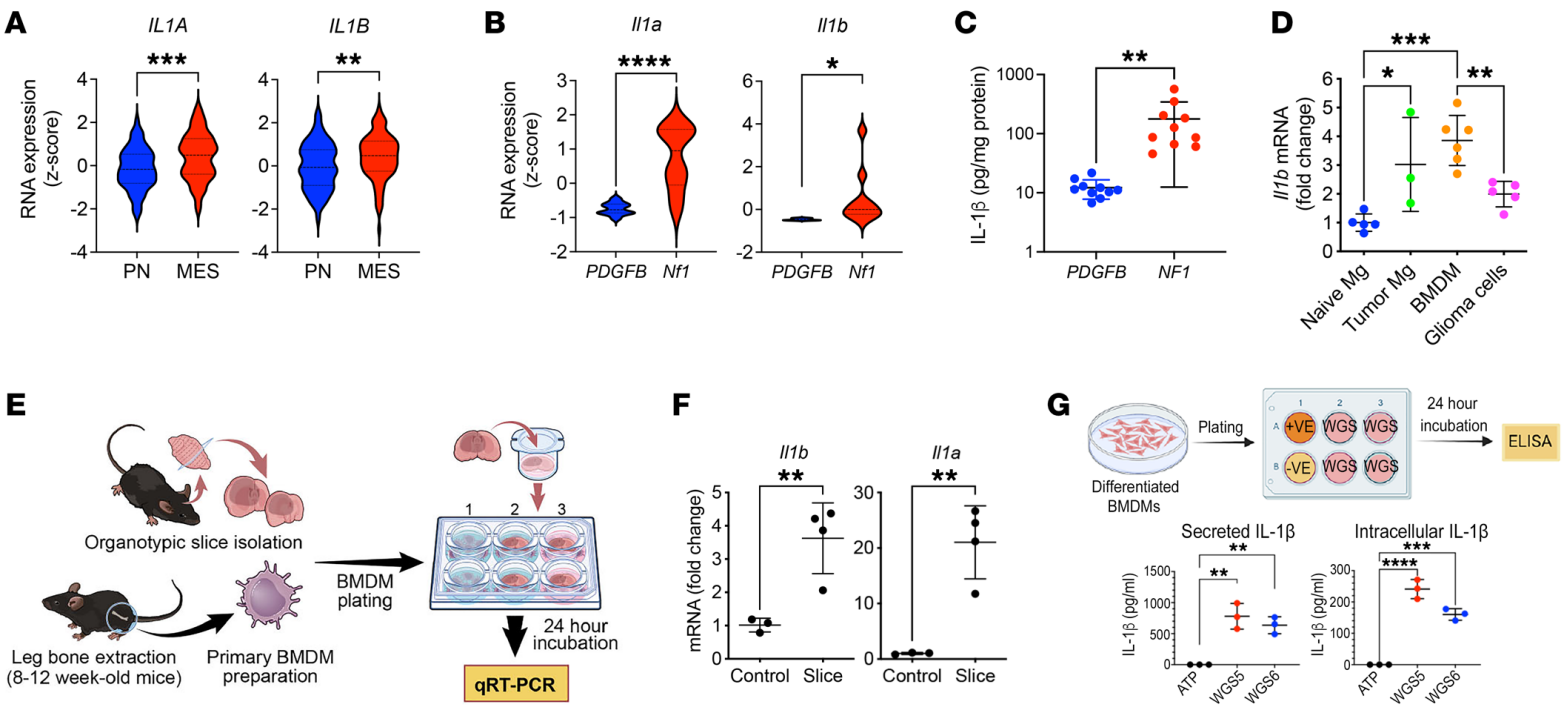
*IL1B* expression is increased in human MES GBM and *Nf1*-silenced murine GBM. (**A**) *IL1A* and *IL1B* RNA expression in PN (*n* = 69) and MES (*n* = 106) human GBM patient samples from TCGA. Two-tailed Student’s *t* test. (**B**) qPCR for *Il1a* and *Il1b* RNA expression from murine *PDGFB*-driven (*n* = 10) and *Nf1*-silenced (*n* = 10) GBM samples. Two-tailed Student’s *t* test. (**C**) IL-1β in *PDGFB*-driven (*n* = 10) and *Nf1*-silenced (*n* = 10) murine GBM tissues by ELISA. Two-tailed Student’s *t* test. (**D**) qPCR of *Il1b* expression in FACS-sorted cells from naive brain (*n* = 5) and *PDGFB*-driven tumors (*n* = 3 to 6). One-way ANOVA with Tukey’s post hoc comparisons. (**E**) Diagram illustrating the coculturing system of primary murine BMDM and *PDGFB*-driven tumor slices. (**F**) qPCR of *Il1a* and *Il1b* expression in BMDMs cocultured with tumor slices (*n* = 3 and 4 respectively). Two-tailed Student’s *t* test. (**G**) IL-1β expression from BMDM cocultured with primary PN glioma stem-like cells (WGS, *n* = 3 each group). One-way ANOVA with Tukey’s post hoc comparisons. *P < 0.05; **P < 0.01; ***P < 0.001; ****P < 0.0001.

Considering the colocalization of IL-1β and IBA1 in human GBM tissue, we next evaluated whether *IL1* expression levels differ between GBM subtypes. In light of previous studies demonstrating differences in TAM numbers and expression profiles between various human and murine GBM subtypes (3, 4, 16), we observed higher *IL1* expression levels in human MES, relative to PN GBM (Figure 2A, *P* < 0.01). In addition, similar results were observed in *Nf1*-silenced murine GBM (*Nf1* mGBM) (model of human MES GBM); *Nf1* expression was suppressed by shRNA compared with *PDGFB*-driven GBM (*PDGFB* mGBM) (model of human PN GBM) (Figure 2B, *P* < 0.05). Consistent with RNA expression results, IL-1β ELISA demonstrated *Nf1* mGBM had much higher IL-1β compared with *PDGFB* mGBM (Figure 2C, *P* < 0.01). This is not surprising, since both murine *Nf1* mGBM and human MES GBM exhibit a significantly higher number of TAMs (3, 16).

To identify the source of IL-1β, we used FACS to isolate tumor-associated MG, BMDMs, and glioma cells for *Il1b* real-time quantitative PCR. Compared with naive MG from control mice, all tumor-associated cell types showed increased *Il1b* mRNA (Figure 2D), with BMDM demonstrating the highest levels of *Il1b* mRNA (Figure 2D). We have previously demonstrated in contrast with LPS stimulation, which induces *Il1b* expression in both BMDM and MG cultures, organotypic tumor slices generated by *Cdkn2a* loss and PDGFB overexpression only induce *Il1b* expression in BMDM cocultures (8). Considering the previously suggested effect of p53 status on IL-1 response (24), we determined whether *Il1* expression in BMDM was induced by GBM tumor cells. Naive BMDMs were cocultured with primary *PDGFB* mGBM tumor slices with *p53* loss (Figure 2E). Both *Il1a* and *Il1b* were induced in BMDM by the tumor slices, suggesting the effect is independent of *p53* status (Figure 2F) (8). Next, we performed coculture experiments with *PDGFB*-driven primary tumor cells (GSCs) and BMDMs and measured both intracellular (pro–IL-1β) and secreted IL-1β. BMDM cocultured with tumor cells induced both IL-1β expression and secretion (Figure 2G); however, this effect was only observed using *PDGFB* mGBM tumors, but not *Nf1*-silenced mGBM cells (Supplemental Figure 3). Together, these results establish IL-1β is expressed in both human and murine GBMs, with greater levels observed in MES GBM, and is induced in a GBM subtype–specific manner in vitro.

### Il1b genetic ablation prolongs the survival of tumor-bearing mice.

**Figure 3:**
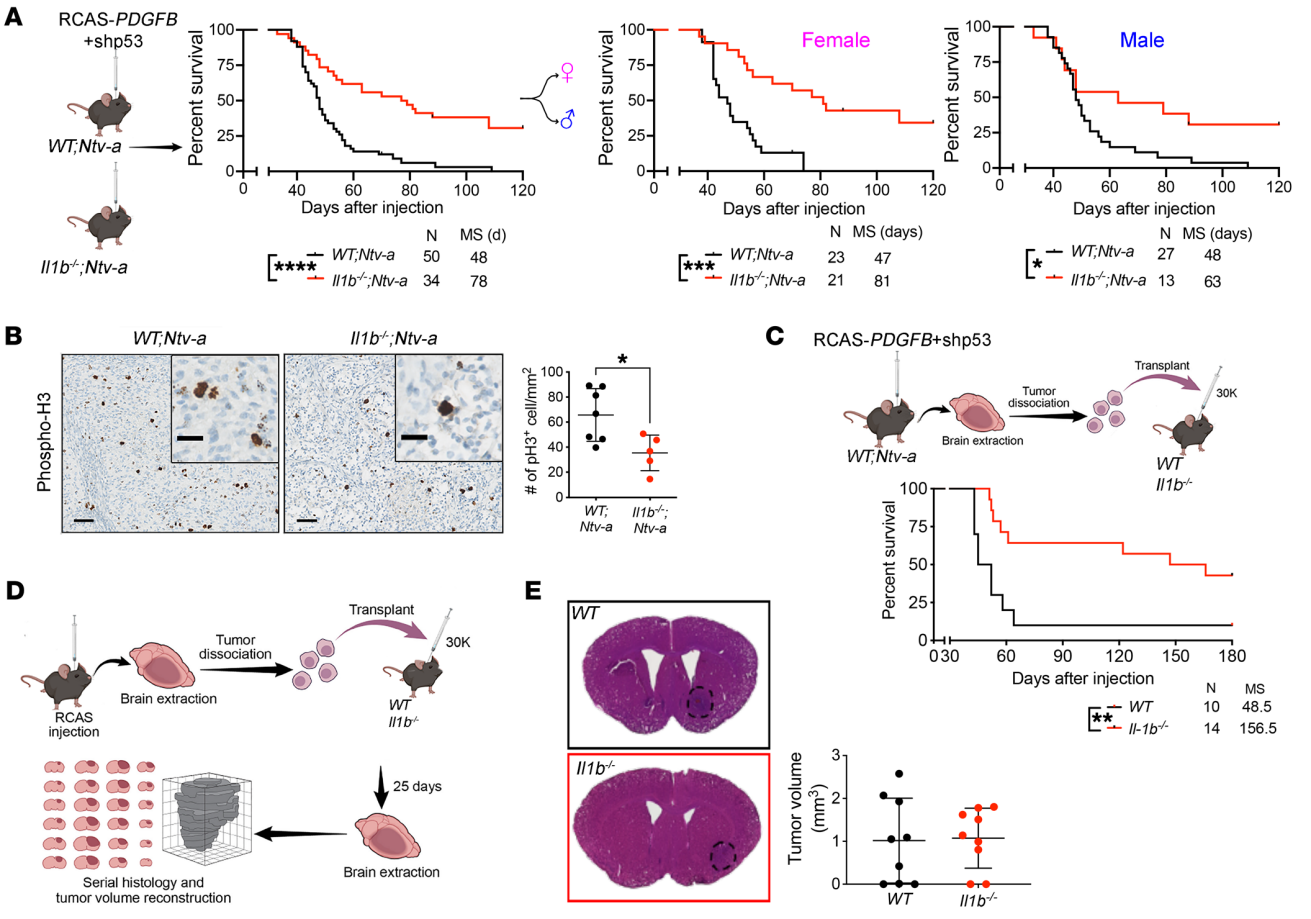
TME-derived IL-1β regulates *PDGFB*-driven murine GBM growth. (**A**) Kaplan-Meier survival curves of *PDGFB*-driven tumors generated in *WT;Ntv-a* and *Il1b^–/–^;Ntv-a* mice. Survival curves were also created stratified by sex. Curves were compared by log-rank (Mantel-Cox) test or Gehan-Breslow-Wilcoxon test (not shown). MS, median survival. *n* = number of mice. (**B**) Representative pH3 IHC images of *PDGFB*-driven tumors generated in *WT;Ntv-a* (*n* = 7) and *Il1b^–/–^;Ntv-a* mice (*n* = 5). Two-tailed Student’s *t* test. Scale bars: 50 μm; 20 μm (insets). (**C**) Kaplan-Meier survival curves of primary *PDGFB*-driven *Il1b* WT tumors in *Il1b^–/–^* and WT recipient animals. Mantel-Cox and Gehan-Breslow-Wilcoxon tests. (**D**) Schematic illustration of orthotopic transplant of primary *PDGFB*-driven *Il1* WT tumors into WT and *Il1b^–/–^* recipient animals to determine the effects on tumor volume during early tumor evolution. (**E**) Comparison of tumor volumes during early tumor evolution between tumors transplanted in WT (*n* = 9) and *Il1b^–/–^* (*n* = 9) recipient animals.*P < 0.05; ***P < 0.001; ****P < 0.0001.

To determine whether IL-1β is essential for GBM growth, we generated *PDGFB*-driven primary tumors in WT (*WT;Ntv-a)* and *Il1b*-KO (*Il1b^–/–^;Ntv-a)* mice by coinjecting RCAS-*PDGFB* and RCAS-shP53-*Rfp* (Figure 3A) (15). In KO mice where *Il1b* was absent in both the tumor and TME cells, *Il1b* loss prolonged the survival of *PDGFB*-driven tumor-bearing mice in a sex-independent manner (Figure 3A). This prolonged survival was associated with decreased numbers of proliferating (phospho-histone 3 [pH3] positive) cells in the tumors (Figure 3B).

Next, to distinguish between prolonged survival reflecting *Il1b* loss in tumor versus TME cells, we orthotopically transplanted 30,000 cells from primary *PDGFB* mGBM (*WT;Ntv-a* mice) into the striatum of *WT* and *Il1b^–/–^* mice (Figure 3C). Genetic *Il1b* ablation in the TME significantly prolonged the survival of tumor-bearing mice, recapitulating the results of the RCAS/*Ntv-a* system and suggesting that this beneficial effect of *Il1b* loss is a TME-driven effect (Figure 3C). To exclude defective tumor initiation in *Il1b^–/–^* mice, mice were euthanized at an earlier time point (25 days after tumor cell transplantation) and their brains serially sectioned for tumor volume estimates (8) (Figure 3D). We found that the tumor volumes were comparable between *WT* and *Il1b^–/–^* recipient mice at this early stage of tumor development, indicating that tumor initiation was not affected in *Il1b^–/–^* mice, but rather that reduced tumor growth in *Il1b^–/–^* mice is the driver of prolonged survival (Figure 3E).

### Il1 loss decreases inflammatory monocyte tumor infiltration.

**Figure 4:**
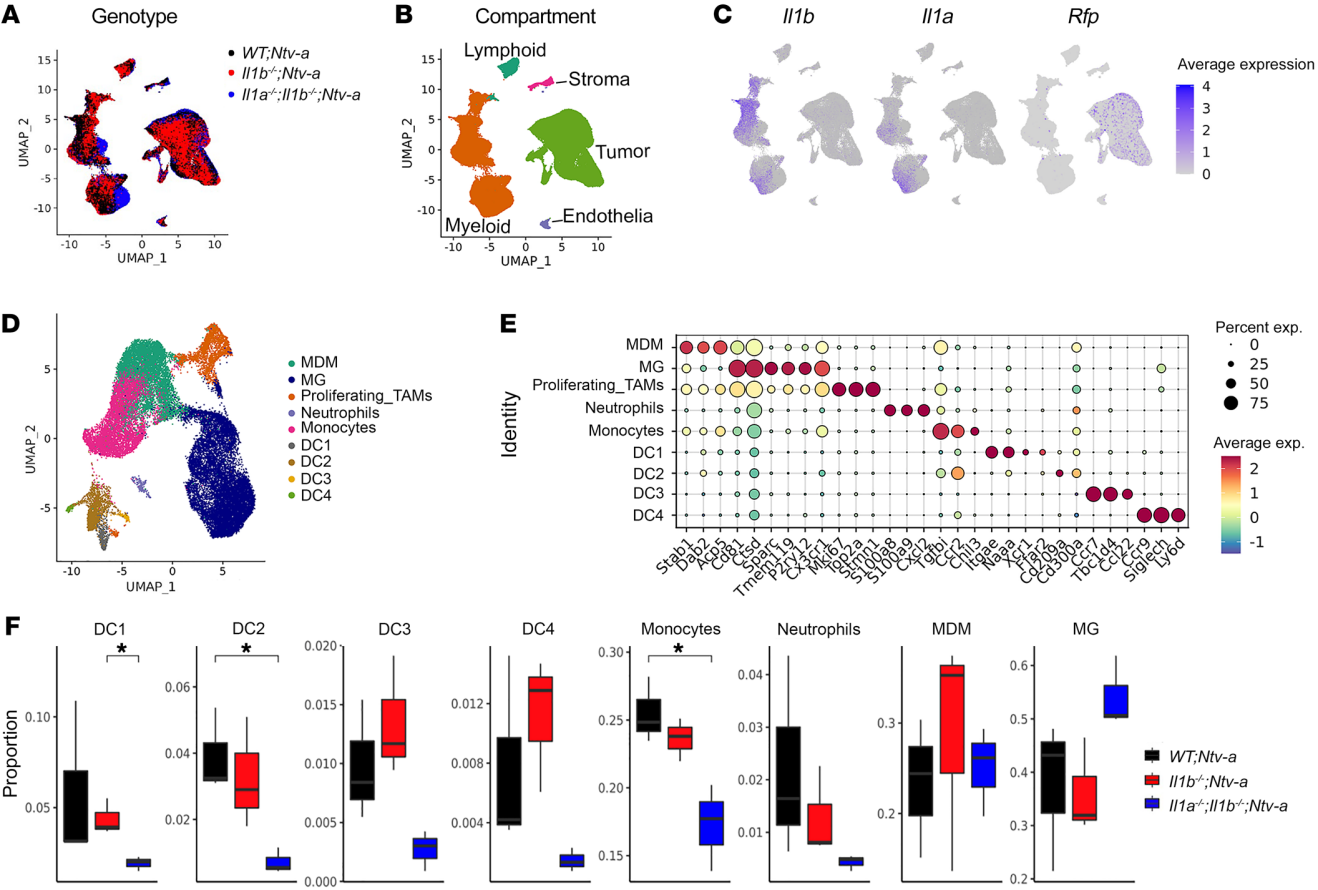
scRNA-Seq reveals reduction in inflammatory monocytes in *Il1b^–/–^;Ntv-a* mice. (**A**) UMAP dimensionality reduction of the scRNA-Seq data of tumors isolated from *WT;Ntv-a* (black, *n* = 3), *Il1b^–/–^;Ntv-a* (red, *n* = 3), and *Il1a^–/–^*;*Il1b^–/–^;Ntv-a* (blue, *n* = 3) mice. (**B**) UMAP of single cells in **A** colored by annotated cell class. (**C**) *Il1a*, *Il1b*, and *Rfp* expression overlayed on the same UMAP coordinates. (**D**) UMAP dimensionality reduction of myeloid cells in the tumors subclustered and colored by annotated myeloid subtypes. (**E**) Dot plot of selected marker genes defining different myeloid subtypes. (**F**) Composition of myeloid cell subpopulations in tumors generated in *WT;Ntv-a*, *Il1b^–/–^;Ntv-a* and *Il1a^–/–^*;*Il1b^–/–^;Ntv-a* mice. Two-tailed Student’s *t* test.*P < 0.05.

To determine whether there is a functional redundancy between IL-1α and IL-1β, we analyzed tumors isolated from *WT;Ntv-a*, *Il1b^–/–^;Ntv-a*, and *Il1a^–/–^;Il1b^–/–^;Ntv-a* mice at humane end points by scRNA-Seq (Figure 4A and Supplemental Figure 4, A–G). Unsupervised clustering identified 5 major cell classes in the tumors — lymphoid and myeloid immune cells, stromal cells, endothelial cells, and tumor cells (Figure 4B) — with myeloid cells accounting for the majority of the nonmalignant cell population, in agreement with our previous work (2). The scRNA-Seq data demonstrated that myeloid cells were the major producers of *Il1a* and *Il1b*, while the *Rfp^+^* (red fluorescent protein, result of the RCAS-shp53-*Rfp*) transformed neoplastic cells transcribe fewer of these cytokines (Figure 4C), consistent with our immunostaining results (Figure 1G).

We subclustered and classified the myeloid cells into phenotypic and functional subgroups (Figure 4D), with de novo annotations based on their expression profiles of signature genes (Figure 4E). We identified 2 TAM populations based on their ontogeny, namely monocyte-derived macrophages (MDMs) and MG (Figure 4E). We found the most significant changes occurred in the number of DC1s, DC2s, and monocytes, while other cell types varied only marginally among these 3 genotypes (Figure 4F). Specifically, we found a reduction in the relative abundance of monocytes in *Il1b^–/–^;Ntv-a* compared with *WT;Ntv-a* mice, which was more pronounced in the *Il1a^–/–^;Il1b^–/–^;Ntv-a* double-KO (DKO) mice (Figure 4F).

**Figure 5:**
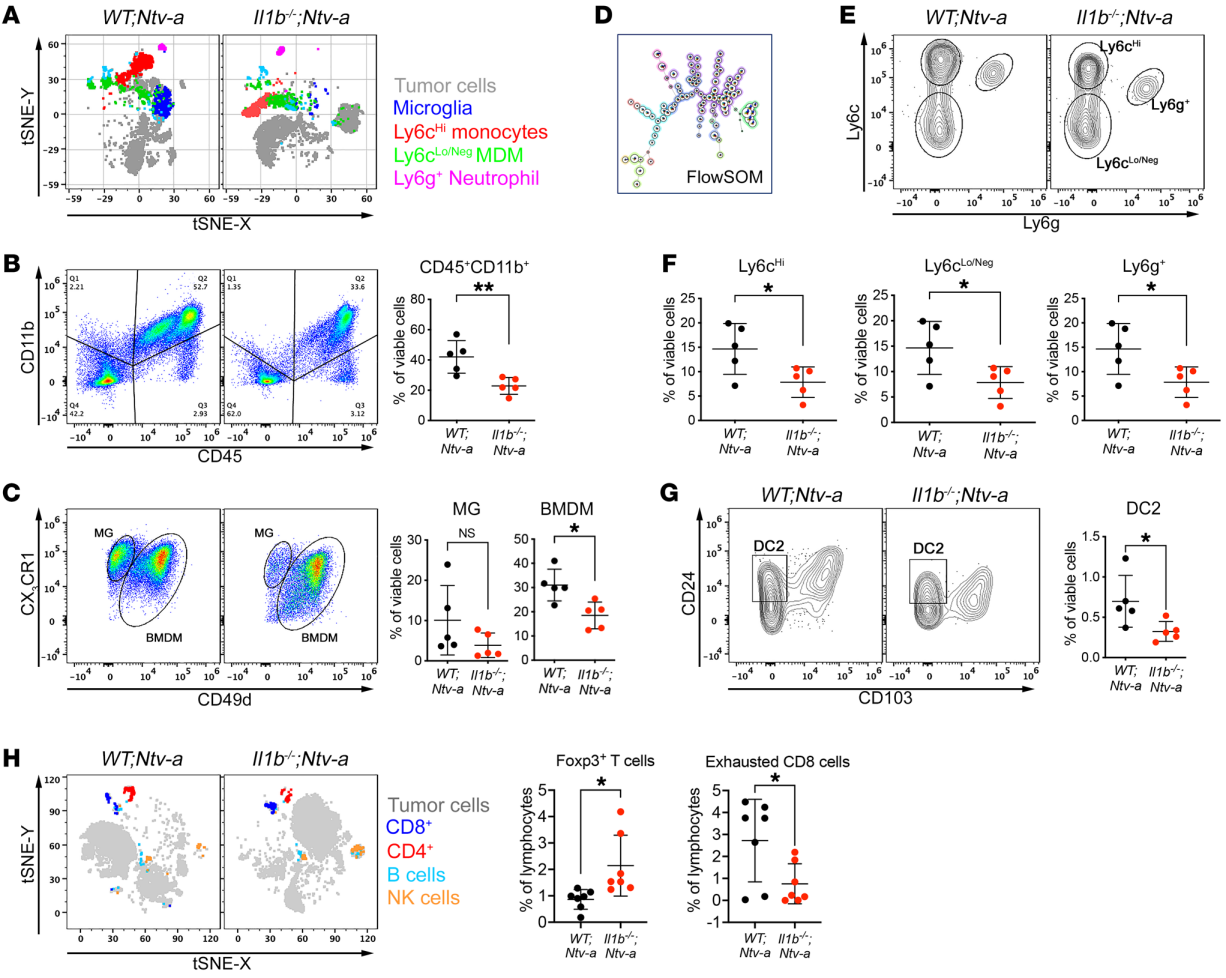
*Il1b* ablation reduces the influx of inflammatory monocytes and DC2s into tumors. (**A**) t-Distributed stochastic neighbor embedding (tSNE) plots of spectral flow cytometry illustrating the tumor cell/myeloid composition in *WT;Ntv-a* (*n* = 5) and *Il1b^–/–^;Ntv-a* mice (*n* = 5) bearing *PDGFB*-driven GBM. (**B** and **C**) Gating strategy for myeloid cells, discriminating between resident brain MG and BMDM, with corresponding quantification dot graphs. Two-tailed Student’s *t* test. (**D**) Illustration of FlowSOM differentiating subtypes of BMDMs. (**E**) Representative contour plots for gating monocytes (Ly6c^hi^), differentiated macrophages (Ly6c^lo/Neg^), and neutrophils (Ly6g^+^). (**F**) Quantification of myeloid cell subtypes depicted in **E**. Two-tailed Student’s *t* test. (**G**) Representative plots and quantification of DC2 populations in *WT;Ntv-a* and *Il1b^–/–^;Ntv-a* mice bearing *PDGFB*-driven GBM. Two-tailed Student’s *t* test. (**H**) tSNE plots and quantification of lymphoid cells by spectral flow cytometry (*n* = 7 for both groups). Two-tailed Student’s *t* test.*P < 0.05; **P < 0.01.

To complement and corroborate the scRNA-Seq data, we analyzed tumors at end points of survival experiments using spectral flow cytometry (Figure 5) to distinguish among inflammatory monocytes (CD11b^+^CD45^hi^Ly6c^hi^Ly6g^Neg^CD49d^+^), BMDMs (CD11b^+^CD45^hi^CX_3_CR1^+/–^CD49d^+^), MG (CD11b^+^CD45^lo^CX_3_CR1^+^Ly6c^Neg^Ly6g^Neg^CD49d^Neg^), and neutrophils (CD11b^+^CD45^+^Ly6c^+^Ly6g^+^CD49d^+^) (Figure 5A, gating strategy shown in Supplemental Figure 5). There was a significant reduction in total myeloid cells (CD45^+^CD11b^+^) in tumors from *Il1b^–/–^;Ntv-a* mice relative to *WT;Ntv-a* mice (Figure 5B, *P* < 0.01). Among these CD11b^+^ myeloid cells, MG abundance was not decreased in *Il1b^–/–^;Ntv-a* mice. However, the abundance of infiltrating BMDMs was reduced in *Il1b^–/–^;Ntv-a* mice (Figure 5C, *P* < 0.05). Using FlowSOM (Figure 5D) to identify subpopulations within the BMDM compartment (gating strategy illustrated in Figure 5E), we found a significant decrease in Ly6c^hi^ (newly infiltrated inflammatory monocytes) in *Il1b^–/–^;Ntv-a* mice, without any change in Ly6c^lo/Neg^ (differentiated macrophages) and Ly6g^+^ neutrophils (Figure 5F), closely reflecting the scRNA-Seq data (Figure 4F). Similarly, we detected a reduction in CD24^+^CD103^–^ DC2s (Figure 5G, gating strategy shown in Supplemental Figure 6) in tumors generated in *Il1b^–/–^;Ntv-a* mice compared with *WT;Ntv-a* mice, with no change in DC1s (Supplemental Figure 6).

To determine whether this reduction in Ly6c^hi^ monocytes in the tumors reflected fewer Ly6c^hi^ monocytes in circulation, we enumerated cell numbers in whole blood of mice using flow cytometry. We found no difference in the myeloid cells between tumor-bearing *WT;Ntv-a* and *Il1b^–/–^;Ntv-a* mice (Supplemental Figure 7). These data demonstrate IL-1β deficiency selectively reduces monocyte infiltration to the tumor from the periphery.

Next, we performed in-depth analysis on the lymphocyte compartment by scRNA-Seq (Supplemental Figure 8) and spectral flow cytometry (Supplemental Figures 9 and 10). Lymphoid cells account for a small portion of infiltrating cells in GBM, consistent with numerous previous studies (25, 26). We found a slight increase of Foxp3^+^ Tregs by both scRNA-Seq and flow cytometry in *Il1b^–/–^;Ntv-a* versus *WT;Ntv-a* mice (Supplemental Figure 8 and Figure 5H, *P* < 0.05) and a significant decrease in the amount of exhausted (Tim3^+^PD-1^+^) CD8^+^ T cells (Figure 5H, *P* < 0.05), but not in other cell types (Supplemental Figure 10). Since the number of Tregs in human GBMs has been shown to be prognostically neutral (27), and considering clinical significance of severe T cell exhaustion shown in GBM (28), although with a small footprint in the tumor, this reduction in exhausted CD8^+^ T cells may be an important contributor to the prolonged survival of the *Il1b^–/–^;Ntv-a* mice.

### IL-1β increases monocyte recruitment via tumor cell expression of MCPs.

As shown above, reduced inflammatory monocyte infiltration is one of the most prominent features observed in tumors generated in *Il1b^–/–^;Ntv-a* mice. This effect closely aligns with our previous findings demonstrating increased *Il1b* expression in tumor-bearing *Cx3cr1^–/–^* mice, which have increased inflammatory monocytes and shortened survival times (29). In addition, primary *PDGFB*-driven tumor cells increase CCL2 expression in response to recombinant IL-1β (rIL-1β) (29). However, genetic *Ccl2* deletion did not reduce TAM content in *PDGFB* mGBM (2).

In contrast to *Ccl2*, *Il1b* loss was sufficient to reduce both inflammatory monocytes and total TAM numbers in *PDGFB* mGBM, suggesting other MCP family members, such as CCL7, CCL8, and CCL12 (or CCL13 in humans), could be essential for mediating inflammatory monocyte GBM infiltration as we have recently shown (30). To test this hypothesis, we analyzed IL-1β induction of all 4 MCP members. We found that all MCP members were increased in primary *PDGFB* mGBM relative to normal brain, and all but one (CCL8) was increased in primary *Nf1* mGBM (Supplemental Figure 11A, *P* < 0.05). We examined this induction under 2 different culture conditions for (a) enriching glioma stem cells (GSCs) grown in a serum-free neural stem cell medium supplemented with EGF and βFGF (termed neutrosphere condition [NSC]), and (b) bulk tumor cells grown in 10% FBS in DMEM medium (termed differentiation condition, or FBS) (29) (Supplemental Figure 11B). We found freshly sorted *PDGFB* tumor cells expressed higher levels of *Ccl2*, *Ccl7*, *Ccl8*, and *Ccl12* relative to the same cells maintained in vitro (up to 6 passages) under both GSC and FBS conditions (Supplemental Figure 11C, *P* < 0.05). Although amounts of *Ccl12* mRNA levels did not reach statistical significance, they were higher in fresh isolates than in cultured cells (Supplemental Figure 11C). These observations suggest stromal support might be essential for *PDGFB*-driven tumor cells to produce MCPs.

To examine this potential explanation, we grew freshly isolated *PDGFB*-driven tumor cells under NSC and FBS conditions and measured MCP levels in the supernatant by ELISA at passage 1 (P1). While MCPs were expressed under both NSC and FBS conditions at P1 (Supplemental Figure 11D), at later P6, *PDGFB*-driven primary cells had reduced MCP expression, while *Nf1*-silenced cells showed high expression regardless of the number of passages (Supplemental Figure 11E), indicating that *Nf1*-silenced tumor cells are less reliant on stromal support for MCP induction, which is consistent with the high levels of IL-1β in the tumor cells of this GBM molecular subtype (MES).

Based on these observations, we hypothesized supplementing stroma-derived factors, such as IL-1β, could restore *PDGFB*-driven (PN), but not *Nf1*-silenced (MES), tumor cell production of MCPs. As such, we added rIL-1β (100 pM) to tumor cell cultures under both NSC and FBS conditions. While IL-1β stimulation had no effect on RNA or protein expression in *Nf1*-silenced tumor cells (Supplemental Figure 12A, B), MCP RNA and protein expression levels increased in *PDGFB* GBM cells under both NSC and FBS conditions (Supplemental Figure 12, C and D). Together, these data support that stroma-derived IL-1β mediates monocyte infiltration through the induction of MCP production in *PDGFB* mGBM cells.

### IL-1β activates NF-κB signaling in tumor cells in a GBM subtype–specific manner.

**Figure 6:**
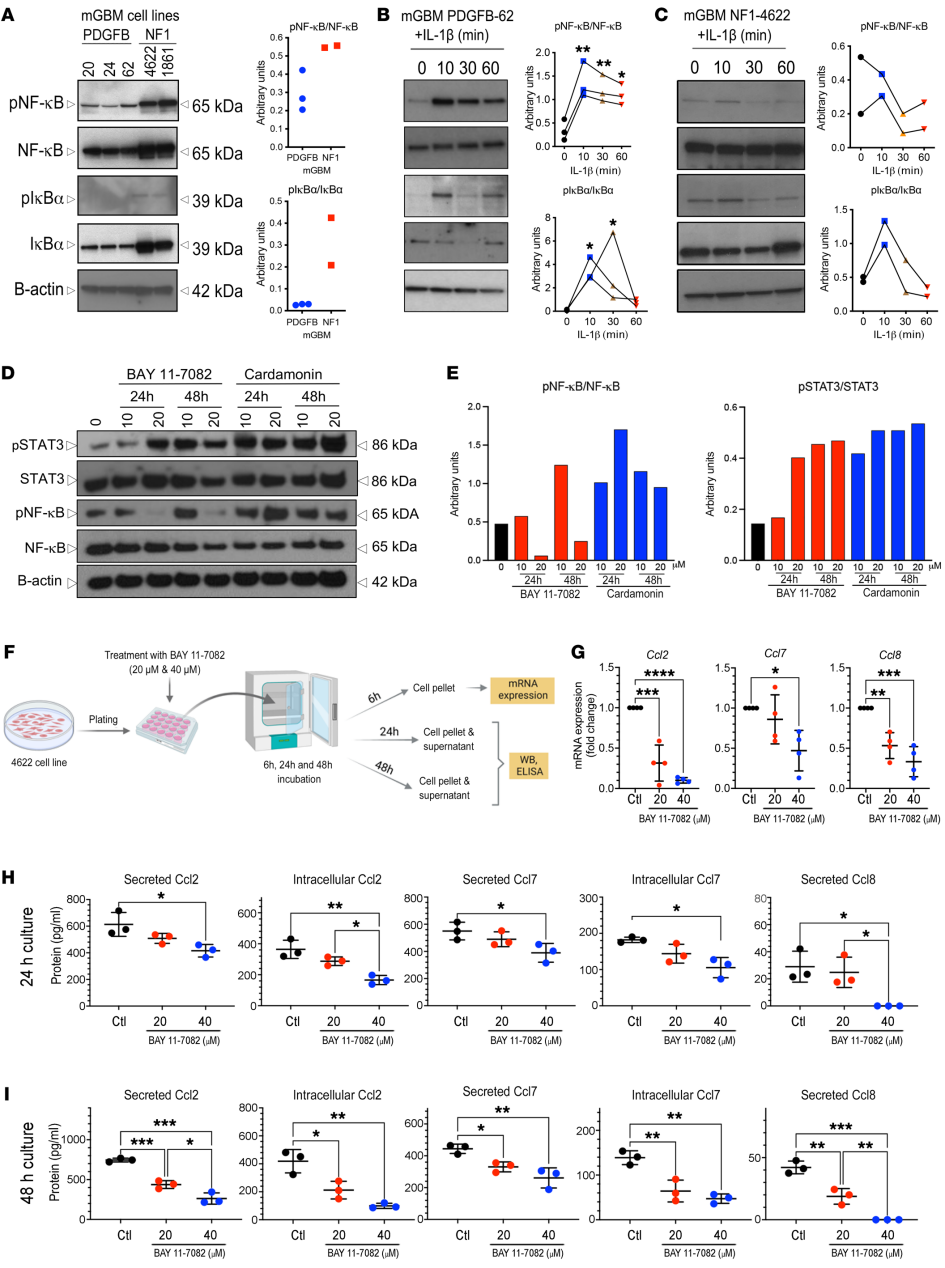
IL-1β induces NF-κB pathway activation in *PDGFB*-driven GBM cell cultures. (**A**) Immunoblot showing NF-κB pathway components in both *PDGFB-*driven (*n* = 3) and *Nf1*-silenced (*n* = 2) mGBM cultures in vitro. (**B** and **C**) NF-κB pathway components in response to IL-1β treatment of *PDGFB-*driven GBM cells (**B**) or *Nf1*-silenced GBM cells (**C**) in FBS-containing medium. Quantification with triplicate experiments. ANOVA test. *n* = 3; *n* = 2, respectively. (**D**) Western blot and quantifications (**E**) showing STAT-3 and NF-κB phosphorylation in MES cell lines in the presence of NF-κB pathway inhibitors. (**F**) Schematic illustration of MES cell line cultured in the presence of an NF-κB pathway inhibitor with MCP expression examined by quantitative reverse-transcriptase PCR (qRT-PCR) or ELISA. (**G**) *MCP* mRNA expression examined by qRT-PCR. One-way ANOVA with Tukey’s post hoc comparisons. *n* = 4 for each group. (**H**) Expression of MCPs at the protein level examined by ELISA for 24 hours (**H**) or 48 hours (**I**) after NF-κB inhibitor treatment. One-way ANOVA with Tukey’s post hoc comparisons. *n* = 3 for each group. One-way ANOVA with Tukey’s post-hoc comparisons.*P < 0.05; **P < 0.01; ***P < 0.001; ****P < 0.0001.

Based on prior studies implicating the NF-κB pathway in IL-1β–mediated signal transduction, we analyzed this pathway in *PDGFB* and *Nf1* mGBM tumor cell cultures in vitro. For *PDGFB* tumors, we used primary cells freshly isolated from excised tumors (P1 to P3). For *Nf1*-silenced tumors, we used murine GBM lines (lines 1816 and 4622), which lack *Nf1* and *Tp53* expression (31, 32). We chose these lines because *Nf1* mGBM cells are difficult to maintain in vitro. *Nf1* mGBM cells have increased phosphorylation (activation) of NF-κB pathway intermediates relative to *PDGFB* mGBM cells (Figure 6A), consistent with a recent study showing that MES GBM GSCs exhibit constitutively active NF-κB signaling (33). We next evaluated the effect of IL-1β (100 pM) on NF-κB pathway activation in NSC or FBS *PDGFB* and *Nf1* GBM cell cultures. While IL-1β induced NF-κB activation under both FBS (Figure 6B, *P* < 0.05) and NSC (Supplemental Figure 13) conditions in *PDGFB* mGBM, IL-1β stimulation did not induce NF-κB pathway activation in *Nf1* mGBM cells (Figure 6C). These results indicate *PDGFB* and *Nf1* mGBM cells intrinsically differ in NF-κB pathway signaling and in response to IL-1β stimulation. While *PDGFB* mGBM cells secrete MCPs in response to IL-1β, *Nf1*-silenced mGBM cells reach a plateau of MCP production, even in the absence of IL-1β stimulation. We interpret this to signify that *Nf1*-silenced mGBM cells exhibit constitutive NF-κB signaling. This was further explored using 2 commercially available NF-κB inhibitors, BAY 11-7082 and cardamonin. While NF-κB phosphorylation was reduced using 20 μM BAY 11-7082, this was not the case following cardamonin treatment. Interestingly, we observed increased levels of STAT3 phosphorylation when NF-κB phosphorylation was decreased with BAY 11-7082 (Figure 6, D and E). To determine whether inhibition of NF-κB phosphorylation changes MCP expression, we performed quantitative PCR (qPCR) after 6 hours of 20 μM and 40 μM BAY 11-7082 treatment. Inhibition of NF-κB phosphorylation was accompanied by reduced *Ccl2*, *Ccl8*, and *Ccl7* RNA expression (Figure 6, F and G) as well as decreased secreted and intracellular protein levels, as determined by ELISA (Figure 6, H and I). These results demonstrate that *Nf1*-silenced and *PDGFB* mGBM cells use different mechanisms to regulate MCP expression.

**Figure 7:**
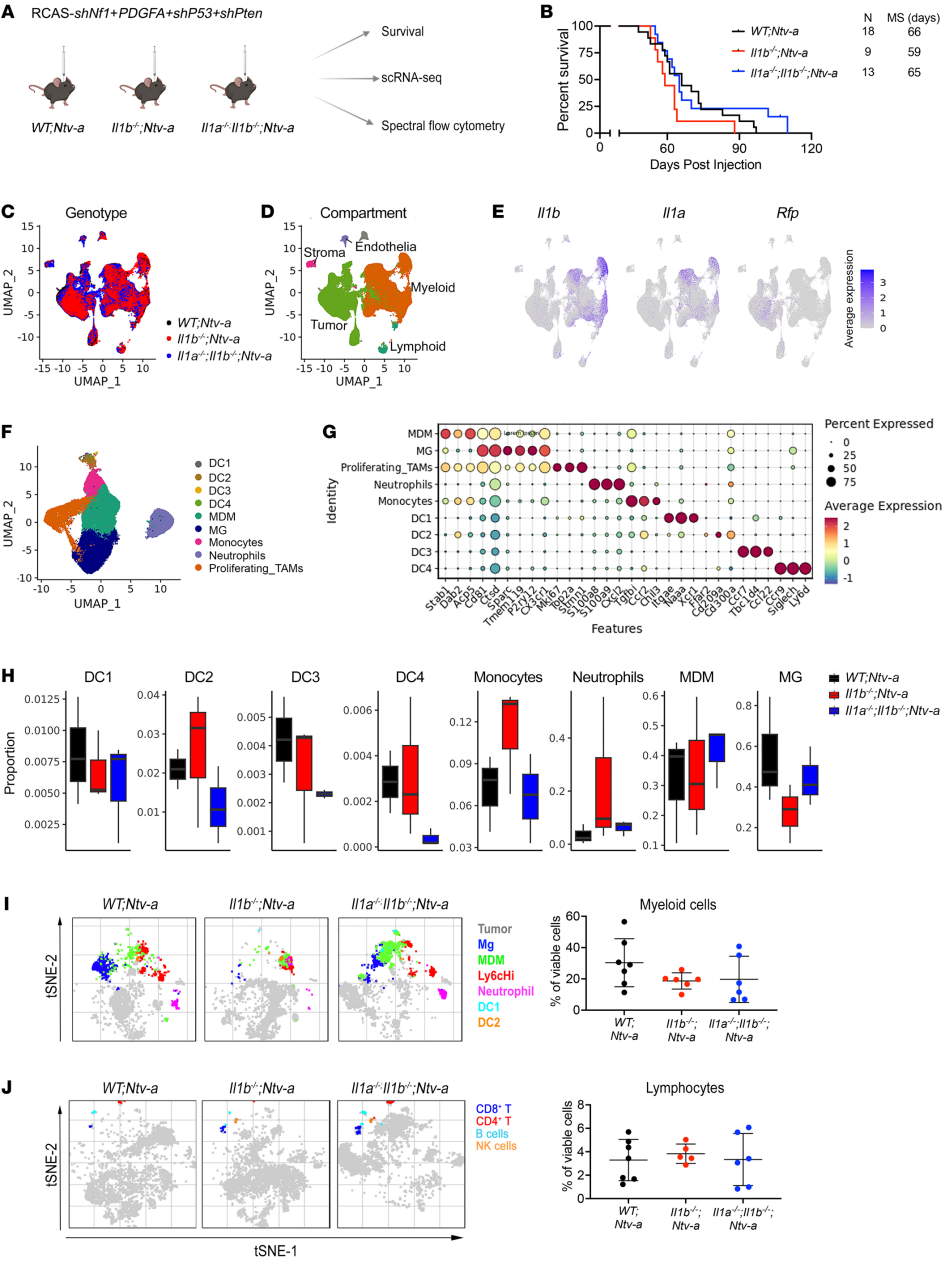
scRNA-Seq and Aurora immune phenotyping of *Nf1* mGBM generated in *Il1b^–/–^;Ntv-a* and *Il1a^–/–^*;*Il1b^–/–^;Ntv-a* mice. (**A**) Schematic illustration of experimental design. (**B**) Kaplan-Meier survival curves of *Nf1*-silenced tumors generated in *WT;Ntv-a*, *Il1b^–/–^;Ntv-a*, *and* *Il1a^–/–^Il1b^–/–^;Ntv-a* mice. Survival curves were also created when mice were stratified by sex. Curves were compared by log-rank (Mantel-Cox) test; no significance was found. (**C**) UMAP dimensionality reduction of the scRNA-Seq data of tumors isolated from *WT;Ntv-a* (black, *n* = 3), *Il1b^–/–^;Ntv-a* (red, *n* = 3), and *Il1a^–/–^*;*Il1b^–/–^;Ntv-a* (blue, *n* = 3) mice. (**D**) UMAP of single cells in **C** colored by annotated broad cell classes. (**E**) *Il1a*, *Il1b*, and *Rfp* expression overlayed on the same UMAP coordinates. (**F**) UMAP dimensionality reduction of myeloid cells in the tumors subclustered and colored by annotated myeloid subtypes. (**G**) Dot plot of selected marker genes defining myeloid subtypes. (**H**) Composition of myeloid cell subpopulations in tumors generated in *WT;Ntv-a*, *Il1b^–/–^;Ntv-a* and *Il1a^–/–^*;*Il1b^–/–^;Ntv-a* mice. (**I** and **J**) tSNE plots of myeloid cells (**I**) and lymphocytes (**J**) and their quantification as examined by spectral flow cytometry for each genotype. *n* = 7, 6, and 6, respectively.

To determine whether the effects of IL-1β–independent MCP regulation in *Nf1* mGBM cells were also seen in vivo, we generated *Nf1* mGBM in *WT;Ntv-a*, *Il1b^–/–^;Ntv-a*, and *Il1a^–/–^;Il1b^–/–^;Ntv-a* mice (Figure 7A). Loss of Il1b or Il1 had no effect on the survival of tumor-bearing mice (Figure 7B). To determine the changes in *Nf1* mGBM, we performed scRNA-Seq from tumors generated in *WT;Ntv-a*, *Il1b^–/–^;Ntv-a*, and *Il1a^–/–^;Il1b^–/–^;Ntv-a* mice (Figure 7C and Supplemental Figure 14, A–H). Unsupervised clustering identified 5 major cell classes in these tumors, as observed for *PDGFB* tumors: lymphoid and myeloid immune cells, stromal cells, endothelial cells, and malignant tumor cells (Figure 7D), with myeloid cells accounting for most of the nonmalignant cells. Myeloid cells were again the major producers of *Il1a* and *Il1b*, while the *Rfp^+^*-transformed neoplastic cells had much lower expression in both *PDGFB* and *Nf1* mGBM (Figure 7E and Supplemental Figure 15). scRNA-Seq of PDGFB and *Nf1* mGBM revealed that *Nf1* mGBM cells are more prominent contributors to the total pool of MCP transcripts compared with PDGFB tumor cells (Supplemental Figure 16), further supporting the observation that *Nf1*-silenced mGBM cells express high levels of MCPs to recruit monocytes.

Next, we classified the myeloid cells into subsets (Figure 7, F and G), as done for *PDGFB* mGBM (Figure 4). In contrast to *PDGFB* mGBM, there was no reduction in the relative abundance of monocytes in *Il1b^–/–^;Ntv-a* or *Il1a^–/–^;Il1b^–/–^;Ntv-a* compared with *WT;Ntv-a* mice (Figure 7H), which was validated by FACS analysis (Figure 7, I and J, and Supplemental Figures 17 and 18). IHC demonstrated no changes in OLIG2, GFAP, or CD44 expression in tumors generated in *WT;Ntv-a*, *Il1b^–/–^;Ntv-a*, or*Il1a^–/–^;Il1b^–/–^;Ntv-a* DKO mice (Supplemental Figure 19). In addition, there were no changes in proliferation in tumors from the various genotypes, as assessed by pH3 staining (Supplemental Figure 19), in accordance with the mouse survival results (Figure 7B). Additionally, no changes in vessel size (CD31^+^ cells) were observed; however, there were reductions in IBA1- and P2RY12-positive areas (Supplemental Figure 20), without significant changes in the numbers of BMDMs or MG by FACS. This difference likely results from changes in cell size (defined as an immunopositive area) without concomitant changes in cell number. Although we did not observe a reduction in neutrophils by FACS (Supplemental Figure 17), we found reduced numbers of Elane^+^ neutrophils in tumors generated in *Il1a^–/–^;Il1b^–/–^;Ntv-a* DKO mice compared with WT (Supplemental Figure 20). These differences might be attributed to neutrophils being rare infiltrates that are extremely sensitive to dissociation techniques used for FACS analysis. These results are in line with previous studies on the role of IL-1β in neutrophil recruitment (34, 35).

Since we confirmed that loss of *Il1b* or *Il1* had no effect on the survival of mice with *Nf1*-silenced mGBM, we next sought to better define the role of *Il1* in *PDGFB* mGBM. First, we wanted to confirm the effect we observed with rIL-1B stimulation in *PDGFB*-driven primary cultures was not an in vitro cell culture–specific phenomenon, so we generated *PDGFB* mGBM in *WT;Ntv-a*, *Il1b^–/–^;Ntv-a*, and *Il1a^–/–^*;*Il1b^–/–^;Ntv-a* mice for quantification of CCL2, CCL7, CCL8, and CCL12 levels by ELISA. Loss of *Il1a/b* leads to decreased MCP production, which was more pronounced in tumors generated in *Il1a^–/–^*;*Il1b^–/–^;Ntv-a* mice compared with *Il1b^–/–^;Ntv-a* mice (Supplemental Figure 21). This reduction in MCP in *Il1b^–/–^* tumors is not apparent relative to *WT* mice, which might reflect that IL-1β–producing BMDMs are restricted to perinecrotic/perivascular areas of tumors and the ELISA was performed on whole-tumor lysates. Furthermore, because of functional redundancy, the effect on MCP reduction might be larger from the combined loss of both *Il1a* and *Il1b* in DKO mice. Finally, we observed no differences in VEGFA levels, which was used as an internal control (Supplemental Figure 21, *P* = 0.63). Taken together, these data establish that loss of IL-1 in the tumor and stroma leads to a reduction in MCPs in vivo, and this effect correlates with decreased infiltration of inflammatory monocytes into *PDGFB* mGBM.

### Germline Il1a and Il1b loss reverses the survival benefit conferred by Il1b deletion.

**Figure 8:**
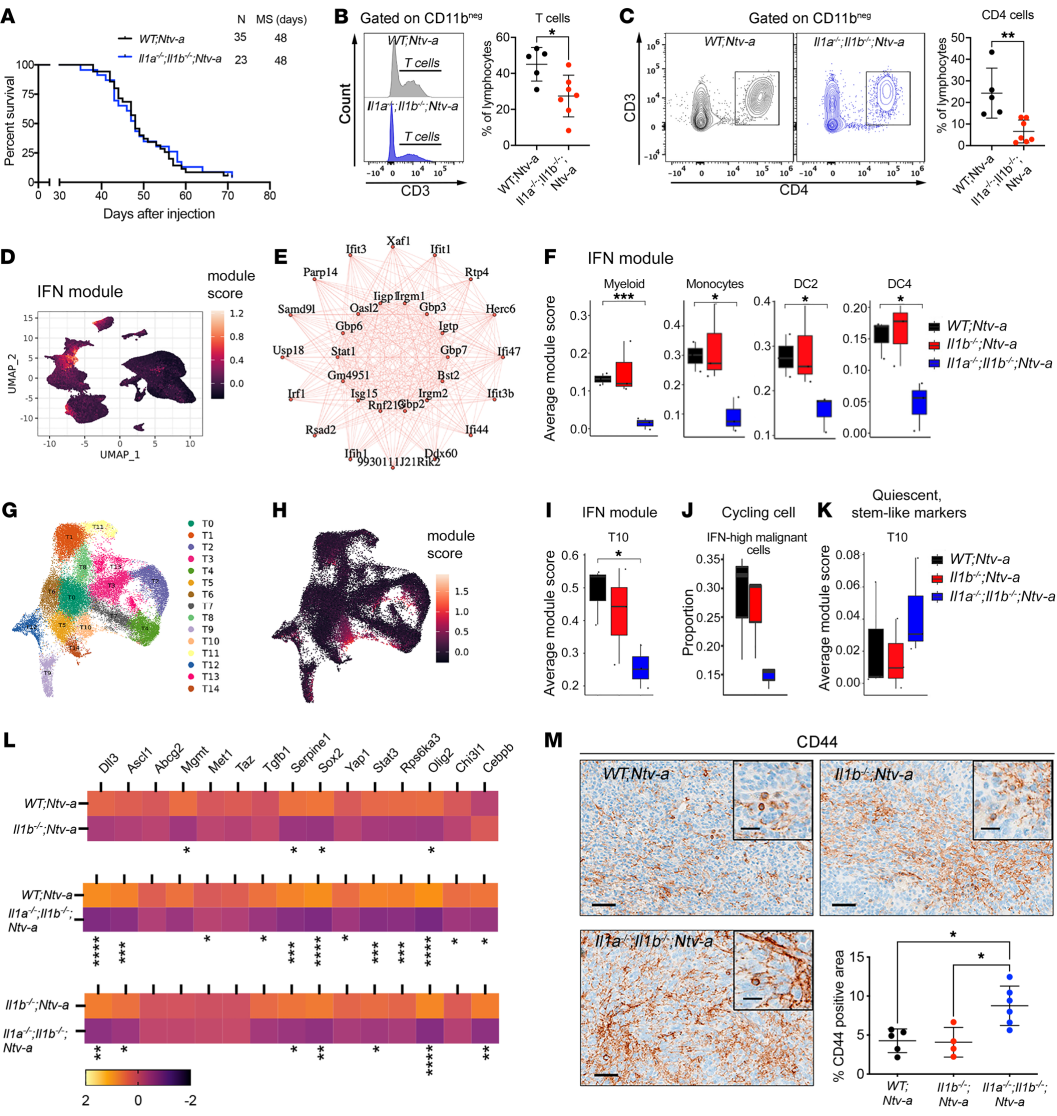
Genetic ablation of *Il1a/b* has no impact on the survival of *PDGFB*-driven GBM-bearing mice. (**A**) Kaplan-Meier survival curves of *PDGFB*-driven tumors generated in *WT;Ntv-a* and *Il1a^–/–^;Il1b^–/–^;Ntv-a* mice. (**B**) Histogram and quantification of CD3^+^ T cells examined by spectral flow cytometry. Two-tailed Student’s *t* test. *n* = 5 and 7, respectively. (**C**) Plots of CD4^+^ T cells and quantification examined by spectral flow cytometry. Two-tailed Student’s *t* test. (**D**) UMAP dimensionality reduction of all cells examined by scRNA-Seq and colored by expression of the IFN module derived by WGCNA. (**E**) Interconnected graph showing the top 30 most coexpressed genes in the IFN module. (**F**) Sample-averaged distributions of IFN module score in myeloid cell types grouped by genotype (*n* = 3 each group). Two-tailed Student’s *t* test. (**G**) UMAP dimensionality reduction of tumor cells, colored by assignment to 15 tumor clusters. (**H**) IFN module score overlayed on the same UMAP coordinates. (**I**) Sample-averaged distributions of IFN module score in cluster T10 grouped by genotype. (**J**) Per sample distributions of proportion of cycling cells in high IFN module score cells (IFN module score > 0) across malignant cells grouped by genotype. (**K**) Sample-averaged distributions of quiescent, stem-like module score in cluster T10 grouped by genotype. (**L**) qPCR analysis of expression of genes associated with stemness signatures in tumors generated in *WT;Ntv-a*, *Il1b^–/–^;Ntv-a*, and *Il1a^–/–^;Il1b^–/–^;Ntv-a* mice. *n* = 10 each group. Two-tailed Student’s *t* test. (**M**) IHC analysis of CD44 with quantification (*n* = 5, 4, and 6, respectively). One-way ANOVA with Tukey’s post-hoc test. Scale bars: 50 μm; 20 μm (insets). *P < 0.05; **P < 0.01; ***P < 0.001.

Considering the strong effect of combined *Il1a* and *Il1b* loss on MCP levels in tumors together with the strong effect on monocyte recruitment shown in scRNA-Seq data, we sought to determine whether genetic deletion of both *Il1a* and *Il1b* would further extend survival of tumor-bearing mice. Surprisingly, in contrast to the extended survival duration of *Il1b^–/–^;Ntv-a* mice (Figure 3A), the survival time of tumor-bearing *Il1a^–/–^*;*Il1b^–/–^;Ntv-a* mice was similar to that of the *WT;Ntv-a* mice (Figure 8A). Consistently, we did not observe any differences in the number of pH3-positive proliferating cells in the tumors between *WT;Ntv-a* and *Il1a^–/–^*;*Il1b^–/–^;Ntv-a* mice (Supplemental Figure 22).

To define the etiology for this seemingly paradoxical result, we analyzed immune cell content in these tumors by flow cytometry and found a reduction in the abundance of total myeloid cells, lymphoid cells, MG, and BMDMs in *Il1a^–/–^*;*Il1b^–/–^;Ntv-a* mice (Supplemental Figures 23 and 24, *P* < 0.05). This decreased MG abundance, which was not apparent in *Il1b^–/–^;Ntv-a* tumors (Figure 5C, *P* = 0.1667), might reflect decreased proliferation due to the loss of IL-1 signaling, which has been previously documented (36). To test whether this was the case, we compared the numbers of Ki67^+^ MG by FACS analysis and demonstrated that loss of *Il1a/b*, but not *Il1b*, leads to decreased MG proliferation (Supplemental Figure 23B). IHC staining for IBA1 demonstrated reduction of macrophages in *Il1b-* and *Il1a/b-*deficient tumors (Supplemental Figure 25). In contrast to *Il1b^–/–^;Ntv-a* mice, tumors generated in *Il1a^–/–^;Il1b^–/–^;Ntv-a* mice showed reduced CD3^+^ T cell infiltration (Figure 8B, *P* < 0.05), especially when considering CD4^+^ T helper cells (Figure 8C, *P* < 0.01). This reduction in both MG and CD4^+^ T cells may partially explain the survival differences seen between *Il1b^–/–^;Ntv-a* and *Il1a^–/–^;Il1b^–/–^;Ntv-a* mice.

To define the mechanism or mechanisms underlying the effects seen in *Il1a^–/–^;Il1b^–/–^;Ntv-a* mice, we performed weighted gene coexpression network analysis (WGCNA) adapted for scRNA-Seq data (37). This analysis identifies gene programs, or “modules” based on gene coexpression patterns (38). Interestingly, we found an IFN module (Figure 8D), where the 30 most coexpressed genes in this module are shown in an interconnected graph (Figure 8E), which shows a substantial decrease in IFN module activity in DKO mice across many cell types, including total myeloid cells (*P* < 0.001), monocytes (*P* < 0.05), DC2s (*P* < 0.05), and DC4s (*P* < 0.05, Figure 8F). Similarly, unsupervised clustering on malignant cells identified 15 subclusters (T0–T14 in Figure 8G and Supplemental Figure 26), where cells in cluster 10 (T10) exhibited a decreased IFN signaling response specifically in *Il1a^–/–^;Il1b^–/–^;Ntv-a* mice (Figure 8, H and I). When we examined the proliferation properties of these cells, we found a sharp decrease in the proportion of cycling cells as a fraction of all malignant IFN high cells in *Il1a^–/–^;Il1b^–/–^;Ntv-a* mice (Figure 8J), suggesting they may be in a quiescent state. This observation inspired us to speculate that these cells could be more stem-like cells entering quiescence in response to an absence of IFN stimulation from the myeloid cells. Consistent with this idea, scRNA-Seq analysis revealed an upregulation of markers associated with quiescent stem-like GBM cells in cluster T10 (Figure 8K), although this finding will require further mechanistic exploration to determine cause and effect (39).

Using a previously established qPCR panel to examine the molecular profiles of stem-like cells in mGBM (15), we analyzed tumors generated in *WT;Ntv-a*, *Il1b^–/–^;Ntv-a* and *Il1a^–/–^;Il1b^–/–^;Ntv-a* mice. Although some of these genes remained unchanged among the 3 genotypes, we observed an IL-1 dose-dependent reduction (stronger effects when both *Il1a* and *Il1b* are lost relative to *Il1b* loss alone) in *Sox2*, *Olig2*, and *Serpine1* expression (40) (Figure 8L). In contrast to *Il1b*-null tumors, tumors from combined *Il1a/b* loss mice showed a reduction in *Ascl1* expression, important for the establishment of neuronal fate and loss of self-renewal (41). Loss of *ASCL1* also inversely correlates with the expression of CD44, another prominent stem cell marker (42), which we confirmed by IHC (Figure 8M, *P* < 0.05). Taken together, these findings indicate GBM progression is controlled by both IL-1α and IL-1β. To demonstrate that IL-1α antagonizes the tumor-promoting effects of IL-1β, we generated tumors in *WT;Ntv-a* and *Il1a^–/–^;Ntv-a:* loss of *Il1a* resulted in accelerated tumor growth, which was the opposite of our observation in *Il1b*-deficient mice (Supplemental Figure 27).

### Local antagonism of IL-1β or IL-1R1 prolongs the survival of tumor-bearing mice.

**Figure 9:**
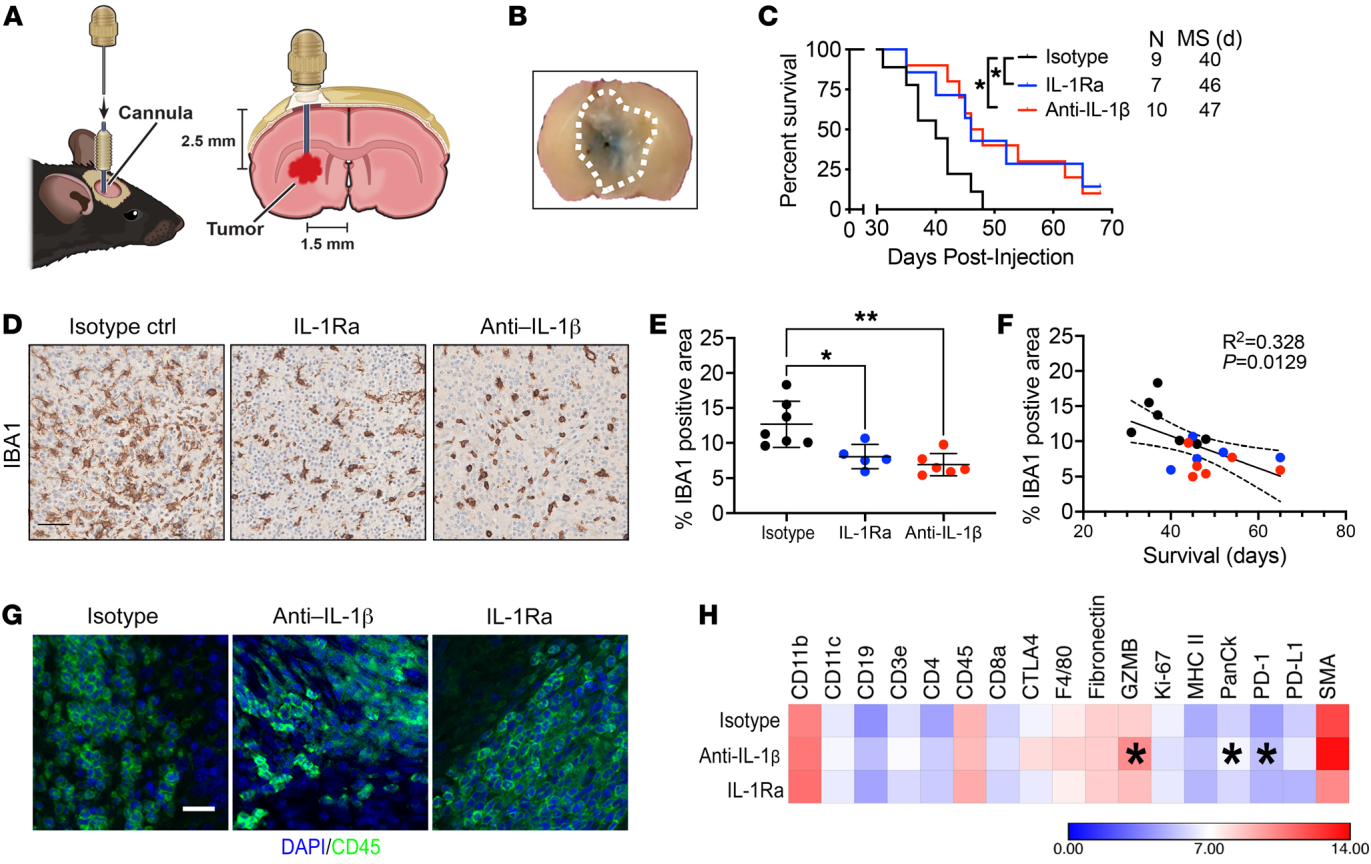
Intratumoral anti–IL-1β antibody or IL-1Ra administration prolongs survival of GBM-bearing mice. (**A**) Illustration of experimental design. (**B**) Trypan blue dye used to visualize drug delivery efficiency. Dotted line outlines tumor. (**C**) Kaplan-Meier survival curves of *PDGFB*-driven tumor-bearing *WT;Ntv-a* mice following treatment with vehicle anti–IL-1β antibody or IL1R antagonist. Mantel-Cox test. *n* = number of mice. (**D**) Representative IBA1 IHC images of tumors from vehicle-, anti–IL-1β antibody, or IL1R antagonist-treated tumor-bearing mice. (**E**) Quantification of IBA1-positive tumoral areas. One-way ANOVA, Tukey’s multiple comparison test. *n* = 7, 5, and 6, respectively. (**F**) IBA1 expression correlates with survival of tumor-bearing mice. Colors of data points match the genotypes depicted in **E**. (**G**) Photomicrographs of tumors stained≈with DAPI (blue) and CD45 (green) to guide ROI selection for NanoString GeoMx multiplexed protein quantification. Scale bars: 25 μm. (**H**) Quantification of protein expression by GeoMx assay. Heatmap color intensity indicates log_2_ expression. Student’s *t* test compared with isotype controls. *n* = 4 mice per group.*P < 0.05; **P < 0.01.

Since all of the genetic experiments were performed in germline *Il1a/b-* or *Il1b*-deficient mice, we sought to determine whether locally neutralizing IL-1β or introducing an IL-1R1 antagonist (IL-1Ra) would impede tumor growth and prolong the survival of tumor-bearing mice without interfering with normal development and function of immune cells. We created *PDGFB*-driven GBM in *WT;Ntv-a* mice (or *WT;Ntv-a;Cdkn2a^–/–^* mice and observed similar results between these 2 strains) and allowed tumors to grow for 15 days. We then installed guide cannulas into the brains of each mouse through the same burr hole used for tumor initiation (Figure 9A). These cannulas enabled us to repeatedly deliver temperature-sensitive anti–IL-1β antibodies or IL-1Ra daily without damaging brain tissue. This direct delivery system also avoided the caveat of low bioavailability when the compounds are injected via the intraperitoneal route. We divided the mice into 3 groups, receiving either vehicle control containing isotype-control antibodies (1 mg/day/mouse), IL-1Ra (500 ng/day/mouse), or anti–IL-1β antibody (1 mg/day/mouse) daily. To confirm the efficacy of this delivery system, we injected trypan blue (0.25% in PBS) as an indicator via the cannula 24 hours prior to euthanasia in a small cohort of mice. We found the blue dye diffused in the tumor, covering about half of the tumor mass (Figure 9A). Kaplan-Meier survival curves demonstrated that pharmacologically antagonizing IL-1β or IL-1R1 prolonged the survival of GBM-bearing mice (median survival = 47 and 46 days, respectively) relative to vehicle controls (median survival = 40 days) (Figure 9B, *P* < 0.05). Interestingly, when we examined the tumor tissues of these mice with IHC (Figure 9C), we found a reduction in IBA1-positive cell density, suggesting a reduction in TAM infiltration (Figure 9D, *P* < 0.05). In fact, reduced TAM content in the tumor serves as an indicator of improved prognosis (Figure 9, E and F). To further evaluate the changes due to the treatments, we used NanoString GeoMx multiplexed 17 immune protein panel staining (Figure 9, G and H). Even though the region of interest (ROI) selection was random and did not cover the entire tumor, it should be appreciated that direct delivery of anti–IL-1b antibodies and IL-1Ra into the tumors resulted in only local inhibition due to limited antibody diffusion. As shown in Figure 9H, we observed increased granzyme B (GZMB) and decreased PD-1 expression. A similar elevation in GZMB levels was demonstrated in a breast cancer transplant model in *Il1b*-deficient mice, where a combination of anti–IL-1β synergized with anti–PD-1 antibodies to restore the function of anergized antitumor T cells and resulted in complete inhibition of tumor growth (43). In line with these results, a recent study showed that GBM cells in the hypoxic niche induce IL-1β in TAMs, promoting the trafficking/sequestration of TAMs and cytotoxic T cells, which are reprogrammed into an immunosuppressive state (22). To determine the significance of extended survival in those de novo primary GBM-bearing mice with IL-1 targeting, we used a clinically relevant TMZ dose of 25 mg/kg administered daily by oral gavage for 2 weeks, which provided a significant survival advantage compared with vehicle-treated animals (8.5 days), which was comparable to anti–IL-1b local targeting (Supplemental Figure 28, A and B). Together, these results establish a rationale for clinical translation of local administration of IL1-targeted therapy for PN GBM.

## Discussion

In this study, we analyzed the mechanistic role of IL-1 signaling in driving TAM tumor cell crosstalk that promotes tumor progression. Using both genetic and pharmacological tools, we demonstrated local suppression of IL-1β/IL-1R1 signaling can markedly prolong the survival of GBM-bearing mice. Previous studies investigating the role of IL-1β signaling in in vitro GBM patient-derived primary cell cultures have yielded inconsistent results: some demonstrate no effects on tumor cell proliferation, while others either demonstrate tumoricidal properties of rIL-1β or tumor-promoting properties, all depending on cell culture conditions and time of exposure to rIL-1β (29, 44–49). Thus, any in vitro investigations may not reliably recapitulate the complexity of IL-1β function in pathophysiological conditions in vivo.

We set out to determine whether tumor genotype and/or timing of IL-1β inhibition (germline loss versus local tumor targeting) affect GBM growth in vivo by using GEMMs of de novo primary GBM. Our results demonstrate both RNA and protein levels of *IL1B* are increased in *IDH*-WT GBM patient samples and that this elevated expression inversely correlates with patient survival. Genetic deletion of *Il1b*, or locally suppressing IL-1 signaling within tumors, can markedly prolong survival of GBM-bearing mice. Our results provide a rationale for using IL-1β–targeted therapy as a practical and effective treatment for PN GBM.

We observed a significantly higher level of IL-1β in murine and human MES GBM compared with PN GBM at both the RNA and protein levels (Figure 2). This is not surprising, as it has been established MES GBM are more immunoactive than PN GBM (3). The observation we made in this study is that MES GBM cells have constitutive NF-κB pathway activation. This observation agrees with a previous study demonstrating active NF-κB signaling in human MES, but not PN, GSC cultures (33). These results clearly illustrate the genotype-specific effect of IL-1β and can partially explain discrepancies in the literature where tumor cell genotype was not considered as a covariate. It appears that both in vitro and in vivo, MES cells may have reached a plateau in NF-κB activity, so that any exogenous IL-1β supplemented to the cell culture did not further increase NF-κB phosphorylation (Figure 6). *Nf1* mGBM have constitutively active NF-κB, similar to what was shown for human GBM lines with *Nf1* loss (MES signature) (33). Since the loss of *Nf1* leads to Ras pathway activation and Ras signaling can activate the NF-κB pathway (50, 51), it is not surprising to observe a constitutively active NF-κB in *Nf1* mGBM primary cultures, and this effect does not depend on IL-1R1. In addition, it has been documented that IL-1β expression can be regulated by NF-κB (52), which can explain the increased levels of IL-1β in *Nf1* mGBM and primary cultures.

Decreased inflammatory monocyte recruitment in *Il1b*-deficient mice was similar to what we observed previously in *Il1R1*-deficient mice (8), which we hypothesized was due to diminished MCP production and thereby reduced monocyte recruitment via chemotaxis. The effect of *Il1* ablation on the MCP network in vivo was more pronounced when both *Il1* ligands were ablated. Interestingly, in tumors lacking the *Il1b* gene, there was a significant induction of chemokine CCL7 (Supplemental Figure 21), in contrast to the in vitro data performed with tumor cell cultures (Supplemental Figure 12, C and D). This increase in CCL7 in vivo could possibly be due to its increased production by other cell types in the TME, similar to what was shown in a lung cancer model, where loss of CCL7 accelerated tumor growth (53).

We found the loss of *Il1b* prolongs survival while the loss of *Il1a* shortens survival of tumor-bearing mice. Ablation of both *Il1a* and *Il1b* in vivo resulted in more potent decreases in both monocyte recruitment and total TAMs; however, the survival duration did not differ from that of *WT;Ntv-a* mice. Our scRNA-Seq data revealed a marked disruption of the IFN signaling pathway in both the myeloid compartment and malignant cells in *Il1a^–/–^;Il1b^–/–^;Ntv-a* DKO mice. Although playing divergent roles in host defense, it is known that IL-1 and IFN pathways can crosstalk with each other in various diseases (54). Type I IFN can actively regulate IL-1 expression and may also antagonize its biological functions; however, much less is known about how IL-1 could in turn regulate IFN production and/or its effector functions (54). When IFN signaling is altered, tumors in *Il1a^–/–^;Il1b^–/–^;Ntv-a* mice demonstrated increased stemness features and decreased proliferation (Figure 8). Based on this observation, we conclude complete ablation of *Il1* results in the impairment of IFN signaling in GBM, allowing tumor immune escape and resulting in reduced mouse survival.

Pharmacological antagonizing *Il1* signaling locally in tumor-bearing mice extended survival time and decreased TAM infiltration, which was opposite of what was observed with *Il1a^–/–^;Il1b^–/–^;Ntv-a* mice. This observation suggests the differences seen in *Il1*-deleted mice can be attributed to germline loss of *Il1* that leads to decreased lymphoid infiltration and IFN loss, although in these mice, TAM infiltration is also diminished. Therefore, reduction in tumor-promoting TAMs alone cannot offset the combined detrimental effects of lymphoid and IFN loss in *Il1a^–/–^;Il1b^–/–^;Ntv-a* mice. On the contrary, local delivery of IL-1–targeted therapy in immunocompetent mice reduced TAM infiltration but did not alter IFN signaling, thereby providing effective inhibition of tumor growth. Our results also indicate IL-1α and IL-1β may perform distinct functions in GBM. Unlike IL-1β, which functions exclusively through binding IL-1R1 on the plasma membrane after proteolytic cleavage and inflammasome formation in myeloid cells, IL-1α can be produced by nearly all cells and be released upon cell death where it can then translocate into the nucleus and bind to transcription factors and activate expression of proinflammatory cytokines and chemokines independently of IL-1R1 (55, 56).

A recent study showed a dichotomous distribution of these 2 suppressor cell types in different sexes of GBM-bearing mice when the tumors were generated by transplanting GL261 tumors isolated from a male donor (20). Here, however, we did not observe any differences in mMDSC and gMDSC infiltration when we stratified our data by sex (data not shown). Additionally, to evaluate whether the sex effect observed in the previous publication was a donor-sex–specific result, we performed experiments where male and female donor-derived primary tumor cells were transplanted into male and female recipients, as illustrated in Supplemental Figure 29. We did not observe sex-specific differences in their myeloid composition or the survival times of tumor-bearing mice (Supplemental Figure 29, B and C). It is possible that the differences between our current data and the prior report are model specific and therefore warrant further investigation.

In summary, our findings demonstrate a protumorigenic function of IL-1β in PDGFB-driven mGBM and show that blocking IL-1β signaling decreases inflammatory monocyte recruitment, MG abundance, and the frequency of exhausted CD8^+^ T cells, prolonging survival of tumor-bearing mice. Along with our previous findings showing targeting IL-1β can effectively reduce cerebral edema in GBM models (8), our results here further support the application of antagonism of IL-1β as a promising therapy for PN GBM. These studies provide strong rationales for clinical translation of antagonizing IL-1β in treating GBM.

## Methods

Detailed methods can be found in Supplemental Methods.

### Mice.

Animals were housed in a climate-controlled, pathogen-free facility with access to food and water ad libitum under a 12-hour light/12-hour dark cycle. Tumor-bearing mice were euthanized at humane end points to ensure comparable tumor burden across all animals. Specific details regarding the mice used can be found in the Supplemental Methods.

### Virus generation and tumor induction.

We delivered RCAS-*PDGFB* in *Cdkn2a^–/–;^Ntv-a* mice or the combination RCAS-*PDGFB* with RCAS-shRNA-*p53-Rfp* in *Ntv-a* mice (15, 16). To generate a murine MES model, we chose to silence the tumor suppressor genes NF1, TP53, and PTEN by coinjection of RCAS virus carrying shRNA of these molecular targets, along with introducing PDGFA (15). Cells were transfected with RCAS viruses using a Fugene 6 Transfection Kit (Roche, 11814443001) according to the manufacturer’s instructions. DF-1 cells (4 × 10^4^) were stereotactically delivered in the right frontal-striatum for tumor generation (15, 16).

### Orthotopic glioma generation.

The same procedure was used as described above, except 3 × 10^4^ of freshly dissociated tumor cells were injected in the right frontal-striatum.

### MG and MDM isolation and culture.

MG were isolated from P0 to P3 pups and BMDMs were isolated from femur and tibia of adult C57BL6/J mice using a modification of previously described protocols (8, 57). Details are provided in Supplemental Methods.

### Organotypic tumor slice culture.

Brains of tumor-bearing mice were rapidly extracted and embedded in 4% low-melt agarose in PBS. The embedded brain was then mounted on a Vibratome (Leica, 1220S) and cut into 300 μm thick sections. Slices were cultured in Neurobasal medium (Stem Cell Technologies, 05700) supplemented with B27 (Thermo Fisher, 17504044), sodium pyruvate (Thermo Fisher, 11360070), and glutamine (Thermo Fisher, 35050061). M-CSF (BioLegend, 576406) was included during coculture experiments with BMDM.

### Tumor dissociation and primary cell culturing.

Tumor dissociation and culture of primary tumor cells was performed as previously described (30).

### IL-1β treatment in vitro.

For primary neurospheres, cultures (before rIL-1β stimulation) were dissociated with Accutase (Sigma-Aldrich, A6964) to generate single cells. Cells were stimulated with 100 pM rIL-1β (R&D 401-ML/CF) for periods indicated in the graphs.

### MES tumor cell cultures and in vitro stimulation.

Murine MES GBM lines (lines 1816 and 4622) were cultured as either neurospheres or FBS cultures as previously described (31, 32, 58). For treatment with NF-κB inhibitors, the cells were subcultured at 10^5^ cells/well in a 24-well plate precoated with Geltrex (Life Technologies, A14132-01). Supernatant was collected from each well of the cultured GBM cells and transferred to BMDM or MG cultures for stimulation.

### Tumor and cultured cell RNA isolation and qPCR analysis.

Mice were sacrificed at humane end points. A piece of tumor was immediately snap-frozen in liquid nitrogen for storage at –80°C. Alternatively, cultured cells were harvested from plates using TRIzol (Thermo Fisher, 15596026). RNA was isolated from the frozen tumor pieces or cells with the RNeasy Lipid Tissue Mini Kit (QIAGEN, 74804). qPCR was performed with the validated Bio-Rad PCR primers using SsoAdvanced Universal Green Supermix (Bio-Rad, 1725271, detailed in Supplemental Table 1). β-Actin or HPRT was used as a housekeeping gene.

### Immunoblot analysis.

Mouse primary GBM cell lines were treated under the following conditions: (a) 100 pM IL-1β (R&D Systems, 401-ML/CF) for 10, 30, and 60 minutes; (b) 10 and 20 μM BAY 11-7082 (MilliporeSigma, 196870) for 24 and 48 hours; and (c) 10 and 20 μM cardamonin (Tocaris, 2509) for 24 and 48 hours. Protein from cell lysates was supplemented with a complete protease inhibitor cocktail (Roche) and subjected to SDS–polyacrylamide gel electrophoresis after determining protein concentration with a BCA kit (Pierce, 23227). Membranes were incubated with primary antibody as described in Supplemental Table 2. Immunodetection was performed with Chemiluminescent HRP Antibody Detection Reagent (Denville Scientific Inc., E2400). Details are provided in Supplemental Methods.

### Human tissue samples and pathological appraisal.

Archived formalin-fixed, paraffin-embedded (FFPE) human GBM samples and deidentified clinical information were provided by Emory University, and patient information is included in our published manuscript (3). Fresh tumor tissues used for ELISA quantification were collected at Mount Sinai Hospital through the biorepository. Specimen meta-information is summarized in Supplemental Table 3. Board-certified neuropathologists diagnosed and graded both human and murine tumor tissue according to the 2016 WHO Classification of Tumors of the Central Nervous System (17).

### TCGA analysis.

U133 microarray data for the GBM (TCGA, provisional) data set were downloaded from cBioPortal (https://www.cbioportal.org) in August 2021 and sorted into subtypes based upon a proprietary key. G-CIMP–positive tumors were excluded from analysis. Cox’s proportional hazard models were fitted in R using age and gene expression as continuous covariates and sex as a binary variable. Forest plots were done using the function *ggforest*.

### Tissue processing and IHC.

Archived FFPE human GBM samples were sectioned at 5 μm thickness, slide mounted, and stored at –80°C. For H&E tumor validation and IHC staining, brains were fixed in 10% neutral buffered formalin for 72 hours at room temperature, processed in a tissue processor (Leica, TP1050), embedded in paraffin, sectioned, and slide mounted.

IHC staining was performed on either the Discovery XT platform (Ventana Medical Systems) or Leica Bond Rx (Leica). Primary antibodies used in this study are described in Supplemental Table 2. Image analysis was performed using Fiji (NIH).

### Immunofluorescence.

Human GBM 5 μm FFPE sections were stained with anti-IBA1 (1:500, Wako, 019-19741) and anti–IL-1β (NCI preclinical repository, biological resource branch, 32D). Secondary antibodies conjugated to Alexa Fluor dyes (555 nm, 647 nm from Invitrogen) were applied. DAPI (MilliporeSigma, D9542) was used for nuclear counterstaining. Fluorescence images were taken on an Olympus FV1000 confocal microscope and analyzed with FIJI (NIH). Details are provided in Supplemental Methods.

### ELISA.

Cell lysates for ELISA were collected via sonication of cells in lysis buffer supplemented with protease and phosphatase inhibitors. Protein concentrations were determined using a Bradford Protein Assay (Bio-Rad, 5000001). ELISAs were performed on cell lysates and supernatants.

### Flow cytometry and spectral flow cytometry.

Single tumor cell suspensions were generated as previously described. Cells were stained with primary antibodies (Supplemental Table 2). Cells were washed and fixed with fixation buffer (eBioscience, 00-5123-43, 00-5223-56, or 00-8333-56) before being stained with the cocktail of antibodies for intracellular markers. All data were collected on a BD LSR II flow cytometer or Cytek Aurora spectral flow cytometer. Data were analyzed using FlowJo, version 10, software (BD Bioscience) based on our published protocols (30).

### scRNA-Seq and data analysis.

Single tumor cell suspensions were obtained by papain dissociation as described above. scRNA-Seq was performed on these samples using the Chromium platform (10x Genomics) with the 3′ Gene Expression (3′ GEX) V3 kit, using an input of approximately 10,000 cells. The targeted depth was at 50,000–100,000 reads per cell.

Raw fastq files were aligned to mouse genome reference mm10, customized to include the Rfp sequence, using CellRanger, version 5.0.0 (10x Genomics). Count matrices were filtered and assessed for quality control before being processed and analyzed using R package Seurat, version 4.0.5. Normalization was performed using the NormalizeData function. Dimensionality reduction was computed using the FindVariableFeatures, ScaleData, and RunPCA functions. De novo clustering using the Louvain algorithm was applied. Cell-level proliferation analysis was carried out with CellCycleScoring.

Identification of modules of coexpressed genes was carried out using the R package scWGCNA (https://github.com/smorabit/hdWGCNA/tree/dd63fa9de19d548a9c82c78742f27ebbef4b27dc; commit ID: dd63fa9). To identify modules, function blockwiseConsensusModules was called. Only the top 2,000 variable genes were used.

### H&E tumor volume reconstruction.

Tumor brain was extracted, fixed, embedded in OCT compound (VWR, 25608-930), and frozen on dry ice. The entire brain was serially sectioned on a cryostat (Leica) to 30 μm sections and tumor volume was reconstituted based on our published protocol (8). Details are provided in Supplemental Methods.

### Cannula installation and drug administration in tumor-bearing mice.

Mice were anesthetized and placed into a stereotactic device. The burr hole used to inject the RCAS virus was reprobed with a compact drill. A guide cannula (Plastics One) was implanted into the brain through the craniotomy and fixed in place with dental acrylic. A matching stylet or “dummy-cannula” was then screwed onto the guide cannula to prevent back flowing of the CSF or environmental debris from entering the guide cannula.

Mice were anesthetized with isoflurane. Up to 2 μl of isotype antibody, (BE0091, BioXcell), IL-1RA (US Biological Corporation, I7663-62E), or purified anti–IL-1β IgG (BioXcell, BE0246) was injected into the lateral ventricle over 1 minute.

### In situ proteomics by NanoString GeoMx.

FFPE sections of 5 μm thickness were placed onto positively charged slides (Thermo Fisher). The GeoMx Immune Cell Profiling Panel Mouse Protein Core with a 20-gene selection was used for nCounter readout along with morphology markers pan-cytokeratin (Alexa Fluor 488), CD45 (Alexa Fluor 647), and DAPI (nuclei) staining. Six circular ROIs 300 μm in diameter were utilized for targeted spatial transcriptomics using the GeoMx platform (NanoString). ROIs were generated in the tumor-rich region of the tissue sections. Once digital spatial profiling data was completed, the samples were pooled for nCounter readout in RCC format.

Data were subsequently normalized against GAPDH and Histone H3, 2 housekeeping genes. The normalized data were used for comparisons between treatment groups and vehicle controls and to generate corresponding heatmaps.

### Statistics.

Graphs were created using GraphPad Prism 8 (GraphPad Software Inc.) or R. Variables from 2 experimental groups were analyzed using unpaired or paired parametric Student’s 2-tailed *t* tests as appropriate, assuming equal SDs. One-way ANOVA was used to compare variables from more than 2 groups. Kaplan-Meier survival analysis was used to analyze survival differences using the log-rank (Mantel-Cox) test and the Gehan-Breslow-Wilcoxon test. Data are represented as mean ± SD. Numbers of samples in each group are indicated in the individual figures.

### Study approval.

All experimental procedures were approved by the IACUC of Emory University (protocol 201700633) and the Icahn School of Medicine at Mount Sinai (protocol 201900619). Archived FFPE human GBM samples and deidentified clinical information were provided by Emory University (IRB study 18-177), and patient information is included in our published manuscript (3). Fresh tumor tissues used for ELISA quantification were collected at Mount Sinai Hospital through the biorepository under an IRB-approved protocol (18-00983). All patient samples were deidentified, and specimen meta-information is summarized in Supplemental Table 3.

### Data availability.

The data that support the findings of this study are available from the corresponding author upon request. scRNA-Seq data were deposited in the NCBI’s Gene Expression Omnibus database (GEO GSE200348). Values for all data points in graphs are reported in the Supporting Data Values file.

## Author contributions

DH, ZC, and AMT conceived the project. ZC, DH, AMT, and BG developed the methodologies for the study. ZC, MK, CJH, G Pinero, MPV, JLR, JA, VM, FS, MMR, AA, NN, G Price, KR, and WT performed experiments. ZC, BG, and SC prepared the figures. ZC, BG, MK, SC, KR, FM, AMT, and G Price analyzed the data. NMT, DHG, and DMS provided resources. Software: ZC, SC, BG, and AMT. ZC and DH wrote the original draft of the manuscript. SC, DHG, BG, and AMT reviewed and edited the manuscript. DH acquired funding. DH and AMT supervised the project.

## Supplementary Material

Supplemental data

Supporting data values

## Version 1 09/21/2023

In-Press Preview

## Version 2 11/15/2023

Electronic publication
