# Supplementary material for: A paracrine circuit of IL-1β/IL-1R1 between myeloid and tumor cells drives genotype-dependent glioblastoma progression
Source: J Clin Invest. 2023 Nov 15;133(22):e163802. doi: 10.1172/JCI163802 (PMC10645395; doi:10.1172/JCI163802)
Supplement: Supplemental data [file jci-133-163802-s206.pdf]

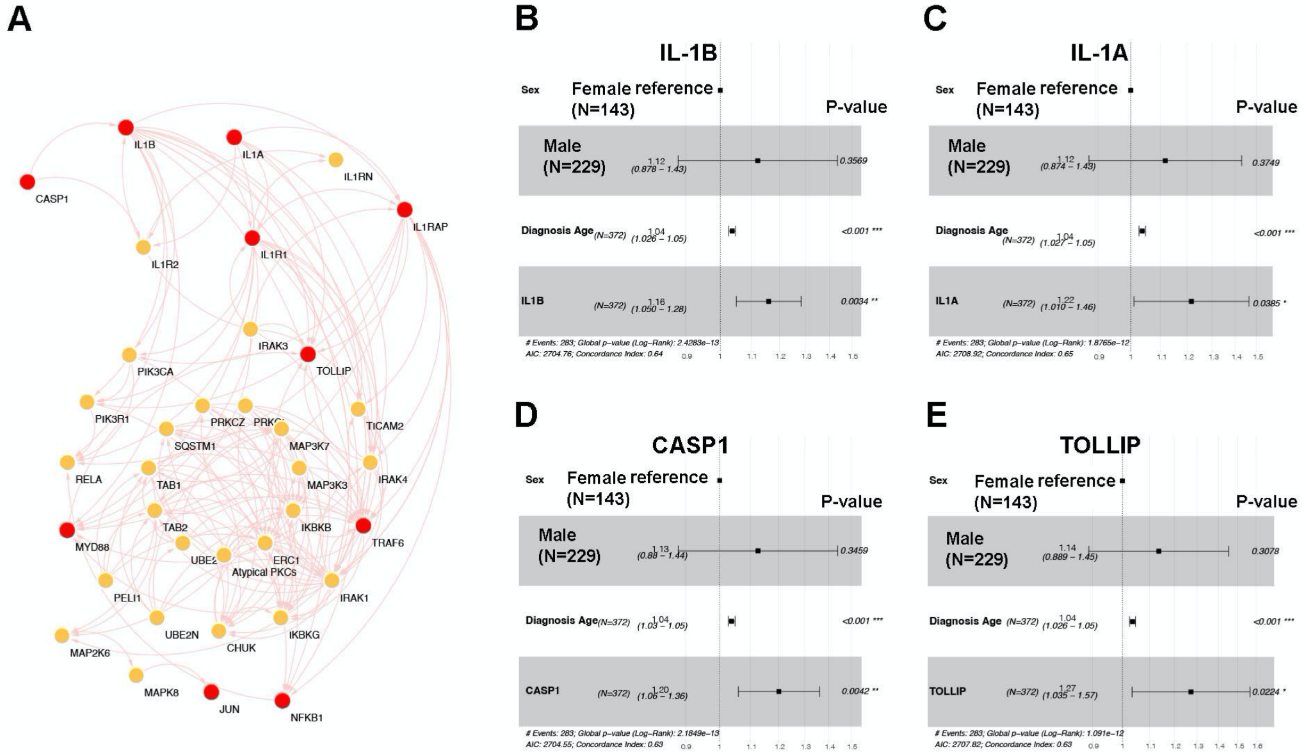

Supplementary Figure 1. Association between the expression of *IL-1* pathway and the survival time of IDH-WT GBM patients. **(A)** Molecules of the *IL-1* signaling pathway. Red dots highlight selected molecules that are prominent players in this pathway. **(B)** – **(E)** Forrest plots generated using Cox Proportional Hazards models, using expression of different genes (indicated) as continuous covariates. Hazard ratio (HR) is shown on X-axis; P-values are indicated on the right. N as indicated.

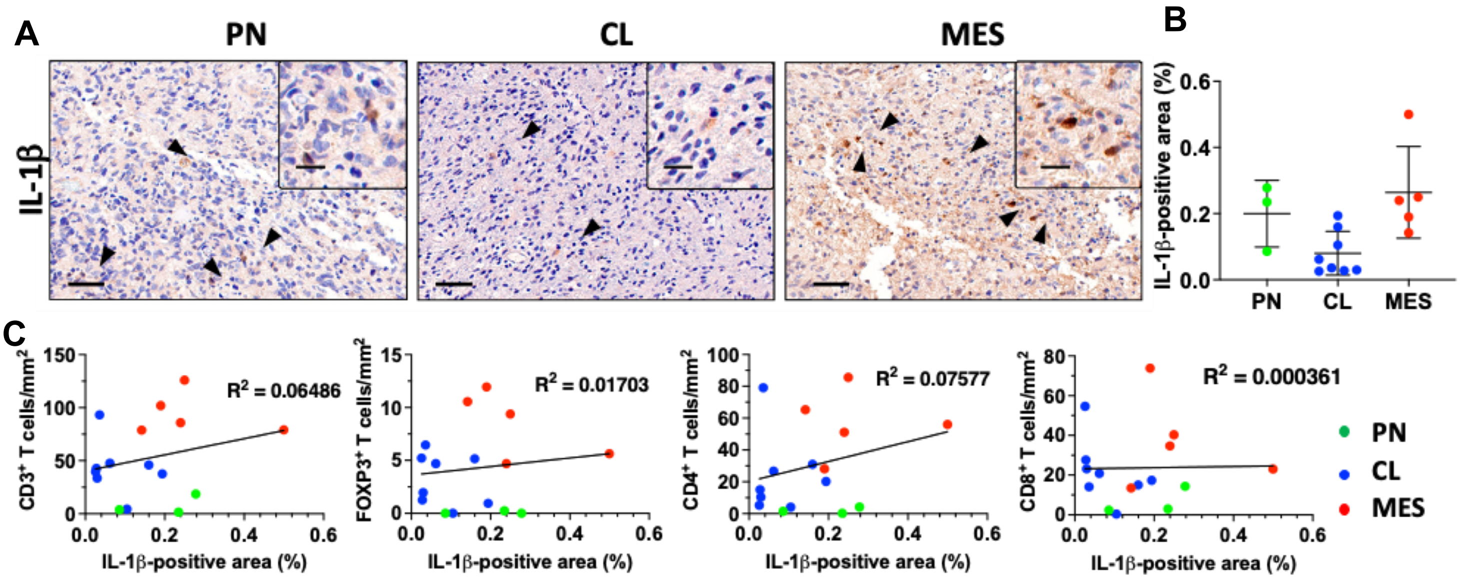

Supplementary Figure 2. Immunohistochemical staining of IL-1β in human GBM tissue sections. **(A)** Representative micrograph of IL-1b staining. Arrows indicates immuno-positivity. **(B)** Quantification of positive areas of IL-1b. **(C)** Correlations between IL-1β staining and T cell subsets. PN = Proneural, N=3; CL = Classical, N=8; MES = Mesenchymal, N=5. Scale bar = 50 um for main graphs and 20 um for insets.

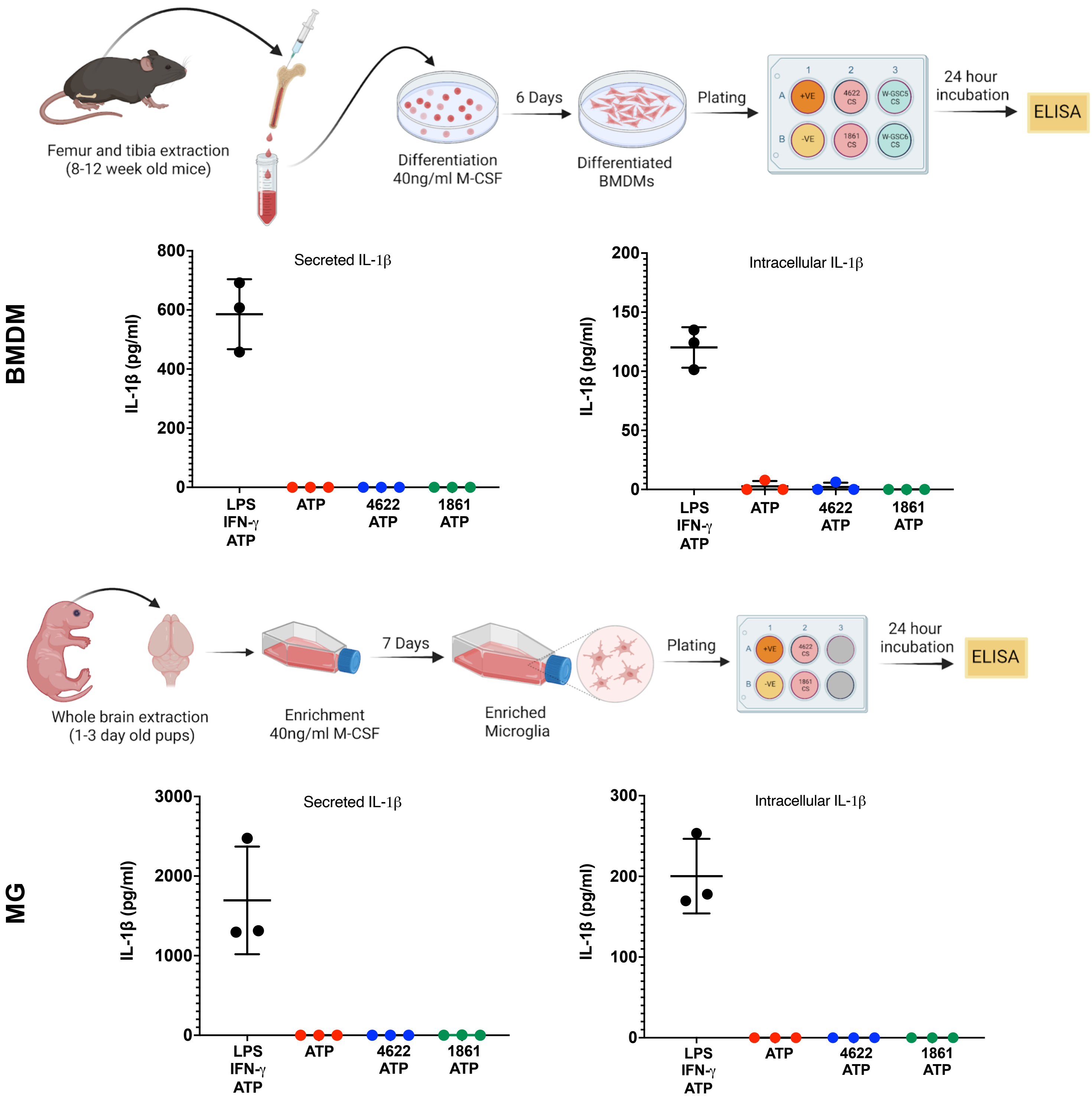

Supplementary Figure 3. *Nf1* mGBM cells do not induce IL-1β production by either BMDM (top) or MG (bottom) cells as examined by ELISA. LPS+IFNγ+ATP was used as a positive control and ATP alone as negative control. N=3 each group.

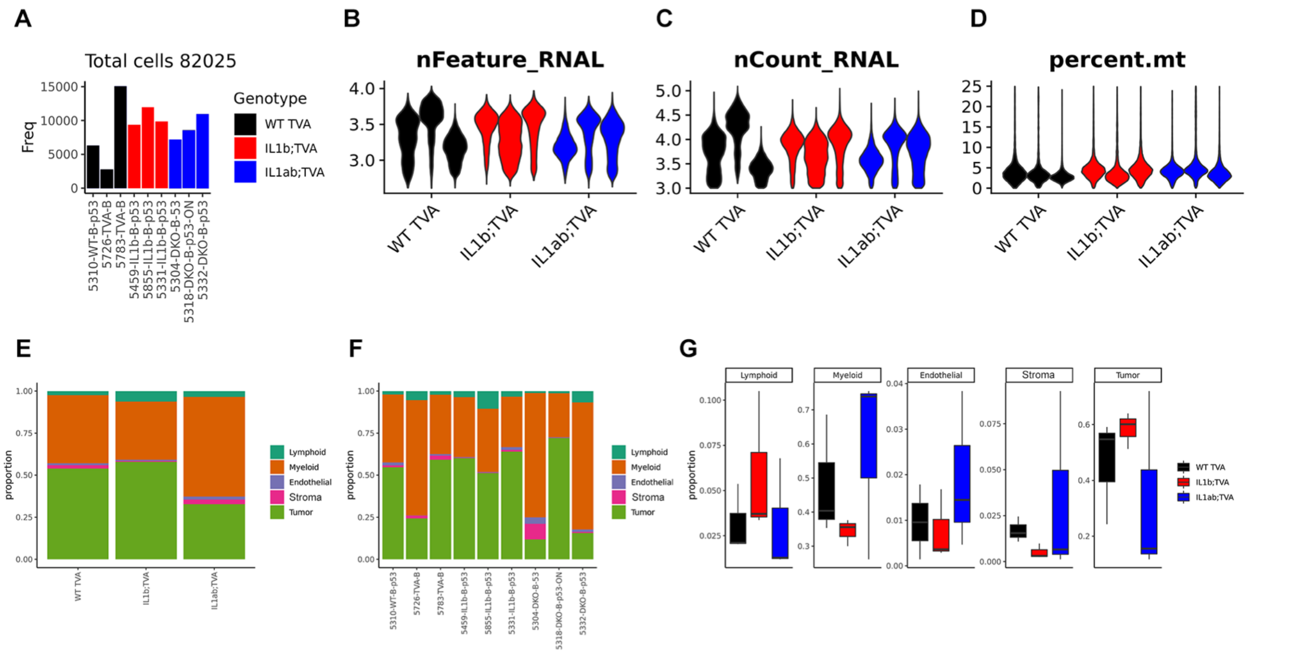

Supplementary Figure 4. ScRNA-seq analysis of PDGFB mGBM generated in *WT;Ntv-a*, *Il1b$^{-/-}$;Ntv-a* and *Il1a$^{-/-}$;Il1b$^{-/-}$;Ntv-a* mice. (A) Total number of cells per samples after removing doublets. **(B)** Distribution of number of unique molecular identifier (UMI) per cells per sample. **(C)** Distribution of number of genes detected per cell per sample. **(D)** Distribution of percentage of mitochondrial genes per cell per sample. **(F)** Proportion of the five major cell classes grouped by individual samples. **(G)** Boxplot showing the distribution of the five major cell classes in the three genotypes. N=3 each group.

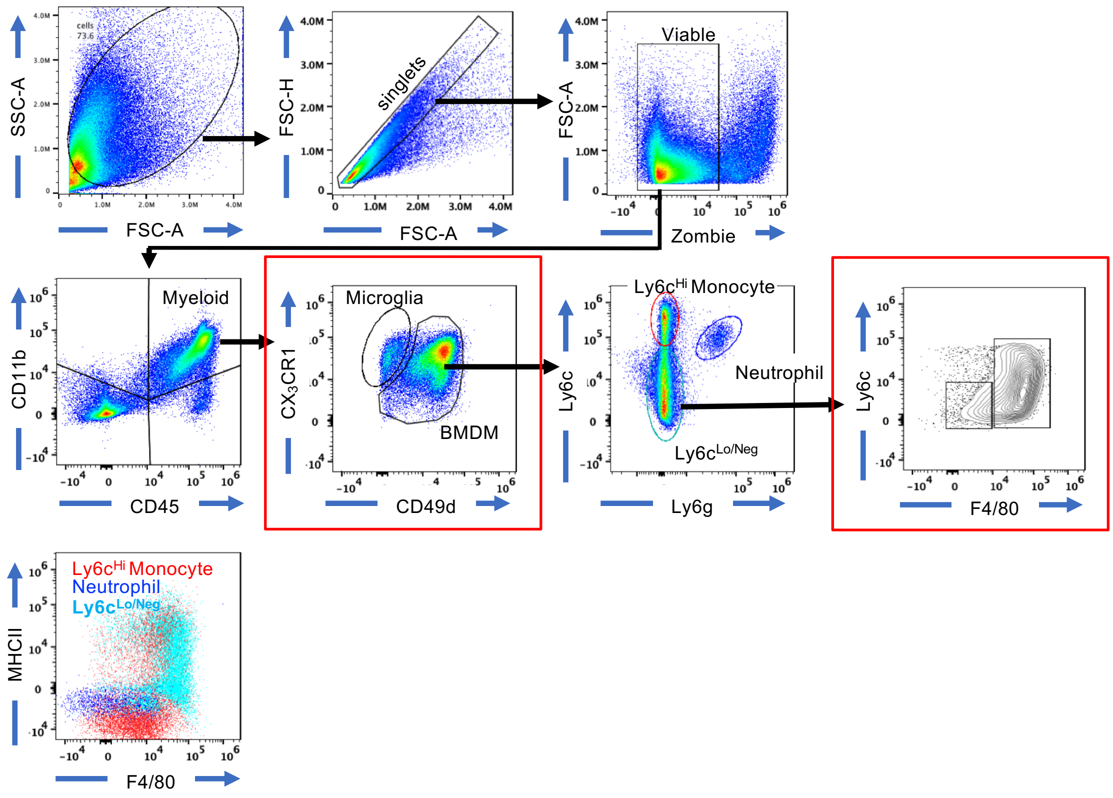

Cell types	Marker combination
Monocyte	CD11b^{+}CD45^{Hi}Ly6c^{Hi}Ly6g^{Neg}CD49d^{+}
BMDM	CD11b^{+}CD45^{Hi}CX$_{3}$CR1$^{+/-}$CD49d^{+}Ly6c$^{Lo/Neg}$Ly6g^{Neg}F4/80^{+}
Microglia	CD11b^{+}CD45^{Lo}CX$_{3}$CR1^{Hi}Ly6c^{Neg}Ly6g^{Neg}CD49d^{Neg}
Neutrophil	CD11b^{+}CD45^{+}Ly6c^{+}Ly6g^{+} CD49d^{+}

Supplementary Figure 5. Gating strategy for multiplex Aurora spectral flow cytometry panel for myeloid cell subsets. BMDM= bone marrow-derived myeloid cells.

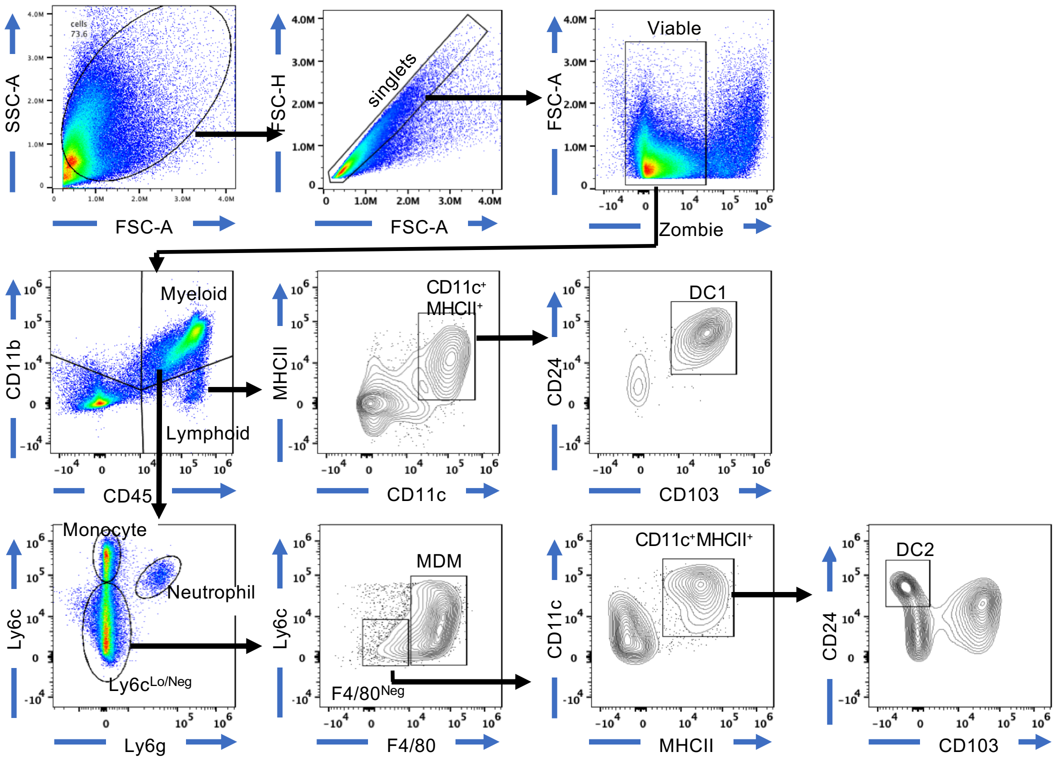

Cell type	Marker combination
**DC1**	CD11b^{Neg}CD45^{Hi}CD11c^{+}MHCII^{+}CD103^{+}CD24^{+}
**MDM**	CD11b^{+}CD45^{Hi}Ly6c$^{Lo/Neg}$Ly6g^{Neg}F4/80^{+}
**DC2**	CD11b^{+}CD45^{Hi}Ly6c$^{Lo/Neg}$Ly6g^{Neg}F4/80^{Neg}CD103^{+}CD24^{+}

Supplementary Figure 6. Gating strategy for multiplex Aurora spectral flow cytometry panel of DC1 and DC2. MDM: monocyte-derived macrophage.

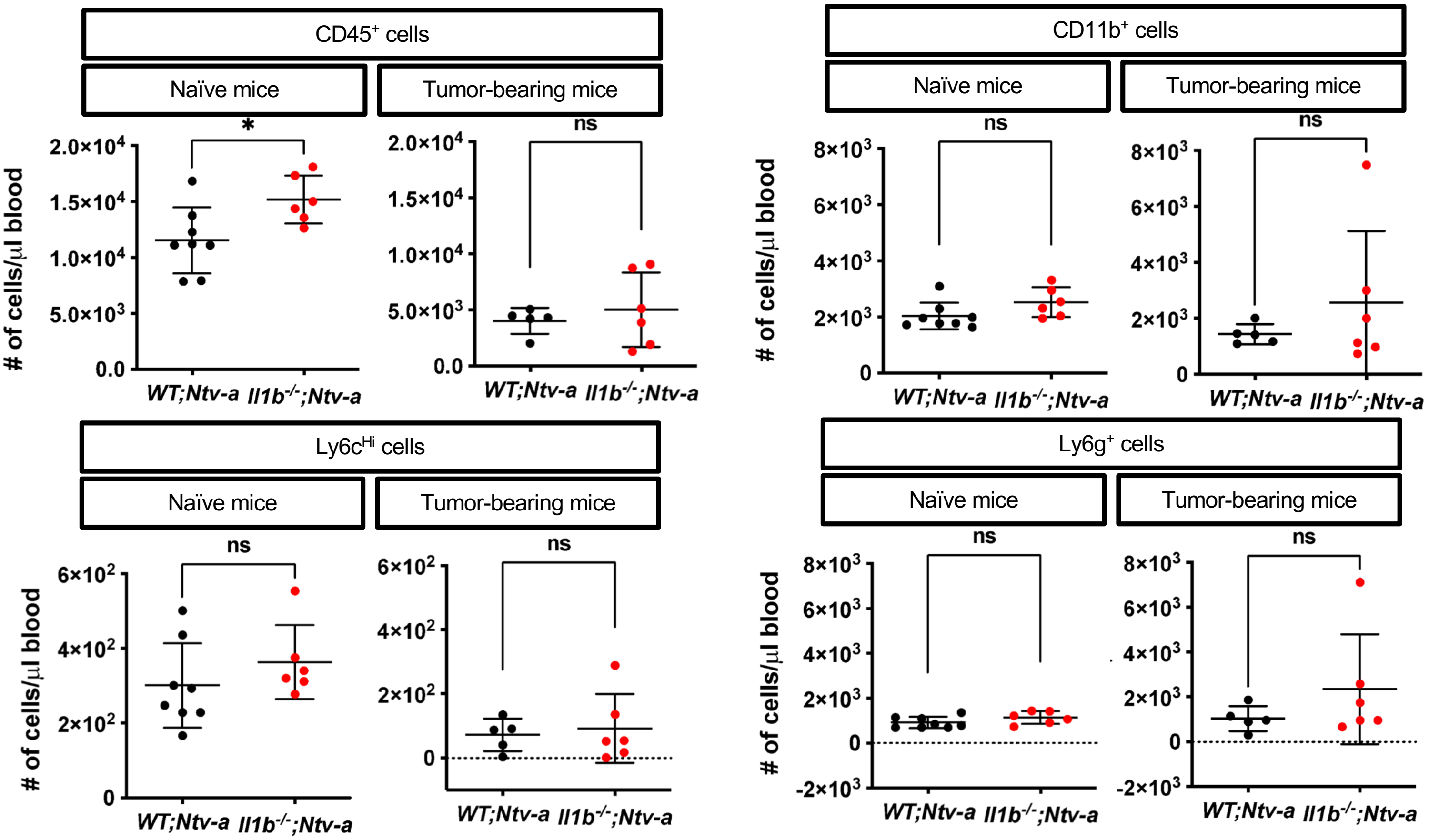

Supplementary Figure 7. ***Il1b*** **ablation has no impact on myeloid composition in the blood of naïve or tumor-bearing mice.** Quantification of FACS analysis of myeloid cell populations in naïve (N=8 and 6) and *PDGFB*-driven tumor-bearing mice (N=5 and 6) in *WT;Ntv-a* and *Il1b*$^{-/-}$*;Ntv-a* mice. Two-tailed Student's *t*-test, *P<0.05, ns = not significant.

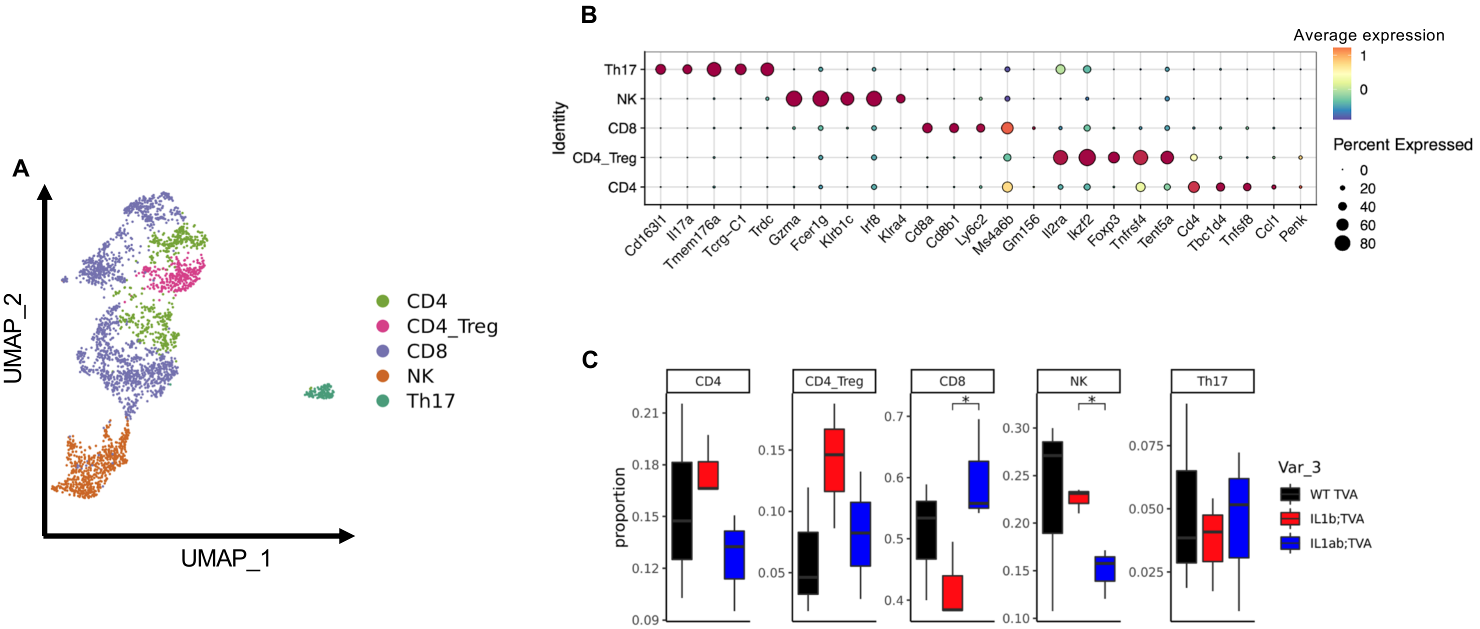

Supplementary Figure 8. ScRNA-seq analysis for the lymphoid cells. (A) UMAP dimensionality reduction of the lymphoid cell. **(B)** Selected marker genes used to annotate the cell types. **(C)** Lymphoid cell subtype distribution in *WT;Ntv-a*, *Il1b*$^{-/-}$*;Ntv-a* or *Il1a*$^{-/-}$;*Il1b*$^{-/-}$*;Ntv-a* mice. *P<0.05 by Student's *t*-test.

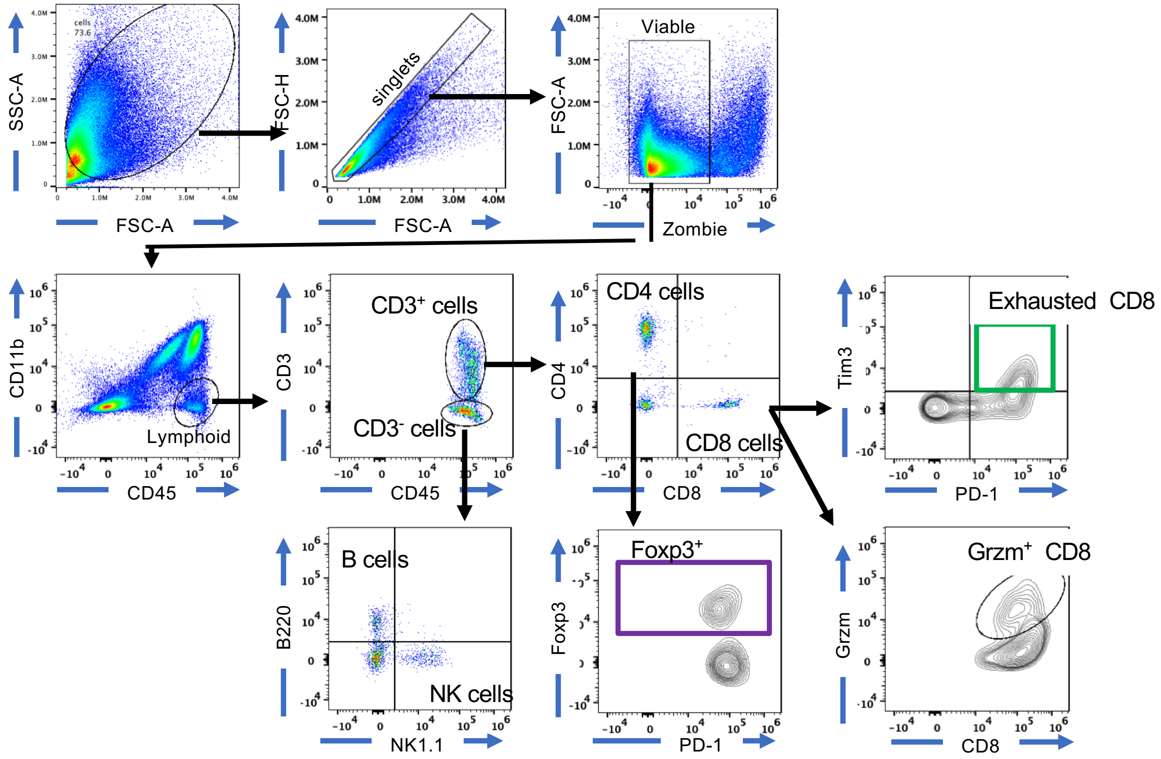

Cell type	Marker combination
**Foxp3+ T_{reg}**	$CD45^{+}CD11b^{Neg}CD3^{+}CD4^{+}CD8^{Neg}Foxp3^{+}$
**Exhausted CD8**	$CD45^{+}CD11b^{Neg}CD3^{+}CD8^{+}CD4^{Neg}PD1^{+}Tim3^{+}$
**B cells**	$CD45^{+}CD11b^{Neg}CD3^{Neg}B220^{+}NK1.1^{Neg}$
**NK cells**	$CD45^{+}CD11b^{Neg}CD3^{Neg}NK1.1^{+}B220^{Neg}$

Supplementary Figure 9. Gating strategy for multiplex Aurora spectral flow cytometry panel for lymphoid subsets. Grzm = granzyme B.

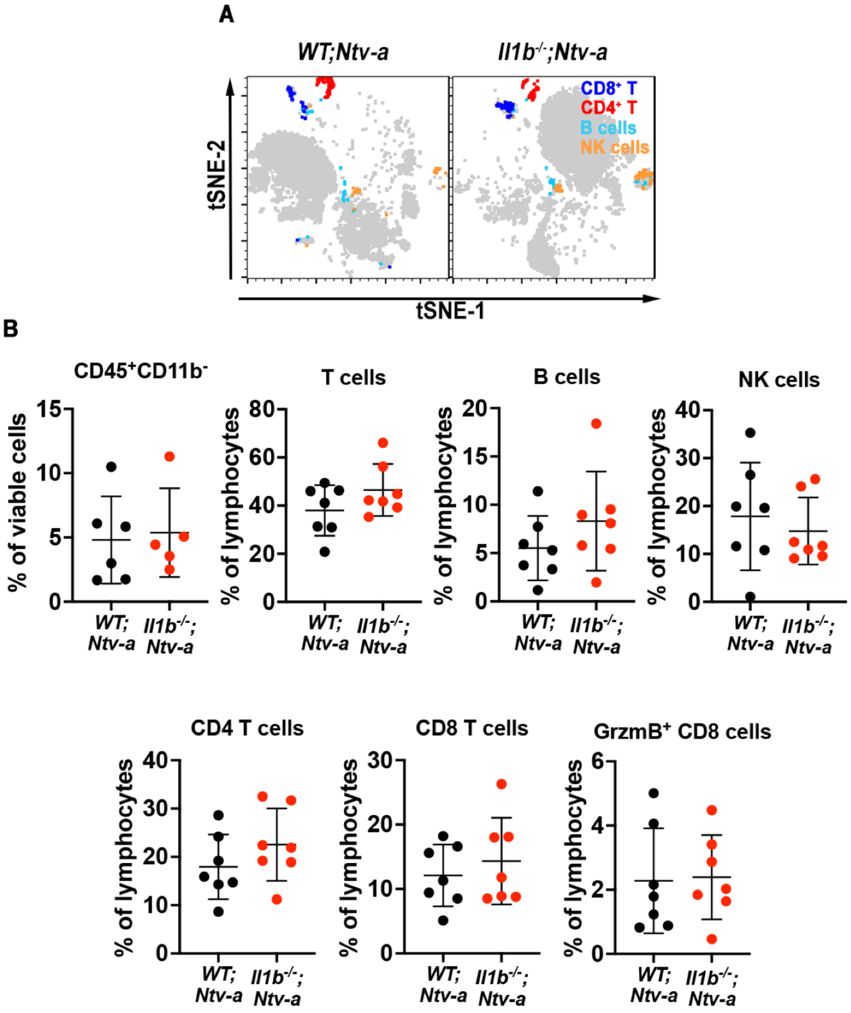

Supplementary Figure 10. ***Il1b*** **ablation has no impact on the total number of lymphoid cells in** ***PDGFB*****-driven murine GBM. (A)** tSNE plots illustrating the tumor cell/lymphoid composition in *WT;Ntv-a* (N=7) and *Il1b$^{-/-}$;Ntv-a* (N=7) mice bearing *PDGFB*-driven GBM. (**B**) Quantification of lymphoid populations in tumors generated in *WT;Ntv-a* and *Il1b$^{-/-}$;Ntv-a* mice.

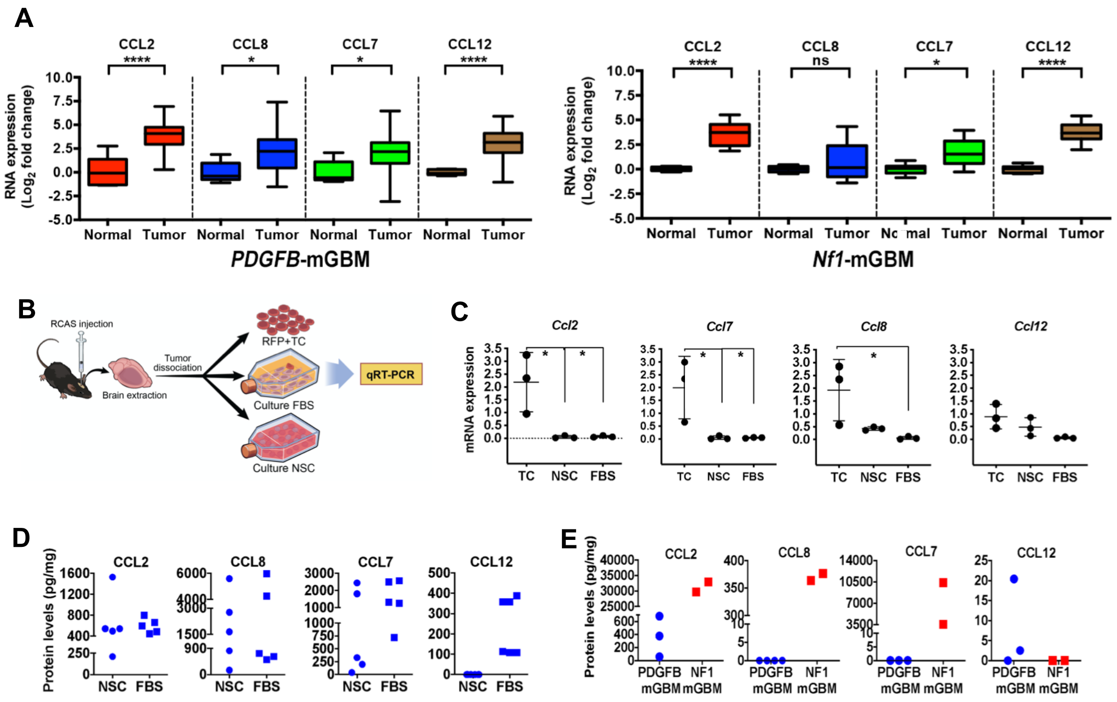

Supplementary Figure 11. MCP expression is increased in both *PDGFB* and *Nf1* mGBM. (A) qPCR analysis of MCP family members - *Ccl2, Ccl8, Ccl7* and *Ccl12* - in naïve brain (normal), *PDGFB* and *Nf1* mGBM tumors. Two-tailed Student's *t*-test, *P<0.05, ****P<0.0001, ns=not significant. **(B)** Schematic illustration of experimental steps for **(C)** demonstrating that freshly-sorted *PDGFB*-tumor cells express higher levels of MCP. When maintained in FBS-containing or NSC medium, expression levels decrease. One-way ANOVA, Tuckey's multiple comparison test, *P<0.05. TC=fresh tumor cells. N= 3 each group. **(D)** Expression of MCP proteins in early passage (P1-P2) of *PDGFB*-driven GBM cells grown in NSC or FBS conditions (N=5 each group), and **(E)** in late passage (P5 and above) *PDGFB* and *Nf1 m*GBM cells under FBS conditions (N=3 and 2 respectively). Each dot represents an independent primary culture.

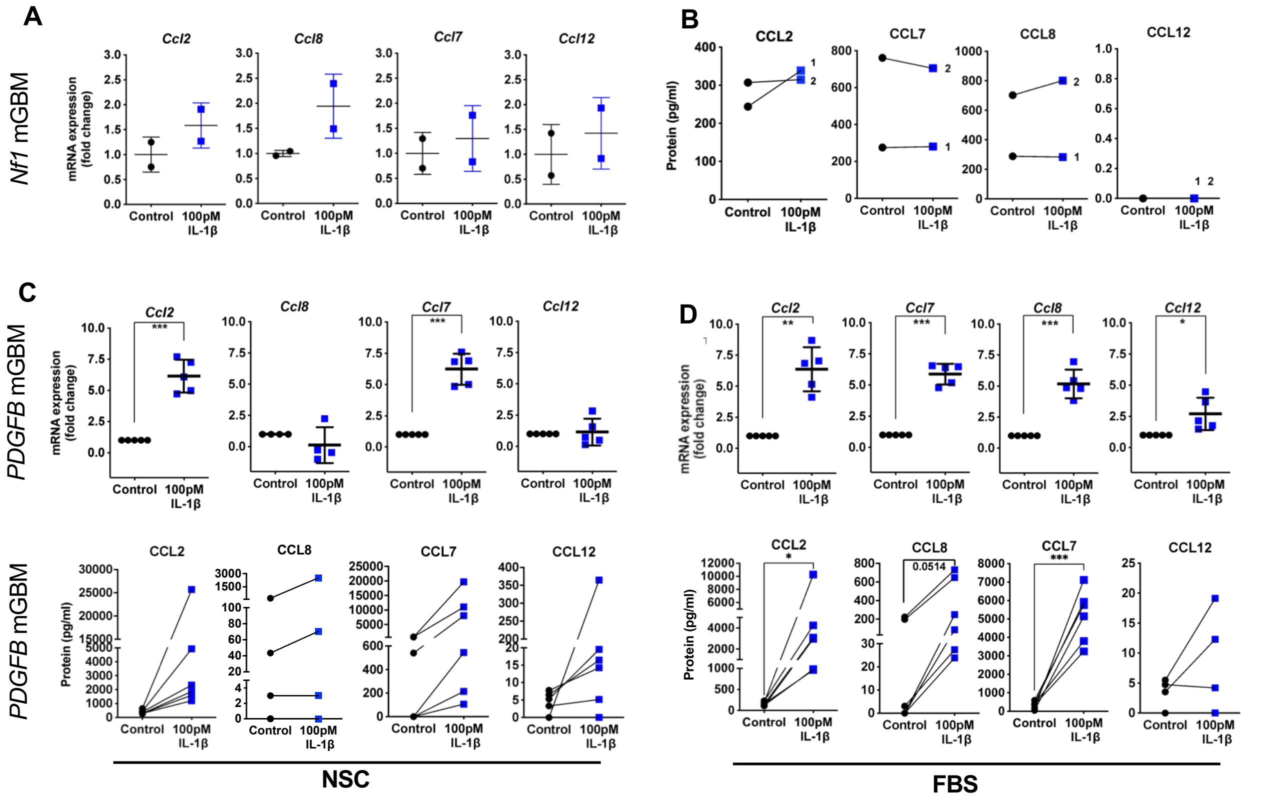

Supplementary Figure S12. IL-1β stimulation induces the MCP network in *PDGFB*-driven, but not *Nf1* mGBM cultures *in vitro*. IL-1β stimulation does not induce MCP family member **(A)** RNA, or **(B)** protein expressions in *Nf1 m*GBM cultures (N=2). Under **(C)** NSC or **(D)** FBS growth conditions, *PDGFB* primary mGBM cells respond to rIL-1β stimulation by increasing RNA and protein expressions of MCPs, as measured by qPCR (N=5) and ELISA (N=6). Two-tailed Student's *t*-test, *P<0.05, **P<0.01, ***P<0.001.

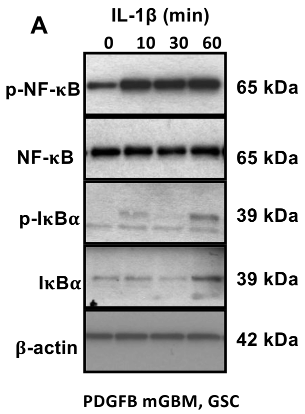

Supplementary Figure 13. IL-1β stimulation induces NF-κB pathway activation in *PDGFB* mGBM cultures under GSC condition. **(A)** Representative immunoblot showing NF-κB pathway activation in *PDGFB*-mGBM cultures under NSC conditions following rIL-1β treatment *in vitro.*

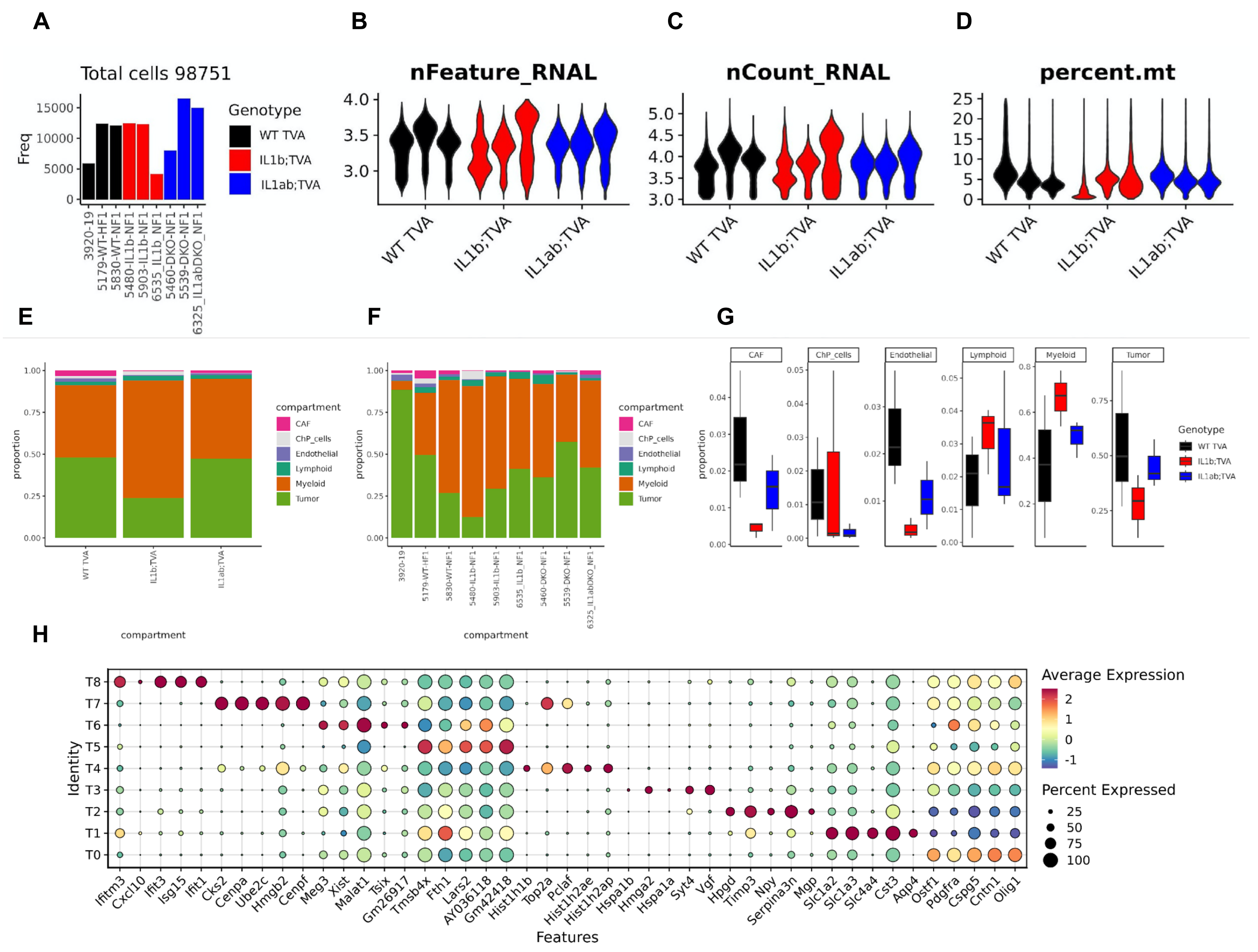

Supplementary Figure 14. Single cell RNA seq analysis of *Nf1* mGBM generated in *WT;Ntv-a*, *Il1b$^{-/-}$;Ntv-a* and *Il1a$^{-/-}$;Il1b$^{-/-}$;Ntv-a* mice. (**A**) Total number of cells per samples after removing doublets. (N=3 each group) (**B**) Distribution of number of unique molecular identifier (UMI) per cells per sample. **(C)** Distribution of number of genes detected per cell per sample. **(D)** Distribution of percentage of mitochondrial genes per cell per sample. **(F)** Proportion of the six major cell classes grouped by individual samples. **(G)** Boxplot showing the distribution of the five major cell classes in the three genotypes. CAF: cancer-associated fibroblast. ChP: choroid plexus. **(H)** Signature gene sets used to cluster the malignant tumor cells in *Nf1* mGBM.

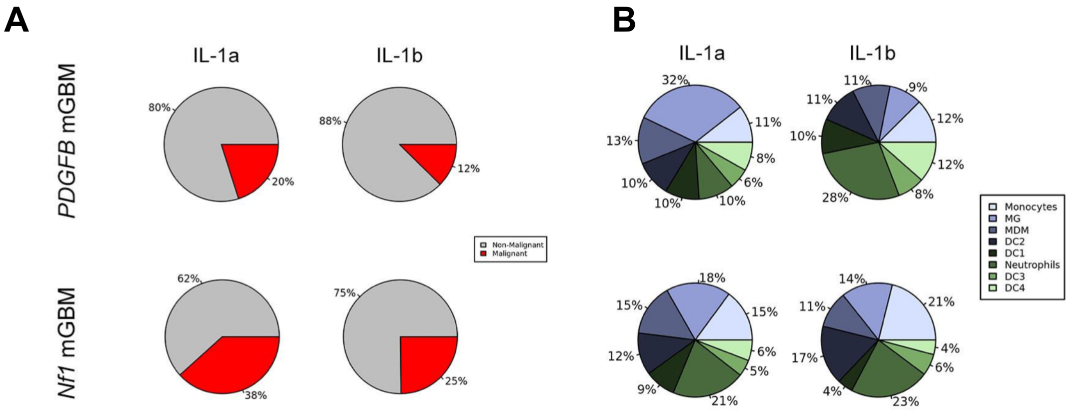

Supplementary Figure 15. scRNA-seq reveals *Il1* expression in various cell types in *PDGFB* or *Nf1* mGBM. (A) Pie-charts showing *Il1* transcripts in malignant or non-malignant compartments. **(B)** Pie-charts showing *Il1* transcripts in myeloid subsets. N=3 each group.

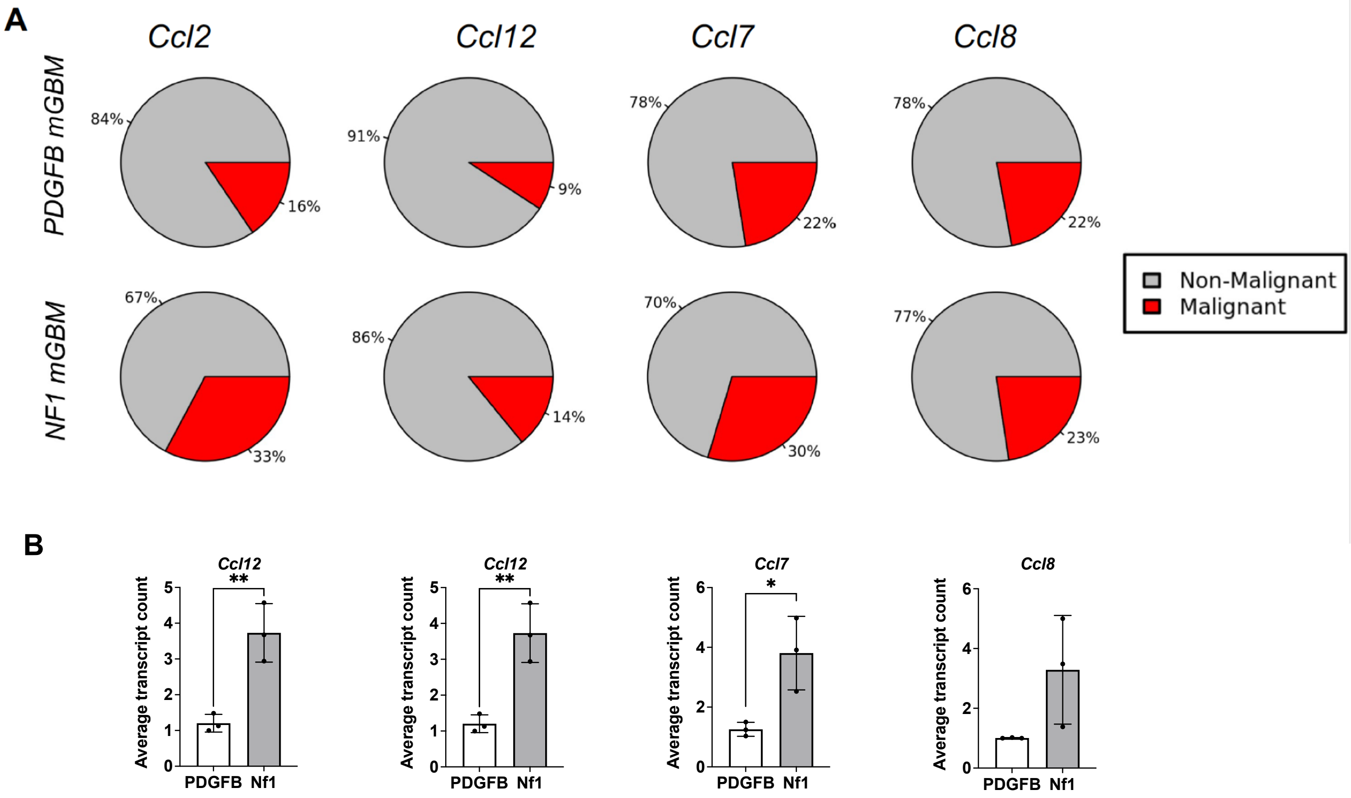

Supplementary Figure 16. scRNA-seq reveals expression of MCPs in malignant or non-malignant cells in *PDGFB* or *Nf1* mGBM. (A) Pie-charts showing MCP transcripts in malignant or non-malignant compartments. **(B)** Bar charts showing MCP transcripts in malignant cells in *PDGFB* or *Nf1* mGBM. N=3 each group.

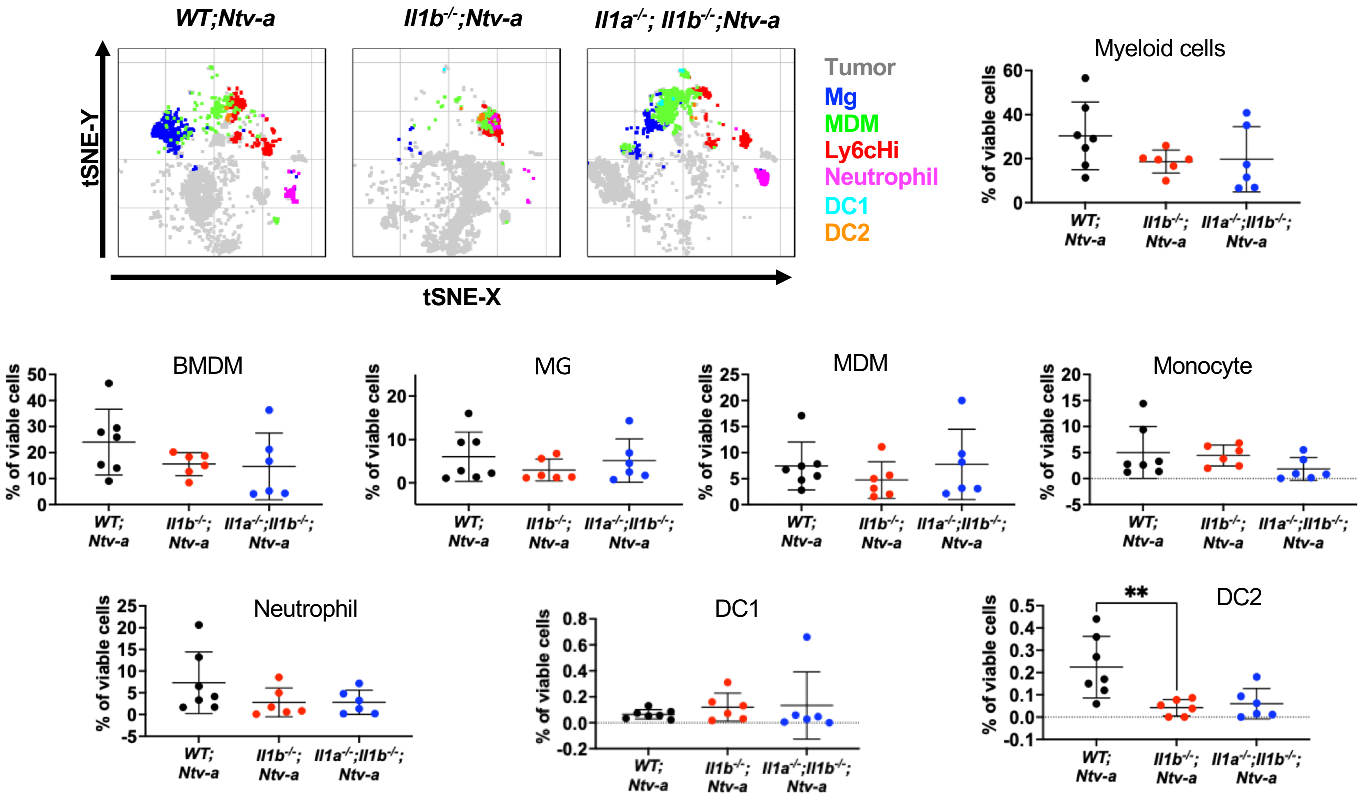

Supplementary Figure 17. Spectral flow cytometry analysis of myeloid cells in *Nf1* mGBM. **P<0.01, by one-way ANOVA followed by Tucky's post-hoc comparison. BMDM: bone marrow-derived myeloid cells, MG: microglia, MDM: monocyte-derived macrophage, DC: dendritic cells. N=7, 6 and 6 respectively.

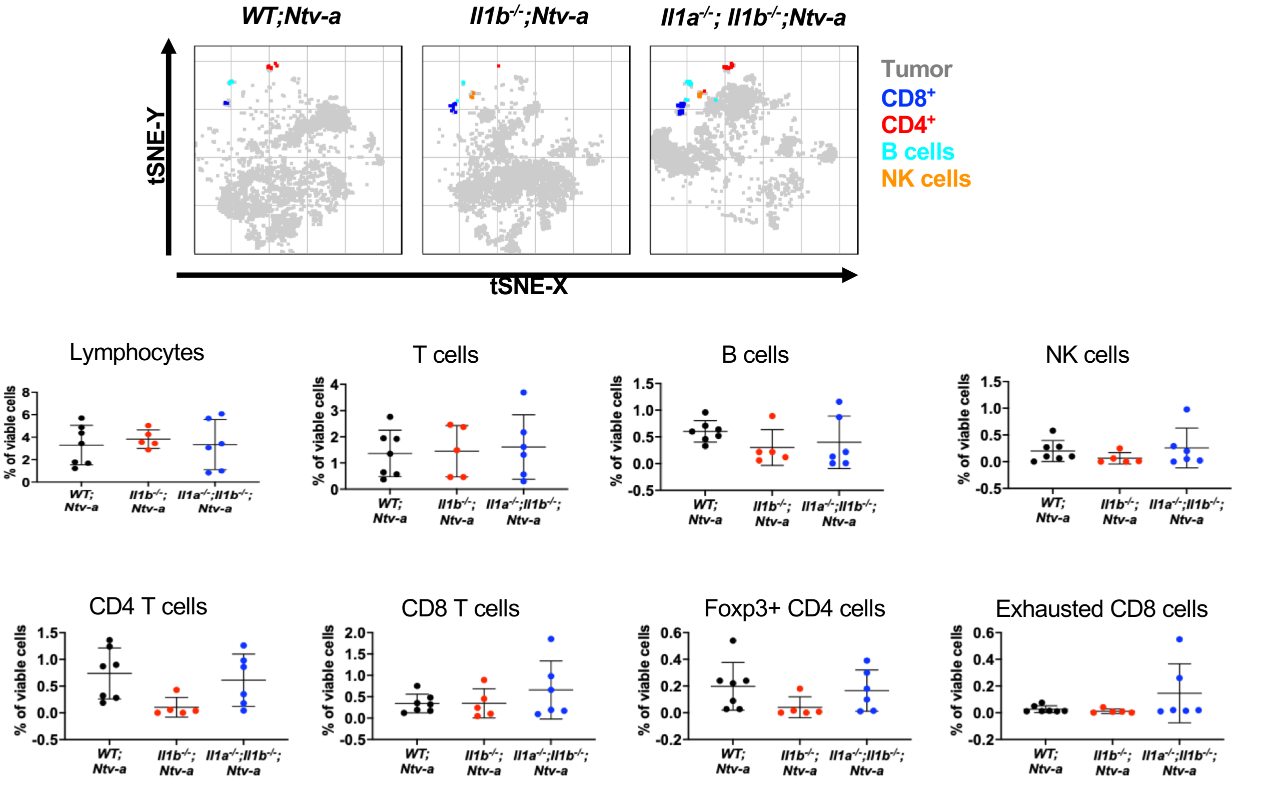

Supplementary Figure 18. Spectral flow cytometry analysis of lymphocytes in *Nf1* mGBM. N=7, 5 and 6 respectively.

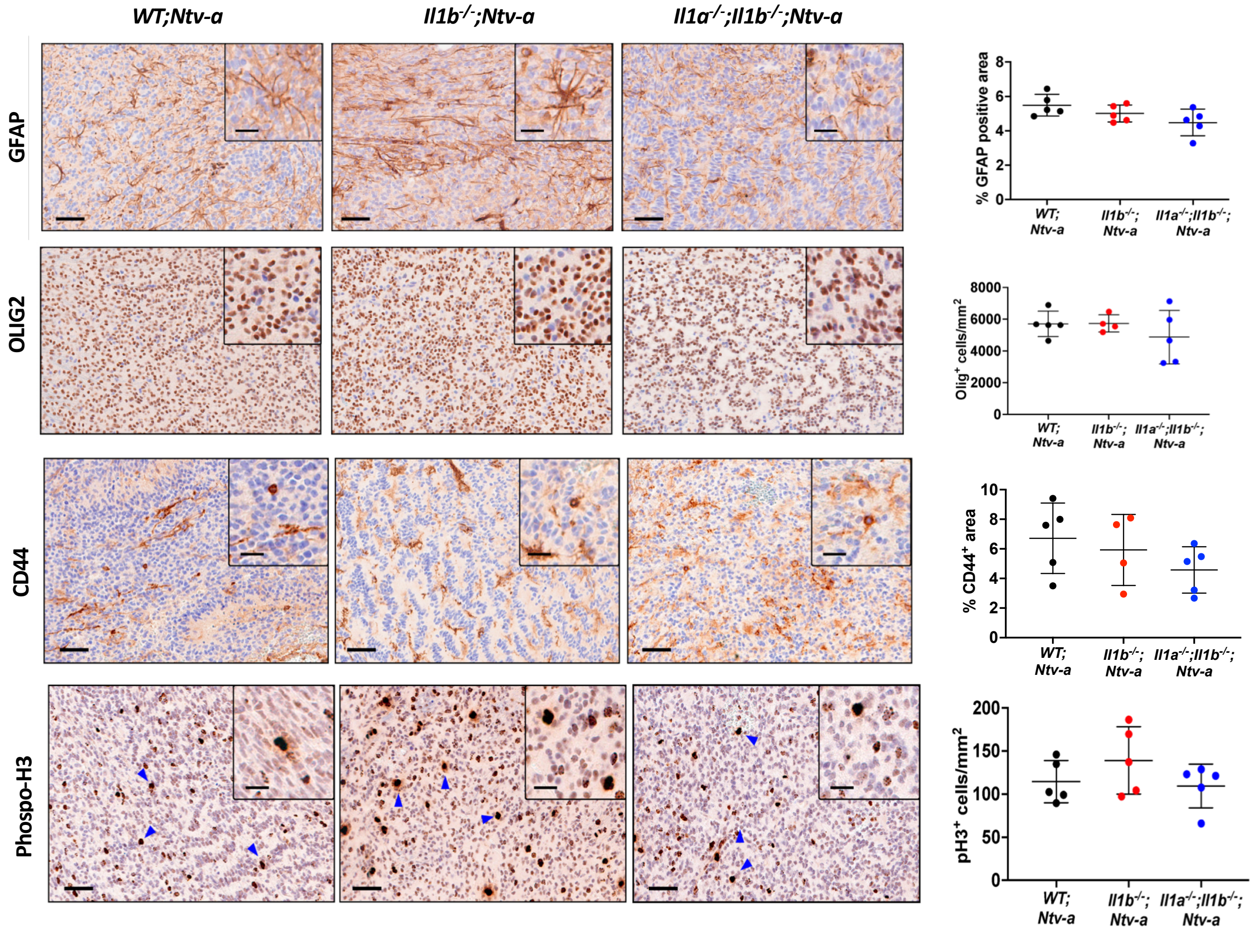

Supplementary Figure 19. Immunohistochemical staining of *Nf1* mGBM tissues. *P<0.05 by one-way ANOVA followed by Tucky's post-hoc comparison. Scale bar in main graph = 50 μm, in insets = 20 μm. N=5 each group.

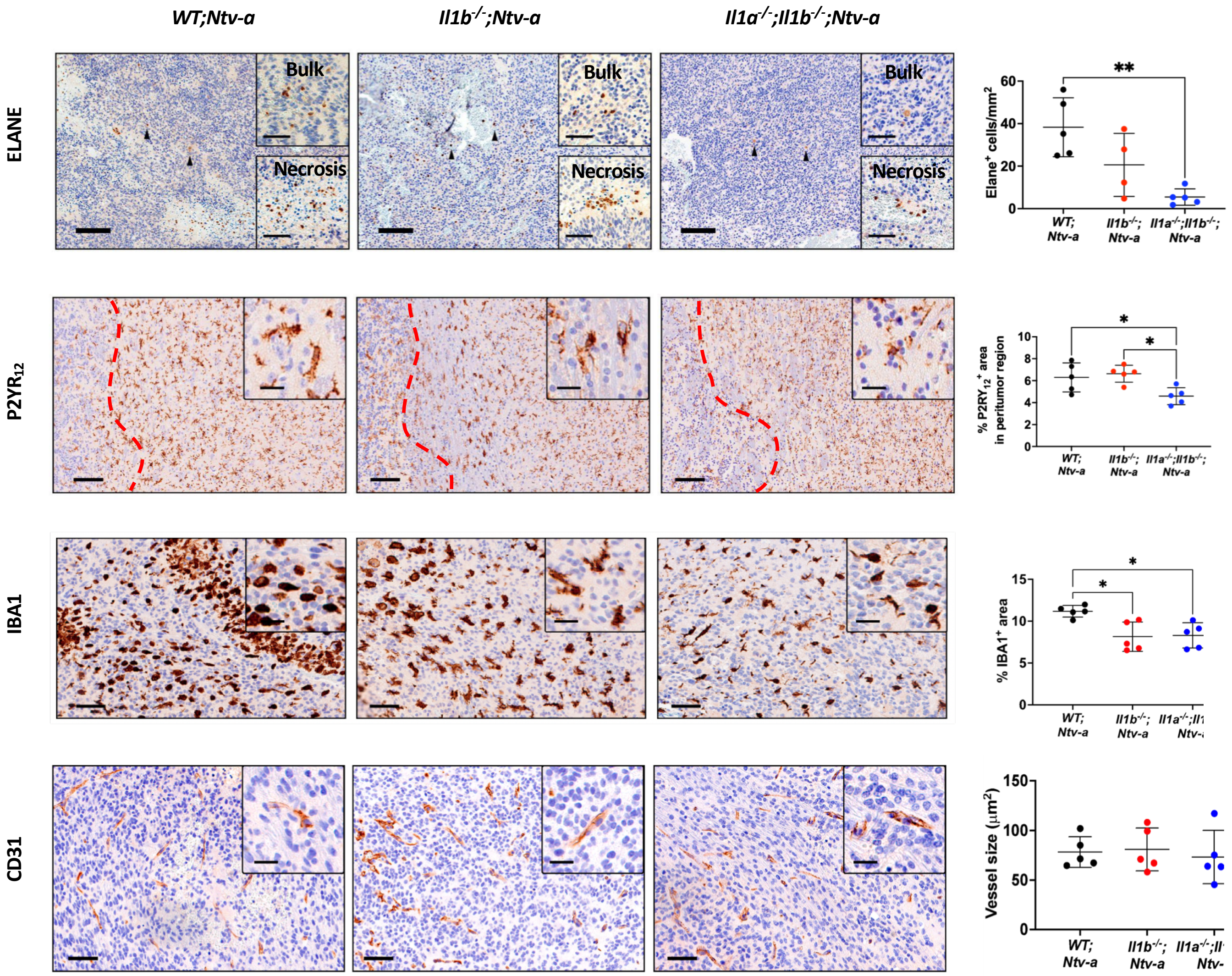

Supplementary Figure 20. Immunohistochemical staining of *Nf1* mGBM tissues. *P<0.05, **P<0.01, by one-way ANOVA followed by Tucky's post-hoc comparison. Scale bar in main graph = 50 μm, in insets = 20 μm. N=5 each group.

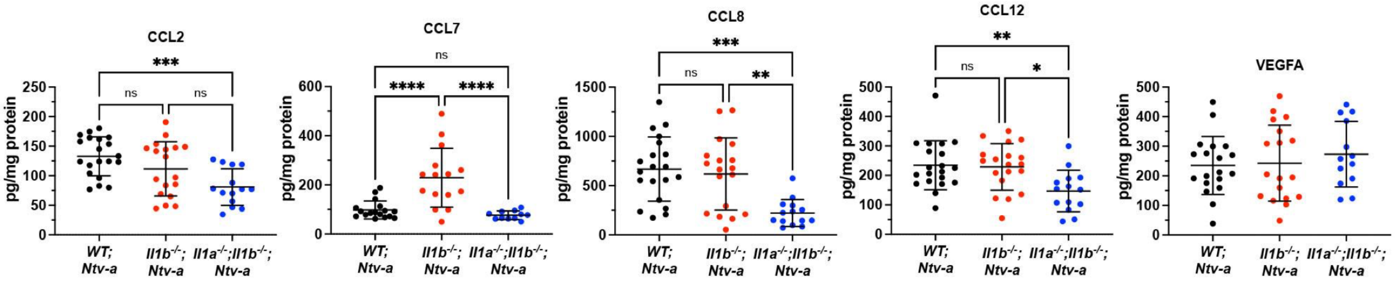

Supplementary Figure S21. *Il1* genetic deletion reduces MCP production in *PDGFB* mGBM *in vivo*. Intracellular levels of MCP members (CCL-2, -7, -8, and -12) and VEGFA were quantified by ELISA in tumors generated in *WT;Ntv-a*, *Il1b*$^{-/-}$*;Ntv-a* or *Il1a*$^{-/-}$;*Il1b*$^{-/-}$*;Ntv-a* mice at humane endpoint. VEGFA levels were not affected and were used as a negative control. One-way ANOVA with Tukey's *post-hoc* comparisons. *P<0.05; **P<0.01; ***P<0.001; ****P<0.0001; ns = not significant. N=20, 18 and 14, respectively.

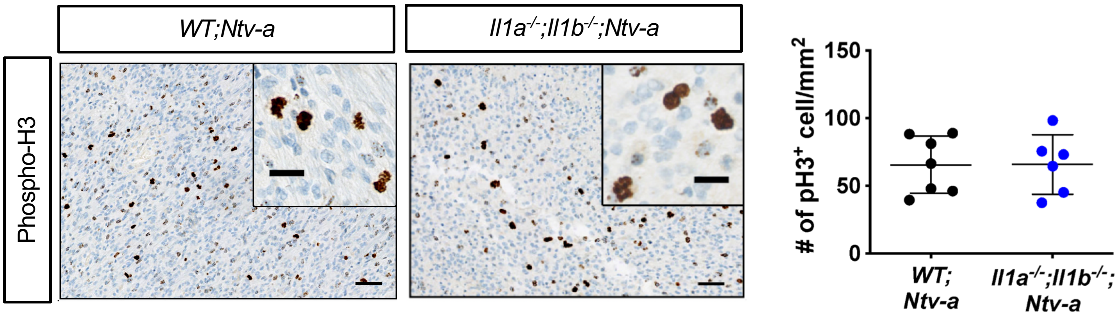

Supplementary Figure 22. Representative images of Immunohistochemical staining and corresponding quantification of phosphorylated histone H3 in *PDGFB* mGBM generated in *WT;Ntv-a* and *Il1a$^{-/-}$; Il1b$^{-/-}$;Ntv-a* mice. Scale bar = 50 µm; scale bar of inset = 20 µm. Student's *t*-test was used for statistical comparison. N=7 and 6.

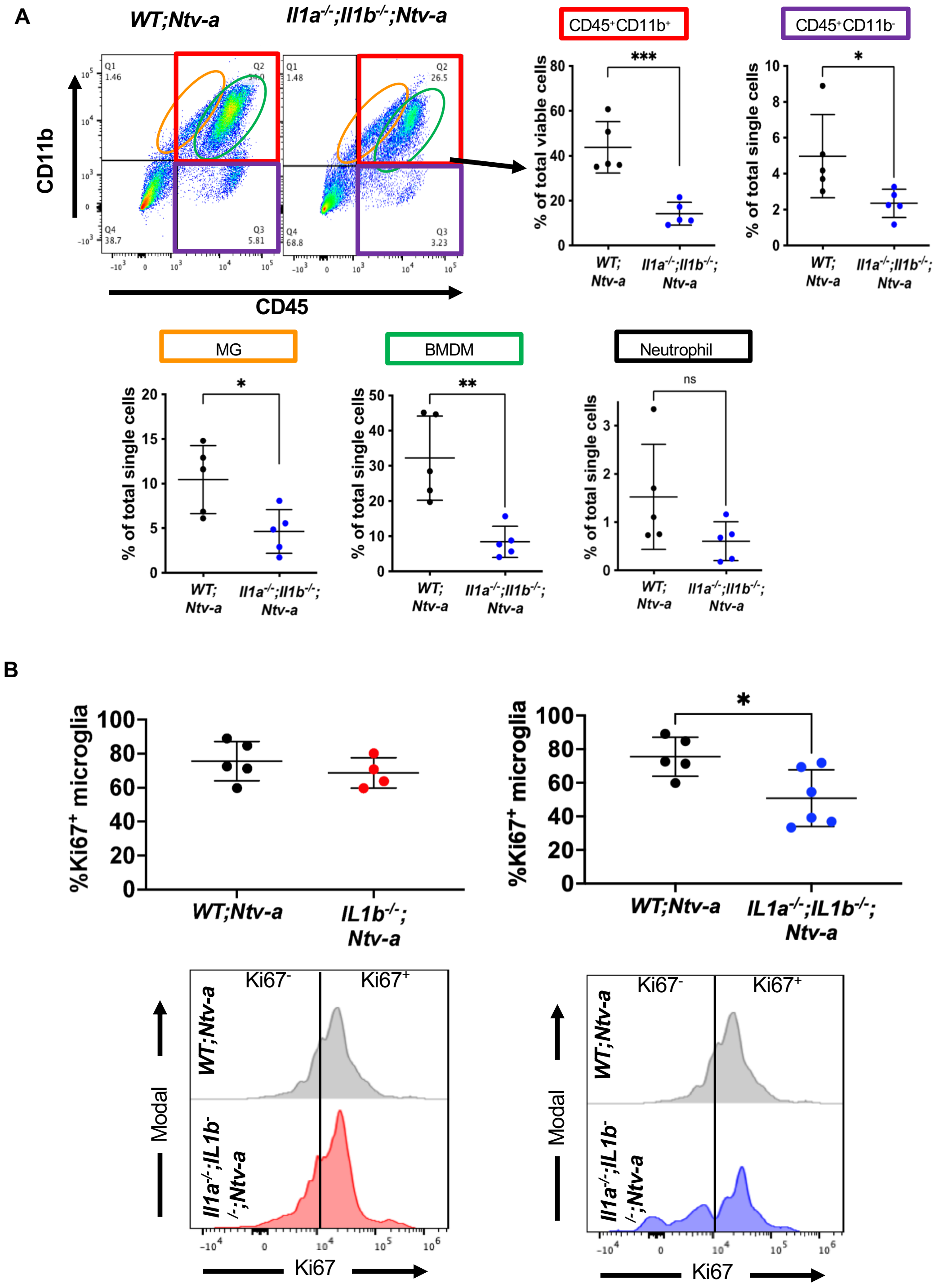

Supplementary Figure 23. Flow cytometry analyses of immune cells in *WT;Ntv-a* and *Il1b*$^{-/-}$*;Ntv-a* mice. (A) Flow cytometry dot plots and quantification of myeloid cells between *WT;Ntv-a* and *Il1b*$^{-/-}$*;Ntv-a* mice. N=5 each group **(B)** Analysis of proliferating microglia in *PDGFB* mGBM. Two-tailed Student's *t*-test, *P<0.05, **P<0.01; ***P<0.001, ns = not significant. N=5, 4 and 6, respectively.

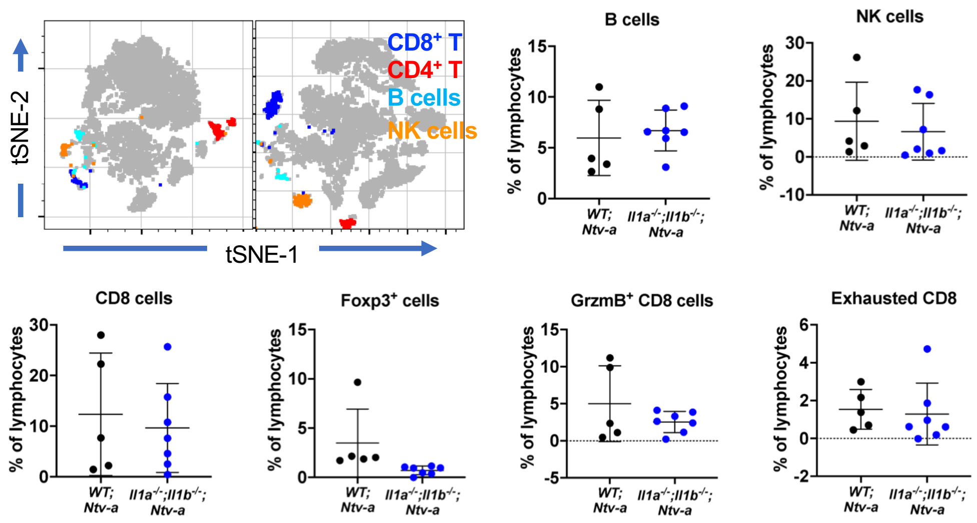

Supplementary Figure 24. Flow cytometry analyses of immune cells in *WT;Ntv-a* and *Il1a$^{-/-}$; Il1b$^{-/-}$;Ntv-a* mice. tSNE plots and quantification of lymphocytes between *WT;Ntv-a* and *Il1a$^{-/-}$; Il1b$^{-/-}$;Ntv-a* mice. N=5 and 7.

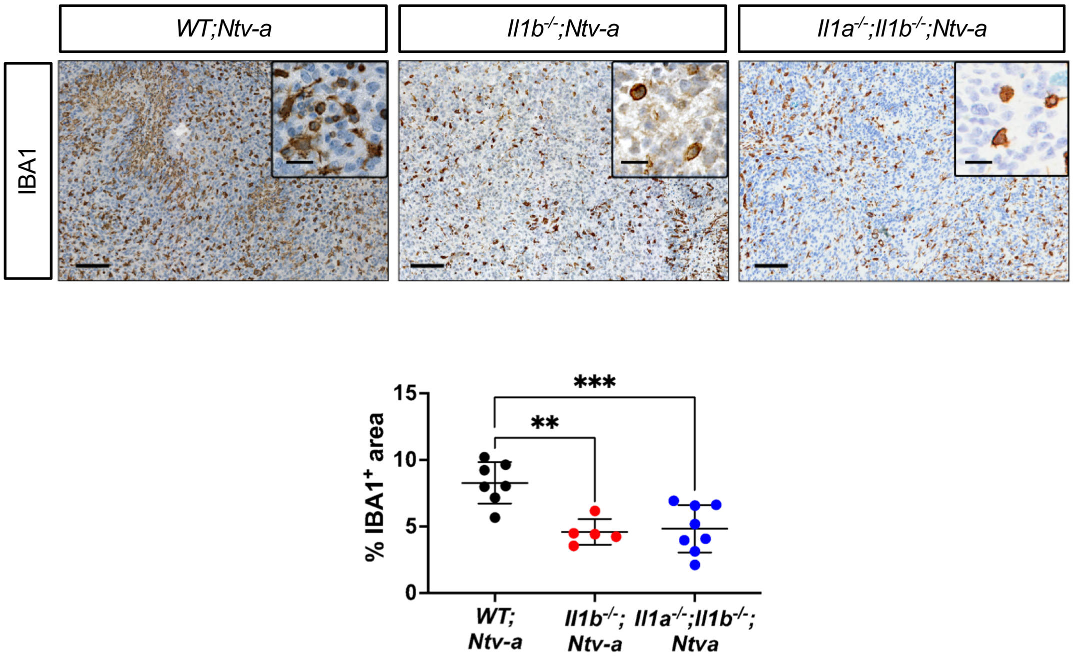

Supplementary Figure 25. Loss of *Il1b* and *Il1* leads to decreased macrophages positive area. Representative images and IHC quantification for IBA1. Scale bar = 50 µm; scale bar of inset = 20 µm. One way ANOVA with Tukey's *post-hoc* test. **P<0.01, ***P<0.001. N=7, 5 and 8.

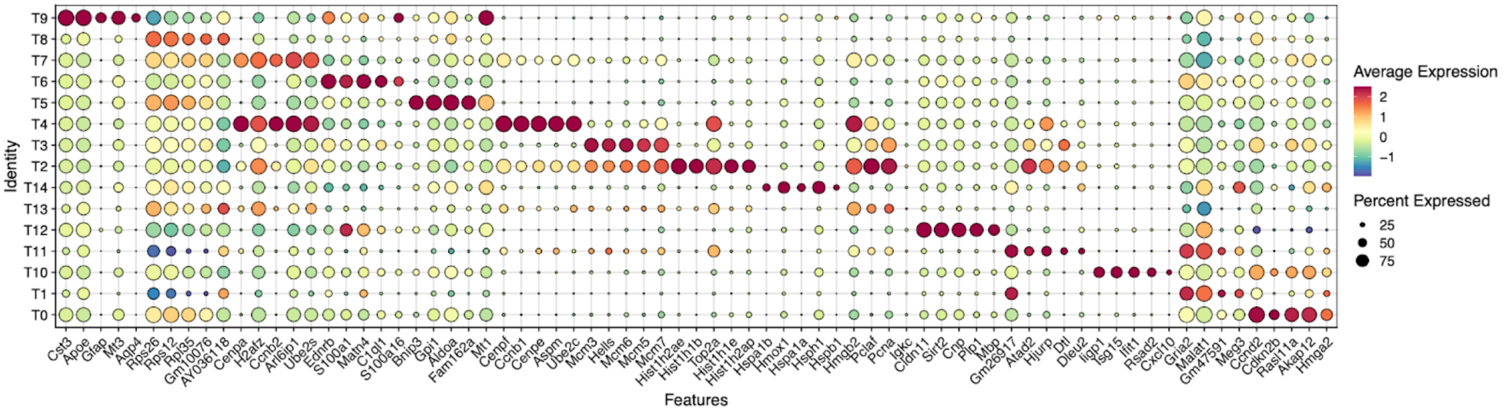

Supplementary Figure 26. Signature gene sets used to cluster the malignant tumor cells in PDGFB mGBM in *WT;Ntv-a* mice.

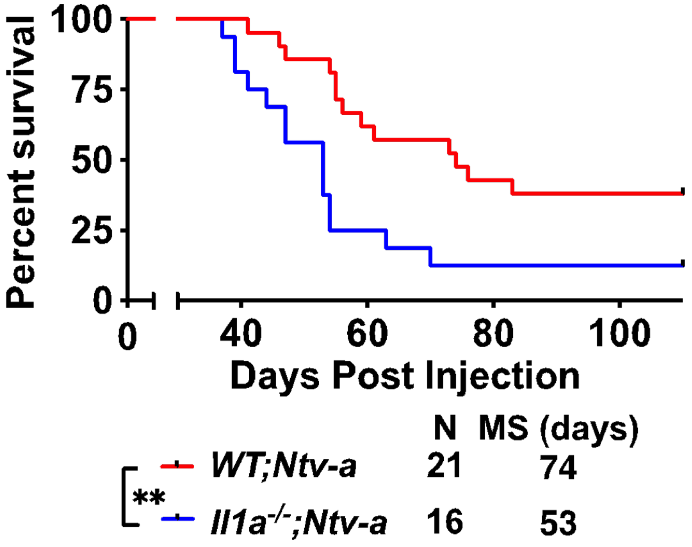

Supplementary Figure 27. Kaplan-Meier survival curves comparing *WT;Ntv-a* and *IL1a*$^{-/-}$*;Ntv-a* mice bearing *PDGFB* mGBM. MS = median survival. N = number of mice. **P<0.01 by Log-rank test.

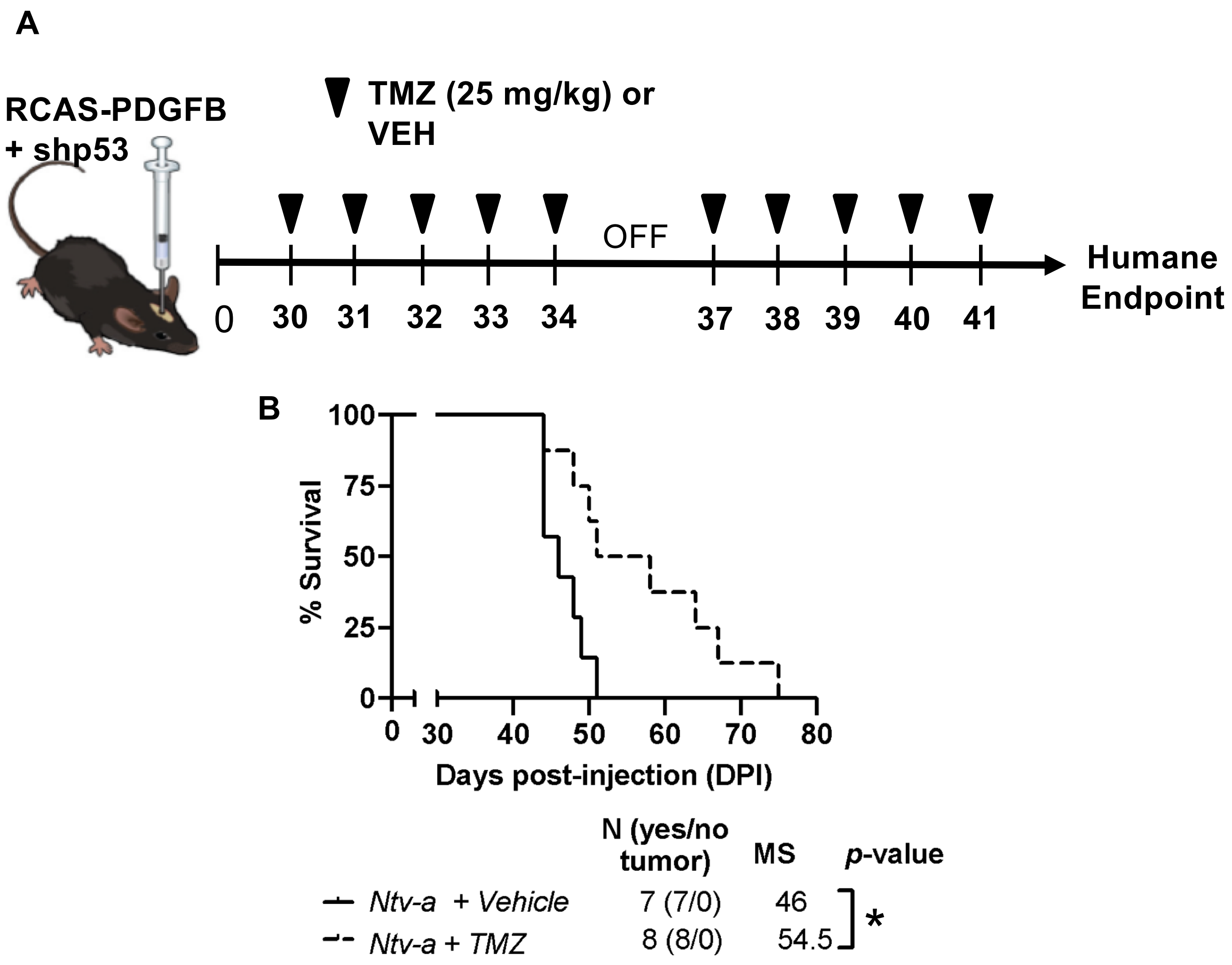

Supplementary Figure 28. TMZ as standard-of-care treatment extends the survival of tumor-bearing mice by 8.5 days. (A) Illustration of experimental scheme with current clinical standard-of-care drug TMZ. (**B**) Kaplan-Meier survival analysis of TMZ treatment. MS = median survival. *P<0.05 by Log-rank test.

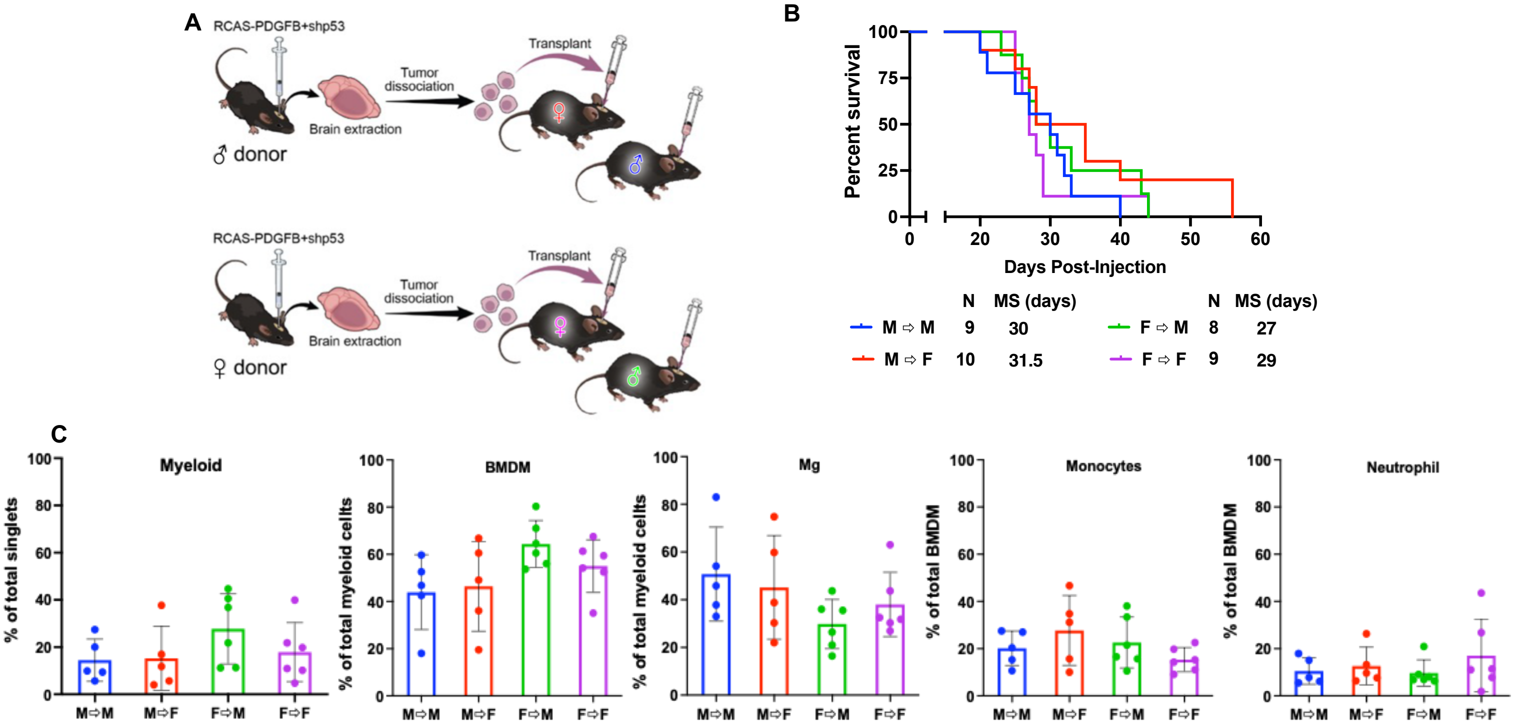

Supplementary Figure 29. The gender of the tumor donor or recipient animals was not associated with differential recruitment of myeloid populations and did not impact survival in PDGFB-tumor-bearing mice. (A) Schematic illustration of generating of male or female tumor-bearing mice receiving tumors from male or female donors. **(B)** Kaplan-Meier survival curves. MS=median survival. N=number of mice. **(C)** FACS analysis of myeloid cell populations in female and male recipient animals following transplantation with male and female donor-derived tumor cells.

Supplemental Methods

Mice

Mice of both sexes (equal distribution) in the age range of 8-16 weeks were used for experiments (1, 2). Previously-described *Il1b$^{-/-}$,* and *Il1a/b$^{-/-}$* mice were crossed with *Ntv-a* mice to generate *Il1b$^{-/-}$;Ntv-a*, *Il1a$^{-/-}$;Ntv-a* and *Il1a$^{-/-}$;Il1/b$^{-/-}$;Ntv-a* mice (3-5). *Il1b* mutant mice referred as *Il1b$^{-/-}$* express *RNA* for *Il1b* but both *Il1b$^{-/-}$,* and *Il1a/b$^{-/-}$* mice lack protein expression (6). Mice used in this study are all in a C57BL/6J background, except for *Ntv-a;Cdkn2a$^{-/-}$;Pten$^{fl/fl}$*, which is in a mixed genetic background. C57BL/6J mice (000664) at 8-12 weeks old were purchased from the Jackson labs and used for BMDM isolation. All animals were housed in a climate-controlled, pathogen-free facility with access to food and water *ad libitum* under a 12-hour light/dark cycle. Tumor-bearing mice were euthanized at humane endpoint to ensure comparable tumor burden across all animals. Humane endpoint was defined as the terminal stage of life where the mice exhibit symptoms including head tilt, lethargy, seizures, and excessive weight loss (10-15% total loss). All experimental procedures were approved by the Institutional Animal Care and Use Committee (IACUC) of Emory University (Protocol #201700633) and the Icahn School of Medicine at Mount Sinai (Protocol #201900619).

Virus generation and tumor induction

We delivered RCAS-*PDGFB* in *Cdkn2a$^{-/-;}$Ntv-a* mice, or the combination RCAS-*PDGFB* with RCAS-shRNA-*p53-Rfp* in *Ntv-a* mice, both of which have been shown to closely resemble human PN GBM in terms of histological and molecular features (1, 2). To generate a murine MES model, we chose to silence the tumor suppressor genes NF1, TP53, and PTEN by co-injection of RCAS virus caring short hairpin RNA of these molecular targets, along with introducing PDGFA, as previously described (2). To propagate RCAS viral vectors, DF-1 cells (ATCC, CRL-12203) were purchased and grown at 39°C according to the supplier's instructions. The cells were routinely tested for mycoplasma contamination to assure they are negative for mycoplasma. Cells were transfected with RCAS-*hPDGFB-HA,* RCAS shRNA*Nf1,* RCAS shRNA*Pten,* and RCAS-shRNA-*p53-Rfp* using a Fugene 6 transfection kit (Roche, 11814443001) according to the manufacturer's instructions. DF-1 cells ($4x10^4$) in 1 µl neurobasal medium were stereotactically delivered with a Hamilton syringe equipped with a 30-gauge needle for tumor generation (7). The target coordinates were in the right-frontal striatum at AP 1.0 mm and right 1.5 mm from bregma; depth 1.5 mm from the dura surface (1, 2, 7). Mice were continually monitored for signs of tumor burden and were sacrificed upon observation of endpoint symptoms including head tilt, lethargy, seizures, and excessive weight loss.

Orthotopic glioma generation

The same procedure was used as described above, except $3x10^4$ of freshly-dissociated tumor cells were injected in the right-frontal striatum AP 1.0 mm and right 1.5 mm from bregma; depth 1.5 mm from the dura surface of recipient animals. Two or three donor tumors of either sex were used to obtain single cell suspension for orthotopic glioma generation in male and female recipient animals.

Microglia and monocyte-derived macrophage isolation and culture

Microglia were isolated from postnatal day 0 (p0) to p3 pups using a modification of a previously-described protocol (8). Briefly, whole brains were extracted from p0-p3 pups using sterile instruments followed by consecutive washes in DPBS. Brains were then digested in 0.5% trypsin (ThermoFisher, 15400054) and DNase I (Sigma-Aldrich, 11284932001) followed by mechanical dissociation prior to filtration with a 70 µm cell strainer. The filtrate was plated in flasks pre-coated with poly-D-lysine (Sigma-Aldrich, P6407) in DMEM (ThermoFisher, 10569010) with 10% FBS (HyClone, SH30396.03) and M-CSF (Peprotech, 315-02) at a concentration of 40 ng/ml. The cells grew until the astrocytes reached confluence on the bottom of the flask and microglia were observed ballooning off of the surface. During this time, additional M-CSF was periodically added. Microglia were collected by gently rinsing the back of the flask with culturing media and were plated in 6-well plates in 2 ml of the culturing media with M-CSF at a concentration of 40 ng/ml for experimentation.

C57BL6/J mice (8-12 weeks of age) were euthanized via CO_2 asphyxiation. The whole, intact femur and tibia were stripped of muscle and collected in sterile Dulbecco's phosphate-buffered saline (DPBS, ThermoFisher, 14190-250). Both ends of the bone were cut and the marrow was flushed into a clean petri dish using DPBS supplemented with 4% bovine serum albumin (BSA, ThermoFisher, 15260037), heparin (StemCell, 07980), DNase I (Sigma-Aldrich, 11284932001), and penicillin/streptomycin (ThermoFisher, SV30010). The resulting mixture was briefly triturated and passed through a 70 µm cell strainer. The cells were plated in a 15 cm non-cell culture-treated plate for a six-day differentiation in 15 ml of DMEM (ThermoFisher, 10569010) with 10% fetal bovine serum (FBS; HyClone, SH30396.03) and 40 ng/ml macrophage colony-stimulating factor (M-CSF, Peprotech, 315-02). An additional 15 ml of media with M-CSF was added after day three. The cells were harvested for experimentation via a 10-minute incubation in DPBS with 5mM EDTA on ice. Cells were plated in 6-well plates in 2 ml of culturing media with M-CSF at a concentration of 20 ng/ml for experimentation.

Organotypic tumor slice culture

Ntv-a mice harboring *PDGFB*-overexpressing and *p53*-silenced tumors were euthanized at endpoint via carbon dioxide asphyxiation. The brain was rapidly extracted and embedded in 4% low-melt agarose in PBS. The embedded brain was then mounted on a vibratome (Leica, 1220S) and submerged in ice cold Hank's balanced salt solution (HBSS, Gibco 14185052). The brain was cut into 300 µm-thick sections and the slices were transferred to inserts in a 6-well plate. Slices were cultured in Neurobasal media (StemCell, 05700) supplemented with B27 supplement (ThermoFisher, 17504044), sodium pyruvate (ThermoFisher, 11360070), and glutamine (ThermoFisher, 35050061). M-CSF (BioLegend, 576406) at a concentration of 40 ng/ml was included in the media during co-culture experiments with BMDM.

Tumor dissociation and primary cell culturing

Tumor dissociation was performed as previously described. Briefly, animals at humane endpoint were anesthetized with an overdose of ketamine/xylazine mix and transcardially perfused with ice-cold sterile Ringer's solution. Tumors were dissected from the brain, minced into pieces < 1 mm^3, and digested with an enzymatic mixture that includes papain (0.94 mg/ml, Worthington, LS003120), EDTA (0.18 mg/ml, Sigma, E6758), cysteine (0.18 mg/ml, Sigma, A8199), and DNase I

(60 µg/ml, Roche, 11284932001) in 2 ml HBSS (Gibco, 14175-095). Tumor tissues were kept at 37°C for 15 minutes with occasional agitation. The digestion was terminated with the addition of 2 ml Ovomucoid (0.7 mg/ml, Worthington, LS003086). Following digestion, single cells were pelleted, resuspended in HBSS, and centrifuged at low speed (84 RCF) for 5 min, before passing through a 70 µm cell strainer.

For tumorsphere cultures (NSC), cells were seeded at 5 x 10^5 cells/ml and grown in Neurocult mouse neurobasal medium (Stem Cell Technologies, 5700) supplemented with 10 ng/ml hEGF (Lonza, cc-4017FF), 20 ng/ml basic-hFGF (ThermoFisher, PHG0261), 1 mg/ml Heparin (Stem Cell Technologies, 7980), and NSC Proliferation Supplements (Stem Cell Technologies, 5701). Fresh medium was added to the cultures every 48 hours. For differentiation (FBS) cultures, cells were seeded at 5 x 10^5 cells/ml and grown in DMEM (ATCC, 30-2002) supplemented with 10% FBS (ATCC, 30-2020). Fresh medium was added to the cultures every 48 hours.

IL-1β treatment *in vitro*

For primary neurospheres, cultures (before rIL-1β stimulation) were dissociated with Accutase (Sigma-Aldrich, A6964) to generate single cells, which were then grown as adherent monolayers on coverslips (Φ=12 mm) coated with Geltrex (Life Technologies, A14132-01) prepared according to manufacturer's instructions. Cells were stimulated with 100pM rIL-1β (R&D 401-ML/CF) for period indicated in the graphs.

MES tumor cell cultures and *in vitro* stimulation

Murine MES GBM lines (1816 and 4622) were cultured as either neurospheres or FBS cultures as previously described (9-11). Mycoplasma tests (Lonza, LT07) were performed monthly to ensure no such contamination occurred in the cultures. For treatment in the presence of NF-kB inhibitors, the cells were sub-cultured at 10^5 cells/well in a 24-well plate pre-coated with Geltrex (Life Technologies, A14132-01) so that the cells grew in a monolayer fashion, evenly exposed to the compounds. To stimulate BMDM or microglia with spent medium of GBM cell lines, supernatant was collected from each well of the cultured GBM cells and transferred to BMDM or microglia cultures.

Tumor and cultured cell RNA isolation and qPCR analysis

At humane endpoint, mice were sacrificed with an overdose of ketamine/xylazine and immediately perfused with ice cold Ringer's solution (Sigma-Aldrich, 96724-100TAB). The brain was extracted, and a piece of tumor was immediately snap-frozen in liquid nitrogen for storage at -80°C. Alternatively, cultured cells were harvested from plates using TRIzol (ThermoFisher, 15596026). RNA was isolated from the frozen tumor pieces or cells with the RNeasy Lipid Tissue Mini Kit (Qiagen, 74804) according to the manufacturer's instructions. RNA quantity was assessed with a NanoDrop 2000 spectrometer (ThermoFisher), while quality was confirmed via electrophoresis of samples in a 1% bleach gel as previously described (12). RNA was used to generate cDNA with a First Strand Superscript III cDNA synthesis kit (ThermoFisher, 18080051) according to the manufacturer's instructions and with equal amounts of starting RNA. Quantitative-PCR was performed with the validated BioRad PCR primers using SsoAdvanced Universal green Supermix (BioRad, 1725271, detailed in **Table S1**). Fold changes in gene

expression were determined relative to a defined control group using the $2^{-\Delta\Delta Ct}$ method or by z-score, with β-Actin or HPRT used as housekeeping genes.

Immunoblot analysis

Mouse primary GBM cell lines were treated with the following conditions: (1) 100 pM IL-1β (R&D Systems, 401-ML/CF) for 10, 30, and 60 minutes. (2) 10 and 20 µM BAY 11-7082 (Millipore Sigma, 196870) for 24 and 48 hours, and (3) 10 and 20 µM Cardamonin (Tocaris, 2509) for 24 and 48 hours. Cells were washed 2 x with ice-cold PBS and scraped into RIPA buffer (50 mM Tris, pH 8.0, 150 mM NaCl, 5 mM EDTA, 1% NP40 and 0.5% deoxycholate, 10 mM NaF, 1 mM sodium orthovanadate with complete protease inhibitor cocktail (Roche) as describe previously, incubated on ice for 30 min, centrifuged, and protein concentrations were determined using a BCA kit (Pierce, 23227). Cell lysates (10-50 µg) were subjected to SDS-polyacrylamide gel electrophoresis on a 4-20% precast gradient gels (BioRad, 5678094) and proteins were transferred to nitrocellulose membranes (Bio-Rad, 162-0112). After transfer, membranes were incubated in 5% Nonfat Dry Milk (Cell Signaling Technology, 9999) in 1 x TBST (Cell Signaling, 9997) for 1 hour. Membranes was incubated for 16 hours at 4°C with primary antibody: p-NF-kB p65 (1:1000), NF-kB (1:1000), p-IkB-alpha (1:1000), and IkB-alpha primary antibodies (1:1000); all antibodies were from the NF-kB Pathway Sampler kit (Cell Signaling, 9936S). p-STAT3 (1:5000, 9145) and STAT3 (1:10,000, 12630) were also from Cell Signaling. β-actin was from Abgent (1:5000, ABIN1842939) or (1:50,000, Cell Signaling, 3700). Immunodetection was performed with anti-rabbit horseradish-peroxidase-conjugated secondary antibody (1:1000) and anti-mouse horseradish–conjugated secondary antibody (1:1000); secondary antibodies were from NF-kappa B Pathway Sampler kit (Cell Signaling, 9936S). Immunodetection was performed with Chemiluminescent HRP Antibody Detection Reagent (Denville Scientific INC, E2400).

Human tissue samples and pathological appraisal

Archived formalin-fixed, paraffin-embedded (FFPE) human GBM samples and de-identified clinical information were provided by Emory University (IRB Study 18-177) and patient information is included in our published manuscript (13). Fresh tumor tissues used for ELISA quantification were collected at Mount Sinai Hospital through the biorepository, under IRB-approved protocols (18-00983). All patient samples were de-identified. Ten randomly selected tumor samples were included in this study, with de-identified age, sex, and molecular information summarized in **Table S3**. "Normal appearing" brain tissues away from the tumor mass, obtained during surgical resection, from three patient samples were used as controls. Board-certified neuropathologists diagnosed and graded both the human tumor tissues and murine samples according to the 2016 World Health Organization Classification of Tumors of the Central Nervous System (14). Gene expression profiling to determine transcriptional subtypes was performed using targeted genome sequencing (Foundation One/Sema4).

TCGA analysis

U133 Microarray data for the GBM (TCGA, provisional) dataset were downloaded from cBioPortal (https://www.cbioportal.org) in August 2021 and sorted into subtypes based upon a proprietary key. G-CIMP-positive tumors were excluded from analysis. We included 372 patient samples for which covariate information (survival information, age, and gender) was available. All of the

TCGA dataset samples analyzed in this manuscript were IDH-wildtype malignant gliomas, consistent with the diagnosis of (IDH-wildtype) Glioblastoma according to the WHO 2021 CNS tumor classification. We have now added this information to the methods section. Cox Proportional Hazard Models were fitted in R using age and gene expression as continuous covariates, and gender as a binary variable. Forest plots were done using the function *ggforest*.

Tissue processing and immunohistochemistry

Archived FFPE human GBM samples were sectioned at 5 µm thickness, slide-mounted, and stored at -80°C until use. To process mouse tumor tissues, animals at humane endpoint were anesthetized with an overdose of ketamine/xylazine mix and transcardially perfused with ice-cold sterile Ringer's solution. Brains were removed and processed according to the different applications. For H&E tumor validation and immunohistochemistry staining, brains were fixed in 10% neutral buffered formalin for 72 hours at room temperature (RT), processed in a tissue processor (Leica, TP1050), embedded in paraffin, sectioned (5 µm), and slide mounted.

Immunohistochemistry staining was performed on either Discovery XT platform (Ventana Medical Systems) or Leica Bond Rx (Leica). Primary antibodies used in this study include: anti-IBA-1 (1:1500, Wako,019-19741), anti-phosphorylated Histone 3 (1:200, Millipore, 06-570), anti-CD44 (1:100, BD Pharmingen, 550538). Digital images of the slides were acquired by using a Nanozoomer 2.0HT whole-slide scanner (Hamamatsu Photonic K.K) and observed offline with NDP view2 software (Hamamatsu). Image analysis was performed using Fiji (NIH).

Immunofluorescence

Human GBM 5 µm FFPE sections were stained with anti-IBA1 (1:500, Wako, 019-19741) and anti-IL-1β (NCI preclinical repository, biological resource branch, 32D). Secondary antibodies conjugated to Alexa-Fluor dyes (555 nm, 647 nm from Invitrogen) at a dilution of 1:500 in PBS/2% BSA were applied. DAPI (Sigma, D9542) was used for nuclear counterstaining. Autofluorescence from erythrocytes was recorded in the green emission channel. Fluorescence images were taken on an Olympus FV1000 confocal microscope and analyzed with FIJI software (NIH).

Enzyme-linked immunosorbent assay

Cell lysates for enzyme-linked immunosorbent assay (ELISA) were collected via sonication of cells in lysis buffer supplemented with protease and phosphatase inhibitors. Tissue lysates were collected via mechanical homogenization in RIPA lysis buffer as described above followed by sonication. Protein concentrations were determined using a Bradford protein assay (Bio-Rad, 5000001) according to the manufacturer's instructions. ELISAs were performed for hIL-1β (R&D, DY201-05), mIL-1β (R&D, DY401-05), CCL2 (R&D, DY479), CCL7 (Boster Bio, EK0683), CCL8 (R&D, DY790), CCL12 (R&D, MCC120), and VEGF (R&D, MMV00) on cell lysates and cell supernatants according to the manufacturer's instructions.

Flow Cytometry and spectral flow cytometry

Mice at humane endpoint were anesthetized with an overdose of ketamine/xylazine mix and transcardially perfused with ice-cold sterile Ringer's solution (catalog number). Initial steps of the enzymatic dissociation of the tumors are the same as described above, except 0.5% collagenase

D (Sigma, 11088858001) and DNase I (Roche, 11284932001) were used in place of papain. Single-cell suspensions were passed through 70 µm cell strainers, centrifuged, and resuspended in 30% Percoll (GE Healthcare, 17-0891-01) solution containing 10% FBS (Hyclone SH30396.03). Cells were separated by centrifugation at 800*g* for 15 minutes at 4°C. The supernatant was carefully removed to discard debris and lipids. The cells were then washed in cold PBS and resuspended in RBC lysis buffer (BioLegend, 420301) for 1 min at 37°C. Cells were transferred to an Eppendorf tube and washed once with FACS buffer (DPBS with 0.5% BSA) and blocked with 100 µl of 2x blocking solution (2% FBS, 5% normal rat serum, 5% normal mouse serum, 5% normal rabbit serum, 10 µg/ml anti-FcR (BioLegend, 101319) and 0.2% NaN_3 in DPBS) on ice for 30 minutes. Cells were then stained with primary antibodies (**Table S2**) on ice for 30 minutes and washed with PBS. The cells were subsequently incubated in 100 µl viability dye (Zombie UV, BioLegend, 1:800) at room temperature for 20 min. The cells were washed and fixed with fixation buffer (eBioscience, 00-5123-43, 00-5223-56) for 30 min. Cells stained with the cocktail of antibodies examined myeloid lineage are set aside in the fridge until loading to the cytometer. Cells stained for the lymphoid panel were then permeabilized with a permeabilization buffer (eBioscience, 00-8333-56) before the intracellular markers were stained. The cells were washed and stored in fridge till analysis. Antibodies used in this study include are listed in **Table S2**. All data were collected on a BD LSR II flow cytometer or Cytek Aurora spectral flow cytometer. Data were analyzed offline using FlowJo 10 software (Tree Star Inc.).

Fluorescence-activated cell sorting (FACS) was used to enrich tumor-associated microglia, BMDM or glioma cells allowing downstream quantitative PCR analysis. Mice at humane endpoint were anesthetized with an overdose of ketamine/xylazine mix and transcardially perfused with ice-cold sterile Ringer's solution. Tumors are dissected and dissociated with papain as described above. The cells were stained with a cocktail of antibodies as previously described (15) and were sorted on a Sony SH800 cell sorter. Normal microglia was similarly sorted, except naïve mouse brains were used. Microglia are defined as $CD45^{Lo}CD11b^{+}Ly6c^{Neg}Ly6g^{Neg}$; BMDM are $CD45^{Hi}CD11b^{+}Ly6c^{+}Ly6g^{Neg}$; Glioma cells are negative for all markers.

Single-cell RNA-seq and data analysis

Mice at humane endpoint were anesthetized with an overdose of ketamine/xylazine mix and transcardially perfused with ice-cold sterile Ringer's solution. Single cell suspensions of the tumors were obtained by papain dissociation as described above. Viability of single cells was assessed using Trypan Blue staining, and debris-free suspensions of >80% viability were deemed suitable for single cell RNA Seq. Single cell RNA Seq was performed on these samples using the Chromium platform (10X Genomics) with the 3' gene expression (3' GEX) V3 kit, using an input of ~10,000 cells. Briefly, Gel-Bead in Emulsions (GEMs) were generated on the sample chip in the Chromium controller. Barcoded cDNA was extracted from the GEMs by Post-GEM RT-cleanup and amplified for 12 cycles. Amplified cDNA was fragmented and subjected to end-repair, poly A-tailing, adapter ligation, and 10X-specific sample indexing following the manufacturer's protocol. Libraries were quantified using Bioanalyzer (Agilent) and QuBit (ThermoFisher) analyses and were sequenced in paired end mode on a NovaSeq instrument (Illumina) targeting a depth of 50,000-100,000 reads per cell.

Raw fastq files were aligned to mouse genome reference mm10 customized to include the Rfp sequence, using CellRanger v5.0.0 (10X Genomics). Count matrices filtered by CellRanges algorithm were further filtered by discarding cells with either < 400 genes, < 1000 UMI (unique molecular identifier), or > 25% mitochondrial genes expressed. Data was processed and analyzed using R package Seurat v4.0.5. Normalization was performed using NormalizeData function with normalization.method = 'LogNormalize'. Dimensionality reduction was computed on the top 2,000 variable features using FindVariableFeatures, ScaleData and RunPCA functions. UMAPs were generated using the top 15 PCs. For subclustering the immune compartment, we used R package Harmony v0.1 to mitigate batch effects driven by technical variation between replicates. *De novo* clustering using the Louvain algorithm was applied at different resolutions (0.2; 0.8; 2; 5) on the KNN graph space. For high-level annotation, cell types were identified in an iterative and semi-supervised fashion by assigning *de novo* discovered clusters to cell types based on expression of known marker genes that define each cluster. Annotation of cell subtypes at a lower-level was performed in a similar manner as for the high-level and further aided by *de novo* marker discovery using the Seurat FindMarkers function and Wilcoxon Rank Sum differential expression test. To identify doublet-enriched clusters we looked for clusters of cells displaying expression of canonical markers for two or more different cell types and higher number of genes/UMI; such clusters were removed from further analysis. Cell-level proliferation analysis was carried out with Seurat function CellCycleScoring using a list of murine cell cycle genes. Cells were then assigned as 'cycling' or 'non-cycling' if either their S-phase score or G2M-score was above a threshold of 0.05 for the tumor compartment and 0.1 for the immune compartment.

Identification of modules of co-expressed genes was carried out using the R package scWGCNA (https://github.com/smorabit/scWGCNA) by first computing metacells of 100 cells (k=100) using function construct_metacells. To identify modules, function blockwiseConsensusModules was called with following parameters: softPower=12, deepSplit=3, mergeCutHeight = 0.25. Only the top 2,000 variable genes were used.

To generate the pie charts shown in Supplementary Figures 15 and 16, we tallied the total counts of the molecules of interest and traced their source to either malignant cells or non-malignant cells. The total counts obtained from the tumor compartment were then divided by the numbers of tumor cells expressing these molecules. Similarly, this ratio was also obtained from the non-malignant compartment. These two ratios were subsequently used to create the pie chart.

Hematoxylin and eosin tumor volume reconstruction

Mice were sacrificed 25 days post-tumor cell transplantation with an overdose of ketamine and xylazine and perfused with 4% paraformaldehyde in PBS. The brain was carefully extracted and incubated in 4% paraformaldehyde in PBS for 24 hours following a 72-hour incubation in 30% sucrose in PBS. The brain was then embedded in O.C.T. compound (VWR, 25608-930) and frozen on dry ice. The entire brain was then serially sectioned on a cryostat (Leica) set to cut 30 µm sections. Every tenth section was collected and mounted on a slide for automated hematoxylin and eosin staining as described above. The slides were scanned at 20x magnification with a whole-slide scanner (Hamamatsu). Tumor area in each section was determined in a blinded

fashion in NDP.view2 and multiplied by the thickness of ten slices. The resulting volumes for the slides of each tumor were then summed, producing an estimation of the total volume.

Cannula installation and drug administration in tumor-bearing mice

Mice were anaesthetized with a ketamine (100 mg/kg) and xylazine (10 mg/kg) cocktail, their heads shaved, and they were placed into a stereotactic device with a three-axis micromanipulator. The burr hole used to inject the RCAS virus was re-probed with a compact drill. A guide cannula (Plastics One) was implanted into the brain through the craniotomy and fixed in place with dental acrylic. A matching stylet or "dummy-cannula" was then screwed onto the guide cannula to prevent back flowing of the CSF or environmental debris from entering the guide cannula. Once the dental acrylic is cured, the skin around the pedestal of the guide cannula was sealed with Gluture liquid stitches (World Precision Instruments, 503763).

On the day of the infusion, the mice were anesthetized with inhalable isoflurane at 3% (volume/volume in pure oxygen) with a nebulizer. The dummy cannula was removed, and a Hamilton syringe mounted on a stereotactic frame was lowered into the guide cannula. Up to 2 µl of vehicle (artificial CSF, Harvard Instrument, containing isotype antibody, BE0091, BioXcell), IL-1RA (US Biological Corporation, I7663-62E), or purified anti-IL-1β IgG (BioXcell, BE0246) were injected into the lateral ventricle over 1 min. Once the injection was completed, the dummy cannula was screwed back, and mice were returned to their home cages.

***In situ* proteomics by NanoString GeoMx assay**

Formalin-fixed, paraffin-embedded sections of 5 µm thickness were placed onto positively-charged slides (ThermoFisher). The GeoMx Immune Cell Profiling Panel Mouse Protein Core with a 20-gene selection was used for nCounter readout along with morphology markers pan-cytokeratin (Alexa-488), leukocyte common antigen CD45 (Alexa-647), and DAPI (nuclei) staining for immunofluorescence labelling. Six circular ROIs 300 µm in diameter were utilized for targeted spatial transcriptomics using the GeoMx platform (NanoString, Seattle, WA). ROIs were generated in the tumor-rich region of the tissue sections, particularly adjacent to perivascular or perinecrotic spaces. Once digital spatial profiling data was completed, the samples were pooled for nCounter readout in RCC format.

RCC files generated from the nCounter readout were plugged into the digital spatial profiling software for initial QC and further analysis. ROIs with nuclei count less than 20 were excluded from further analysis. Data was subsequently normalized against GAPDH and Histone H3, two housekeeping genes. The normalized data were used for comparisons between treatment groups and vehicle controls, and to generate corresponding heatmaps.

STable 1. qPCR Primers Used in Study. The Bio-Rad qPCR primers used in the study are listed with their catalog numbers.

Primer	Bio-Rad Catalog Number
Abcg2	qMmuCID0009104
Actb	qMmuCED0027505
Aif1	qMmuCED0046745
Ascl1	qMmuCED0044820
Ccl11	qMmuCED0044849
Ccl12	qMmuCED0061017
Ccl2	qMmuCED0048300
Ccl7	qMmuCED0049027
Ccl8	qMmuCED0003781
Ccr1	qMmuCID0006862
Cd44	qMmuCID0025677
Cebpb	qMmuCED0050360
Chi3l1	qMmuCID0015758
Dll3	qMmuCID0023659
Gfap	qMmuCID0020163
Hprt	qMmuCED0045738
Il1a	qMmuCID0005637
Il1b	qMmuCID0005641
Met	qMmuCID0017026
Mgmt	qMmuCID0009593
Olig2	qMmuCED0003760
Rps6ka3	qMmuCID0006067
Serpine1	qMmuCID0027303
Sox2	qMmuCED0051857
Stat3	qMmuCED0044698
Taz	qMmuCID0020469
Tgfb1	qMmuCID0017320
Yap1	qMmuCID0005990

STable 2. Antibodies used in the study.

Antibody	Application	Specificity	Manufacturer	Catalog Number
IBA1	IHC/IF	Human, Mouse	Wako	019-19741
IL-1β	IF	Human, Mouse		
IL-1β	In vivo injection	Mouse	BioXcell	CST 3169S
Phospho-Histone 3	IHC	Mouse	Millipore	06-570
CD31	IHC	Mouse	Dianova	DIA-310
CD44	IHC	Mouse	BD Pharmingen	550538
CD31	IHC	Human	Dako	M0823
p-NF-κB p65	Western blot	Mouse	Cell signaling	9936S
NF-κB	Western blot	Mouse	Cell signaling	9936S
p-IκB-a	Western blot	Mouse	Cell signaling	9936S
IκB-a	Western blot	Mouse	Cell signaling	9936S
β–Actin	Western blot	Mouse	Abgent	ABIN1842939
CD45-APC	Flow Cytometry	Mouse	BioLegend	103112
CD45-FITC	Flow Cytometry	Mouse	BioLegend	103107
CD45-V450	Flow Cytometry	Mouse	BD Biosciences	560501
B220-BV605	Flow Cytometry	Mouse	BioLegend	103243
CD101-APC	Flow Cytometry	Mouse	Invitrogen	17101180
CD103-BUV395	Flow Cytometry	Mouse	BD Biosciences	748253
CD11b-PerCP-Cy5.5	Flow Cytometry	Mouse	BD Biosciences	550993
CD11c-APC	Flow Cytometry	Mouse	BD Biosciences	550281
CD24-BUV496	Flow Cytometry	Mouse	BD Biosciences	
CD3-PE-dazzle	Flow Cytometry	Mouse	BioLegend	100348
CD4-APC-Cy7	Flow Cytometry	Mouse	BioLegend	100526
CD49d-PE-dazzle	Flow Cytometry	Mouse	BioLegend	103625
CD8-BV510	Flow Cytometry	Mouse	BioLegend	100752
CX3CR1-BV650	Flow Cytometry	Mouse	BioLegend	149033
CXCR2-PE	Flow Cytometry	Mouse	BioLegend	149609
F4/80-BV711	Flow Cytometry	Mouse	BioLegend	123147
Foxp3-FITC	Flow Cytometry	Mouse	Invitrogen	11-5773-82
GrzmB-PE	Flow Cytometry	Human, Mouse	Invitrogen	12-8899-41
IA/IE-Alex700	Flow Cytometry	Mouse	BioLegend	107622
Ly6C-PE-Cy7	Flow Cytometry	Mouse	BD Biosciences	560593
Ly6c-PE-Cy7	Flow Cytometry	Mouse	BD Biosciences	560593
Ly6G-V450	Flow Cytometry	Mouse	BD Biosciences	560603
Ly6g-V450	Flow Cytometry	Mouse	BD Biosciences	560603
NK1.1-BV711	Flow Cytometry	Mouse	BD Biosciences	740663
PD-L1-BV605	Flow Cytometry	Mouse	BD Biosciences	745135

PD1-BV785	Flow Cytometry	Mouse	BioLegend	135225
Tim-3-PE-Cy7	Flow Cytometry	Mouse	Invitrogen	25-5870-82

STable 3. Patient information.

Sample #	Tumor diagnosis	Sex	Age	Sub-classification	Molecular driver
16302 (A2)	GBM, IDH-WT	Female	74	MES	NF1 mutation
21810	GBM, IDH-WT	Female	66	CL	EGFR amplified, TERT promoter c-146C>T, TP53 p.Y220D missense; PTEN loss; CDKN2A/B loss
24286	GBM, IDH-WT	Male	46	CL	EGFR amplified; EGFR G598V - subclonal, EGFRvIVa† (ex 24-27 del); ATM splice site 3576G>A; KDR R961W; CDKN2A/B loss; MTAP loss; TERT promoter -124C>T; VUS: CARD11 A687V; CEBPA V287G; CRKL T213A; GATA4 R318T; MAP3K1 T1511I; MTAP M169V; NTRK1 G18E; NTRK2 S167Y; PTEN F154I; SETD2 V932I;
27419	GBM, IDH-WT	Female	56	MES	NF1 splice site 2850+1G>A; CDKN2A loss; PTEN M199del; TERT -146C>T; TP53 T211_H214del; MS-Stable; 4 muts/ MB
29282	GBM, IDH-WT	Female	58	CL	EGFR amplified; EGFR p.A289V; PTEN p.K332Tfs*8; TERT c.-124C>T
31100	GBM, IDH-WT	Male	75	MES	CDKN2A loss; MTAP loss; NF1 N2387_F2388de; NF1 K1444E; PTEN loss; PTPN11 Y63C; TERT promoter -146C>T; MS-Stable; 1 mut/Mb;
11849	GBM, IDH-WT	Female	74	PN	PDGFRa and KIT amplifications
13629	GBM, IDH-WT	Male	69	unknown	BRAF c.1390G>C (G464R)
16499	GBM, IDH-WT	Female	73	unknown	NA
29162	GBM, IDH-WT	Female	66	unknown	TP53 mutation

References

1. Chen Z, Herting CJ, Ross JL, Gabanic B, Puigdelloses Vallcorba M, Szulzewsky F, et al. Genetic driver mutations introduced in identical cell-of-origin in murine glioblastoma reveal distinct immune landscapes but similar response to checkpoint blockade. *Glia.* 2020;68(10):2148-66.
2. Herting CJ, Chen Z, Pitter KL, Szulzewsky F, Kaffes I, Kaluzova M, et al. Genetic driver mutations define the expression signature and microenvironmental composition of high-grade gliomas. *Glia.* 2017;65(12):1914-26.
3. Herting CJ, Chen Z, Maximov V, Duffy A, Szulzewsky F, Shayakhmetov DM, et al. Tumour-associated macrophage-derived interleukin-1 mediates glioblastoma-associated cerebral oedema. *Brain.* 2019;142(12):3834-51.
4. Shornick LP, De Togni P, Mariathasan S, Goellner J, Strauss-Schoenberger J, Karr RW, et al. Mice deficient in IL-1beta manifest impaired contact hypersensitivity to trinitrochlorobenzone. *J Exp Med.* 1996;183(4):1427-36.
5. Horai R, Asano M, Sudo K, Kanuka H, Suzuki M, Nishihara M, et al. Production of mice deficient in genes for interleukin (IL)-1alpha, IL-1beta, IL-1alpha/beta, and IL-1 receptor antagonist shows that IL-1beta is crucial in turpentine-induced fever development and glucocorticoid secretion. *J Exp Med.* 1998;187(9):1463-75.
6. Di Paolo NC, Shafiani S, Day T, Papayannopoulou T, Russell DW, Iwakura Y, et al. Interdependence between Interleukin-1 and Tumor Necrosis Factor Regulates TNF-Dependent Control of Mycobacterium tuberculosis Infection. *Immunity.* 2015;43(6):1125-36.
7. Franklin KBJ, and Paxinos G. *The mouse brain in stereotaxic coordinates.* San Diego: Academic Press; 1997.
8. Roy J. Primary microglia isolation from mixed cell cultures of neonatal mouse brain tissue. *Brain Res.* 2018;1689:21-9.
9. Reilly KM, Loisel DA, Bronson RT, McLaughlin ME, and Jacks T. Nf1;Trp53 mutant mice develop glioblastoma with evidence of strain-specific effects. *Nat Genet.* 2000;26(1):109-13.
10. Gursel DB, Connell-Albert YS, Tuskan RG, Anastassiadis T, Walrath JC, Hawes JJ, et al. Control of proliferation in astrocytoma cells by the receptor tyrosine kinase/PI3K/AKT signaling axis and the use of PI-103 and TCN as potential anti-astrocytoma therapies. *Neuro Oncol.* 2011;13(6):610-21.
11. Pan Y, Smithson LJ, Ma Y, Hambardzumyan D, and Gutmann DH. Ccl5 establishes an autocrine high-grade glioma growth regulatory circuit critical for mesenchymal glioblastoma survival. *Oncotarget.* 2017;8(20):32977-89.
12. Aranda PS, LaJoie DM, and Jorcyk CL. Bleach gel: a simple agarose gel for analyzing RNA quality. *Electrophoresis.* 2012;33(2):366-9.
13. Kaffes I, Szulzewsky F, Chen Z, Herting CJ, Gabanic B, Velazquez Vega JE, et al. Human Mesenchymal glioblastomas are characterized by an increased immune cell presence compared to Proneural and Classical tumors. *Oncoimmunology.* 2019;8(11):e1655360.
14. Louis DN, Perry A, Wesseling P, Brat DJ, Cree IA, Figarella-Branger D, et al. The 2021 WHO Classification of Tumors of the Central Nervous System: a summary. *Neuro Oncol.* 2021;23(8):1231-51.

15. Chen Z, Feng X, Herting CJ, Garcia VA, Nie K, Pong WW, et al. Cellular and Molecular Identity of Tumor-Associated Macrophages in Glioblastoma. *Cancer Res.* 2017;77(9):2266-78.
